# The genus *Amegilla* (Hymenoptera, Apidae, Anthophorini) in Australia: a revision of the subgenus *Asaropoda*

**DOI:** 10.3897/zookeys.908.47375

**Published:** 2020-02-03

**Authors:** Remko Leijs, James Dorey, Katja Hogendoorn

**Affiliations:** 1 South Australian Museum, North Terrace, Adelaide, SA 5000, Australia South Australian Museum Adelaide Australia; 2 School of Biology, Flinders University, Adelaide, SA 5001, Australia Flinders University Adelaide Australia; 3 School of Agriculture, Food and Wine, The University of Adelaide, SA 5005, Australia The University of Adelaide Adelaide Australia

**Keywords:** teddy bear bee, taxonomic revision, mtDNA phylogeny, new species

## Abstract

The species in the subgenus Amegilla (Asaropoda) are revised. Species delineation was decided based on diagnostic morphological characters as well as an incomplete phylogeny based on mitochondrial cytochrome oxidase 1 sequence data. Strong support was obtained for separating the Australian species of *Amegilla* into the three subgenera previously proposed on the basis of morphology. The subgenus Asaropoda was found to comprise 21 species, including ten new species: *A. albiclypeata* Leijs, **sp. nov.**, *A. aurantia* Leijs, **sp. nov.**, *A. batleyi* Leijs, **sp. nov.**, *A. crenata* Leijs, **sp. nov.**, *A. griseocincta* Leijs, **sp. nov.**, *A. incognita* Leijs, **sp. nov.**, *A. nitidiventris* Leijs, **sp. nov.**, *A. scoparia* Leijs, **sp. nov.**, *A. xylocopoides* Leijs, **sp. nov.**, and *A. youngi* Leijs, **sp. nov.** The subspecies *A. preissi frogatti* is raised to species level, and 16 new synonymies are proposed. Keys to the species of both sexes and descriptions or redescriptions are provided. Distribution maps, data on flower visitation and phenology are given.

## Introduction

The Australian members of the genus *Amegilla* (Hymenoptera, Apidae, Anthophorini) have been classified into three groups of species (Rayment 1951), which were formalised by Brooks (1988) as the subgenera *Asaropoda* and *Notomegilla*, which are endemic to Australia and Papua New Guinea, and *Zonamegilla*, which has a wide distribution throughout Eurasia, SE Asia, and Australia (Michener 2007). However, because of the difficulty of separating females of different species, Michener (2007) chose not to recognise subgenera, but acknowledged that the names might be useful to define species-groups within this very large genus.

The taxonomy of the genus *Amegilla* in Australia has been summarised by Leijs et al. (2017), in a revision of the species in the subgenera *Notomegilla* and *Zonamegilla*. Here, we revise the species in the subgenus Asaropoda.

The first A. (Asaropoda) species, Amegilla (Asaropoda) bombiformis was described more than 165 years ago by Smith (1854), followed by A. (A.) scymna circa 40 years later (Gribodo 1894). A further five species and a subspecies were described by Cockerell and Friese in the early 1900’s (Cockerell 1905, 1910, 1914, 1926, 1929, Friese 1911). Rayment (1931) described six additional species from bees in the collections of the Western Australian Museum and the Agriculture Department, Perth. This work was followed by a publication in 1951, titled “A critical revision of the genus *Asaropoda* by new characters” (Rayment 1951), which added another eight species. This ‘critical revision’ was instigated by Cockerell (1929) who suggested the likelihood of multiple species within *A. bombiformis*. Unfortunately, one of the new characters used for the diagnosis of these species, the sculpture of the pygidial plate, appears to be unreliable because it is subject to wear with age. Although his descriptions were well illustrated, especially of images of dissected metasomal sterna and genitalia, diagnostic differences between these species with respect to these characters were not discussed. The lack of keys made proper identification of the species difficult. One of Rayment’s species, A. (A.) albigena, was renamed by Michener (1965), A. (A.) albigenella, because the name was already used for another *Amegilla* species. Brooks (1988) described two new Australian species associated with his beautifully illustrated work on the systematics and phylogeny of Anthophorine bees. He also synonymised two of the previously named species, alas without explanation. Cardale (1993) lists 26 A. (Asaropoda) species, of which one, *A. alpha*, appeared to belong to the subgenus Zonamegilla (Leijs et al. 2017), and together with the latest addition, A. (A.) paracalva (Brooks, 1993), 26 names for A. (Asaropoda) were recognised.

Within the subgenus Amegilla (Asaropoda), the species *A. bombiformis* was given the common name ‘teddy bear bee’ because of its fluffy red-brown habitus (Rayment 1935). This species is common along the East coast of Australia and appears on many images published on the internet. Besides the ‘blue banded bees’, Amegilla (Zonamegilla), the ‘teddy bear bees’ are probably the best-known native bees to the Australian public. However, little is known about their lifecycle and biology. In that respect, the best studied species is Dawson’s burrowing bee, *A. dawsoni*. This species is among Australia’s largest bee species and has extensive nesting aggregations on mud flats in central coastal Western Australia. The biology of *A. dawsoni* was first described by Houston (1991). The species has males in two distinct size classes, which are associated with different mating strategies. Large males wait at nesting sites for newly emerging virgin females and fight for mates, while small males mainly patrol flowers near the nest sites for virgin females. Such male size dimorphism is relatively rare in bees (see references in Alcock 1996) and the maintenance and understanding of such a biological system poses interesting behavioural, evolutionary and genetic questions. Several researchers published about *A. dawsoni* between 1996 and 2006. Alcock (1996) tested a number of hypotheses as to why female bees would produce a mix of large and small males but did not find sufficient support for any of them. Alcock (1997) described the size related male mating strategies. Simmons et al. (2000) showed that there was no sperm competition between large and small males and that females only mate once. Simmons et al (2003) looked at cuticular hydrocarbons with respect to mate attraction and found that females become unattractive to males soon after mating. Tomkins et al. (2001) and Alcock et al (2005) showed that female bees produce small males towards the end of the flight season when flower resources become scarce, which was confirmed using genetic methods by Beveridge et al. (2006a). Alcock et al (2006) showed that larger females do not have a fitness advantage over smaller females. Finally, Beveridge et al. (2006b) used population genetic methods to indicate that *A. dawsoni* should be considered a single panmictic population across its distribution. Unfortunately, even after ten years and study by multiple researchers the male size dimorphism conundrum in *A. dawsoni* remains incompletely resolved (Alcock et al. 2005).

Alcock et al. (2010) described the mating behaviour of *A. calva*, which is similar to *A. dawsoni*, apart from the observation that males appear in a single size class. Mating and nesting behaviour was only recorded from a small number of additional *Asaropoda* species. Rayment (1935, 1951) and Cardale (1968) describe that *A. bombiformis* makes nests in walls of mudbrick or rammed earth and frequently nest in soil under houses.

Here we present a revision of the species in the subgenera *Asaropoda* based on examination of the majority of the type material and supported by molecular phylogenetics based on mitochondrial DNA.

## Materials and methods

### Specimens examined

This study is based on examination of 1466 museum specimens, including most type specimens. The following acronyms are used in the database of the examined specimens (available as supplementary information associated with this paper):

**ABTC** Australian Biological Tissue Collection at SAMA, Adelaide

**AM** Australian Museum, Sydney

**ANIC** Australian National Insect Collection, Canberra

**NHMUK** The Natural History Museum, London, United Kingdom

**MAGNT** Museum and Art Gallery Northern Territory, Darwin

**MSNG** Civic Museum of Natural History, Genoa, Italy

**MV** Museum of Victoria Entomology, Melbourne

**QM** Queensland Museum, Brisbane

**SAMA** South Australian Museum, Adelaide

**UQIC** University of Queensland Insect Collection, Brisbane, housed at the QM

**WADA** Department of Primary Industries, Perth

**WAM** Western Australia Museum, Perth

**WINC** Waite Insect and Nematode Collection, Adelaide University, Waite Campus, Adelaide

**ZMB** Museum für Naturkunde, Humboldt-Universität, Berlin, Germany

Additional fresh specimens were collected throughout Australia by the authors and other collectors acknowledged below. Field collected specimens were killed and preserved in absolute ethanol to allow DNA extraction at a later stage. Ethanol preserved specimens, as well as extracted DNA, are kept in the Australian Biological Tissue Collection (**ABTC**) at the South Australian Museum. DNA-voucher specimens are kept in the Entomology Collection of the South Australian Museum. Locality data, voucher numbers and GenBank Accession numbers of specimens used in the molecular analyses are available in the supporting information. With the species descriptions only the data of the type specimens are given, the full data of all examined specimens can be found in the supplementary information Table S1.

### *Taxonomic methods*

Genitalia were dissected from male museum specimens, after relaxing in a humid container with added chromocresol as fungicide for up to three days. Genitalia were treated with 10% cold sodium hydroxide for 24 hours, glacial acetic acid for approximately one hour and stored in glycerol to facilitate the study of morphology. For species descriptions the terminology used by Michener (2007) was followed and we adopted the morphological character set used by Brooks (1988, 1993) for his descriptions of *A. houstoni*, *A. epaphrodita*, and *A. paracalva*. We use the term paraclypeal area for the lower part of the para-ocular area that lies between the eye and the clypeus. The terminology for integument sculpture, grades of pit and pubescence density and pit size follows Houston (1975), reproduced in Leijs et al. (2018). Integument sculpture was observed using 40× magnification and YK-B144T LED ring elimination.

A Leica stereomicroscope with auto-montage imaging stacking software was used to obtain high-resolution diagnostic images of all species. A Canon EOS 5DSR camera attached to a Nikon Eclipse 50i compound microscope and Zerene Stacker were used to image male genitalia and metasomal sterna seven and eight.

The following abbreviations are used in the identification key and species descriptions:

**T1, T2** etc. for first, second metasomal tergum etc.;

**S1, S2** etc. for first, second metasomal sternum etc.;

**F1, F2** etc. for first, second flagellar segment etc.

### DNA methods

DNA extraction, PCR amplification and sequencing were performed as described in Cooper et al. (2002). Two regions of the mitochondrial genome were amplified. An 822 bp region of the 3’ end of the cytochrome oxidase subunit 1 (CO1) gene was amplified using primers M202 (forward, 5’-CAA CAT TTA TTT TGA TTT TTT GG-3’, alias Jerry, Simon et al. 1994) and M70 (reverse, 5’-TCC ATT GCA CTA ATC TGC CAT ATT A-3’) (UEA9 and 10, Lunt et al. 1996), as well as a 648 bp fragment just upstream of the previous fragment of CO1 using primers M414 (5’-GGT CAA CAA ATC ATA AAG ATA TTG G-3’) and M423 (5’-TAA ACT TCA GGG TGA CCA AAA AAT CA-3’) (LCO1490 and HCO2198, Folmer et al. 1994). ChromasPro version 1.34 (Technelysium Pty Ltd, Tewantin, QLD, Australia) was used to edit chromatogram files, to determine consensus sequences from both strands, and to align sequences across specimens. New sequences are lodged with GenBank accession numbers MN908567-MN908578.

Some of the specimens treated here were also submitted to BOLD (Barcode of Life Database) for DNA barcoding using the cytochrome c oxidase subunit 1 gene. Specimen details, including DNA sequence, collection dates and locality information can be accessed in BOLD under the project Australian Bee Survey, e.g., http://www.boldsystems.org/index.php/Public_RecordView?processid=AUSBS304-13. AUSBS-numbers are presented under material examined.

### *Phylogenetic analyses*

Phylogenetic analyses of aligned sequence data were carried out using PAUP* version 4.0b8 (Swofford 2001) and MRBAYES v.3.2.4 (Huelsenbeck and Ronquist 2001). PAUP* was used for generating and editing data matrices, error proofing using neighbour joining runs, as well as for analyses of uncorrected sequence divergence. Bayesian analyses, using MRBAYES were performed by applying unlinked data partitions for each of the codons for the CO1 gene and using a general time reversible model of sequence evolution with invariable sites and gamma distributed rates across sites. Tracer v1.4 (Rambaut and Drummond 2007) was used to make sure that the effective sample size (ESS) of the parameters during the Bayesian runs were larger than 100.

MRBAYES allowed the application of relaxed molecular clock methods in order to obtain estimates of node divergence times. Because fossils are unavailable for *Amegilla* species, a mean rate of 0.0105 substitutions per site per million years for COI (Papadopoulou et al. 2010) was used as prior with an uncorrelated log-linear relaxed molecular clock. We are aware of the limitations of using a ‘borrowed’ clock rate, but because Papadopoulou’s rate is modelled using a large (> 30) number of independent rate calibrations from various insect taxa, for the moment, we consider it the best option to use for our divergence estimates. The analyses were performed using three million generations with two independent runs each of four simultaneous chains, sampling trees every 100 generations, until all parameters had reached their ESS, and the potential scale reduction parameter was approximately one for all parameters, indicating that the Bayesian runs had converged and that a sufficient sample of the posterior distribution had been obtained. Parameter estimation and calculations of the > 50 % posterior probability consensus tree was done after discarding the first 25 % of the saved trees.

## Results and discussion

### A revision of *Amegilla* (Asaropoda)

Morphological examination of type specimens in Australia and abroad and specimens from all major Australian museum collections, as well as newly collected material during Bush Blitz surveys and other collecting trips by the authors, revealed 21 species of which ten were recognised as new, while 15 names needed to be synonymised. Some of the male types of species described by Rayment (1931, 1951) lacked terminal metasomal segments, but this did not present a problem as a diagnostic character on S4 was still present. Association of the sexes was done either by identifying series of males and females collected from a single location and date, or by using DNA analyses. In contrast to species in *Zonamegilla*, *Asaropoda* species have numerous diagnostic characters, especially in males, which makes recognition of the species straightforward. However, the available DNA data also assisted with resolving the taxonomy.

### Diagnostic characters

The main diagnostic characters for *Asaropoda* in males are the shape and colour of a patch of setae on mid posterior margin of S4 and occasionally S3; the shape, width and depth of the emargination of S5 and S6; as well as genitalia characters, particularly the size and shape of the outer and inner gonostylus. For females, diagnostic characters include hair colour of tibial scopa, presence or absence of supra- and/or paraclypeal marks, presence or absence of dark hairs anteriorly on T2. In addition, the shape of a raised area on S6, which sometimes is drawn out in an acute spine (*A. houstoni*), a blunt process (*A. epaphrodita*), a pygidium like structure (*A. crenata*), a parabolic ridge (*A. albiclypeata*), or just an undefined rough area (*A. calva*) is a helpful diagnostic character. For males single diagnostic characters are often sufficient, however for females a combination of characters, particularly the pubescence colour and face marks, may be needed for identification of the species.

### Phylogenetic analyses

**Figure 1.**
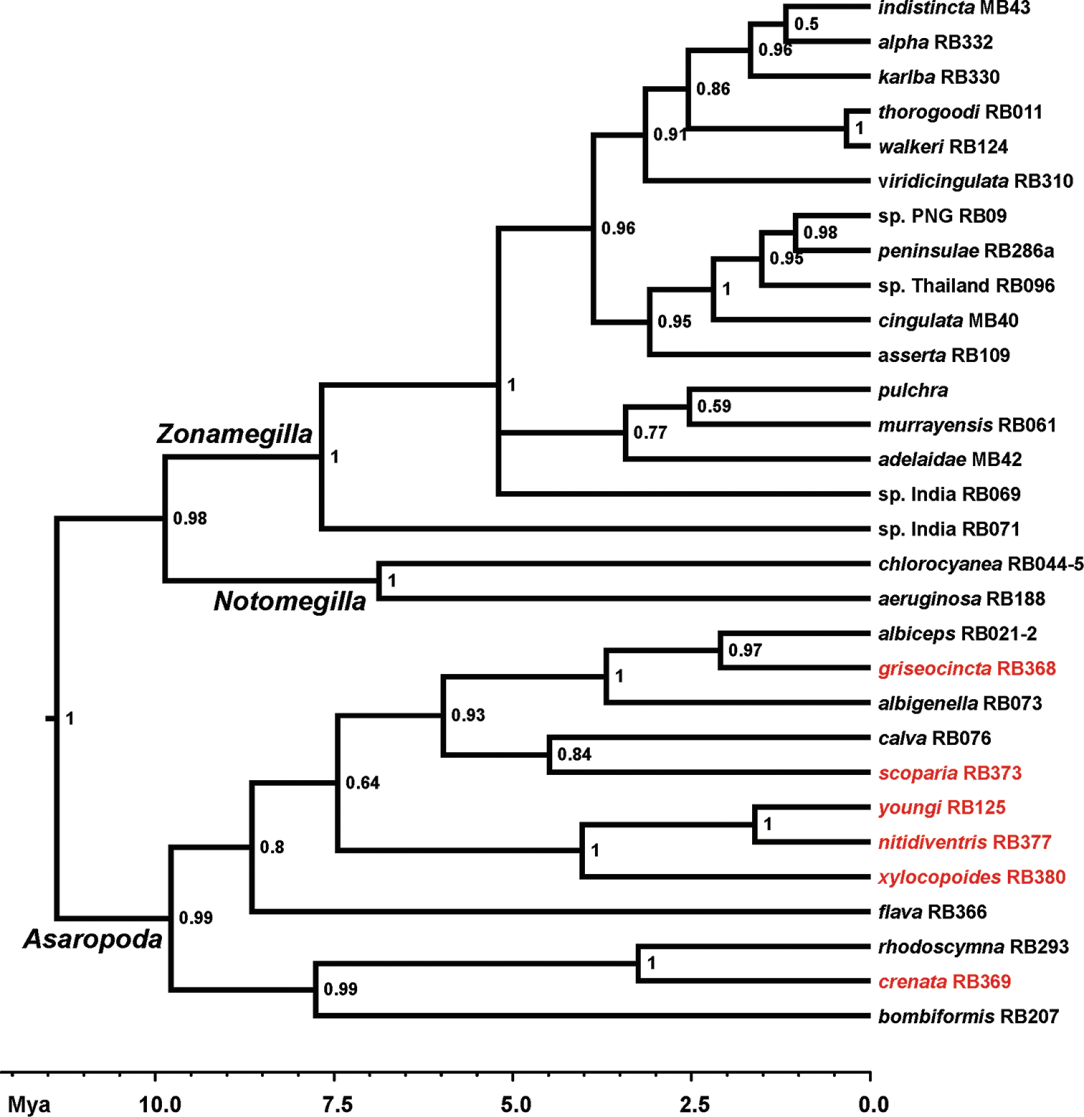
Bayesian molecular phylogenetic consensus tree of CO1 sequences. Node support values (posterior probabilities) are shown near the nodes. The terminals are labelled with species names (new species in red) and RB-numbers, which refer to Table S1.

DNA data were generated for 12 species, including six of the new species. These species were analysed together with data from other Australian *Amegilla* subgenera and species (Leijs et al. 2017) in order to examine support for the subgeneric division of the Australian species. The analysis showed strong support (pp > 0.98) for the subgeneric division as proposed by Brooks, 1988 with *Asaropoda* as sister group to *Zonamegilla* and *Notomegilla* (Figure 1). Subgeneric divergences were estimated as early as 9.8 Mya (5.3 – 15.8 95 % HDP) for *Asaropoda* and 9.8 Mya (5.3 – 15.7 95 % HDP) for the split between *Zonamegilla* and *Notomegilla*. Although the currently available DNA data cover only approximately half of the *Asaropoda* species, the phylogenetic tree shows a number of strongly supported (pp > 0.99) species groups, and this is supported by morphological traits such as general appearance, male sterna and genitalia.

The good match between the molecular and morphological data types allows the allocation of species for which no DNA data are as yet available into species groups. The differences in morphology between these groups, which are discussed below, are more pronounced than those between *Zonamegilla* and *Notomegilla*, but we consider it unnecessary at this stage to raise these groups to subgenus level, particularly as DNA data are lacking for circa half the taxa. The groups are:

The ***bombiformis*-group** containing *A. bombiformis*, *A. crenata* and *A. rhodoscymna*, these species all have reddish hairs and male genitalia have short gonostyli;

The ***houstoni*-group**, consisting of *A. epaphrodita*, *A. houstoni, A. xylocopoides* have black integument and orange or yellow mesosomal pubescence and predominantly black metasomal pubescence;

The ***youngi*-group**, consisting of *A. nitidiventris* and *A. youngi*; are superficially similar to A. (Zonamegilla) because they have posterior hairbands on the metasomal terga, the males of the last two groups have gonocoxa with long outer and inner gonostyli;

The ***albiceps*-group**, containing *A. albiceps*, *A. griseocincta*, and *A. albigenella*, of which the males are characterised by a relatively large triangular shaped patch of bristles apicomedially on S4 and genitalia with long outer gonostyli, but inner gonostyli are much reduced or lacking.

The three remaining species in the phylogenetic tree may contain the following lineages:

The ***flava*-lineage**, containing *A. flava* and most likely *A. preissi*, *A. albiclypeata* and *A. batleyi*, characterised by an elongated shaped patch of bristles apicomedially on S4 and male genitalia with stocky outer gonostyli, and short inner gonostyli;

The ***calva*-lineage**, containing *A. calva* and most likely *A. aurantia* and *incognita*, based on the relatively small and round shaped patch of bristles apicomedially on S4 and male genitalia with slender outer gonostyli, and short inner gonostyli; and

The ***scoparia*-lineage** that may contain *A. scoparia* and *A. frogatti* based on similar club shaped gonostyli, however sternal brushes do not match.

It is difficult to place *A. dawsoni* into the phylogeny without molecular data due to its aberrant morphology and body size.

**Figure 2.**
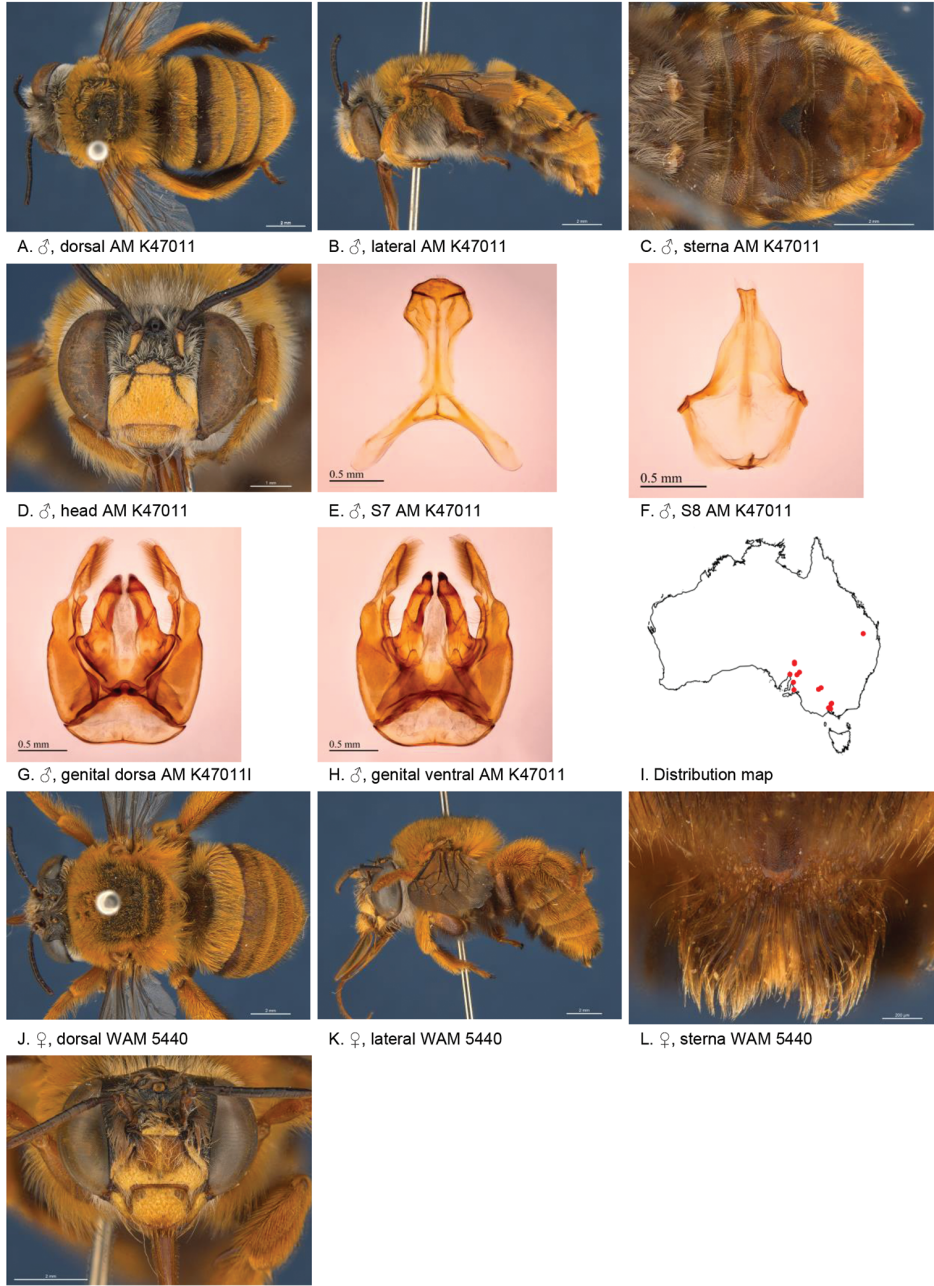
Amegilla (Asaropoda) albiceps (Rayment, 1951).

Considering the good match between phylogenetic species groups and their morphology, which mainly are male sexual characters, it is interesting to note that there are large dissimilarities in general appearance, mostly in pubescence colouration, between closely related species and this is especially the case when closely related species have overlapping or nearly overlapping distributions. Compare for example *A. albiceps* (Fig. 2) and *A. griseocincta* (Fig. 14). In sister species with non-overlapping distributions these differences are usually less pronounced, for example *A. youngi* (Fig. 22) - *A. nitidiventris* (Figure 17) and *A. rhodoscymna* (Fig. 19) - *A. crenata* (Fig. 9). With the exception of *dawsoni*, and apart from differences in face marks between the sexes, there are no clear sexual dimorphisms in general appearance in *Asarapoda* species. We hypothesise that dissimilarities in general appearance of closely related sympatric species were enhanced by sexual selection on mate recognition cues. Selection on general appearance may not have occurred in sister species pairs that may have speciated allopatrically.

### Distribution patterns

Brooks (1988: 515) suggested that *Asaropoda* was absent from the dry interior of Australia, although he noted that this may have been caused by a lack of sufficient sampling. However, this ‘lack of sampling’ may refer to insufficient sampling of Australian museum collections, which, at the time, contained more than 120 specimens of four species collected from the arid zone. In fact, *Asaropoda* species are widely distributed across Australia, New Guinea and the Bismarck Archipelago, but, as of yet, have not been found in Tasmania.

General distribution patterns of individual species of *Asaropoda* are similar to those found for species of the other subgenera of *Amegilla*. Roughly these patterns are east and west coastal, northern tropical, southern temperate and arid inland. Species with east-coast tropical and subtropical distributions are *bombiformis* and *A. rhodoscymna*, with a central arid distribution are *A. calva*, *A. scoparia*, and *A. albigenella*. Further, species with central-west, WA, distributions are *A. dawsoni* and *aurantia*, with a south-west temperate, WA, distribution is *preissi* and southern temperate distributions are *A. flava* and *A. albiceps*.

Often, closely related species do not overlap in distribution. For example, there are three cases of sister species pairs that are distributed east and west of the Gulf of Carpentaria in Northern Australia: *A. rhodoscymna* – *A. crenata*, *A. nitidiventris* – *A. youngi*, and *A. xylocopoides* – *A. epaphrodita*. The area south of the Gulf of Carpentaria has been described as a break in distribution for numerous taxa (Joseph and Omland 2009 and references therein). Similar disjunct distributions have been found between the sister species Amegilla (Zonamegilla) walkeri and A. (Z.) thorogoodi (Leijs et al. 2017). The estimated divergence times between the three *Asaropoda* sister species pairs (Figure 1: circa 1.6-3,3 Mya) correspond well with the Pleistocene age of the barrier as inferred for several other taxa (grass finches: Jennings and Edwards 2005, fairy wrens: Lee and Edwards 2008, rodents: Breed and Ford 2007, birds: Joseph and Omland 2009, blowflies: Wallman et al. 2005, and grasshoppers: Carsten and Knowles 2007), but seem slightly older than for the *Zonamegilla* species pairs (Figure 1: circa 0.5 Mya).

### Phenology

While for several species an accurate understanding of the phenology is hampered by small sample sizes, some general patterns can be deduced. As in other *Amegilla* (Leijs et al. 2017), the species’ activity patterns seem to be determined by the climatic variation in the flowering time of food plants. Species of the *bombiformis* group, which are known from the east coast and the northern tropics are found almost year-round, although there may be latitudinal and temporal variation in activity. Activity of *A. bombiformis* peaks at Jan-Mar. Species from the *houstoni* group, which are all distributed in the northern tropics seem to be active during the tropical wet season, Jan-Mar. Species with an inland distribution (*A. scoparia*, *A. calva*) are active during the cooler months, May-Nov. Southern temperate species (*A. albiceps*, *A. flava*, *A. preissi*) are found during Oct-Mar, with exception of *A. preissi* which was only collected during Feb-Mar. West coastal and inland species (*A. albiclypeata*, *A. aurantia*, *A. incognita*, *A. dawsoni*) were mainly found during Jul-Oct.

### Floral Records

Amegilla (Asaropoda) were collected on 26 genera of plants belonging to 20 families (248 records). Eighty-five percent of the records involve only five plant families: Scrophulariaceae (46.8%) represented by *Eremophila* species, Myrtaceae (17.7%) represented by *Eucalyptus*, *Corymbia*, *Angophora*, *Calothmanus*, and *Calytrix*, Boraginaceae (7.3%) with *Trichodesma*, Loranthaceae (6.9%) with *Amyema*, and Fabaceae (6.5%) with *Acacia*, *Senna*, and *Petalostylis*.

From the following *Asaropoda* species with a multitude of flower records (> 10) it can be hypothesised that these are polylectic: *A. scoparia* (55 records) was encountered on eight plant families; *A. dawsoni* (66 records) on four different families; and *calva* (24 records) on five different plant families. *Amegilla albiceps* (14 records) is the only species with multiple locality and flower records that has been collected on a single plant species, *Amyema preissi* (Loranthaceae). This bee species is active during summer and autumn and it is possible that during that time *Amyema* is the only reliable flower resource, especially in the drier inland habitats.

It is remarkable that for *A. bombiformis* there are only two floral records out of the 491 specimen records, probably because the majority of these records are from a time when flower visits were not recorded. However, based on the many images of *bombiformis* on the internet it is clear that this common species visits a large variety of plant families and species, including weeds such as *Lantana* (Verbenaceae) and introduced species such as *Duranta* (Verbenaceae).

*Amegilla* species are capable of buzz-pollination, and hence can use pollen sources in the landscape that are inaccessible to the ubiquitous introduced honey bee *Apis mellifera*.

## Systematics

### Subgenus Asaropoda

Taxon classificationAnimaliaHymenopteraApidae

Cockerell, 1926

37DB8DEF-3065-56A5-A35F-AA6BDCFBB7C1

Asaropoda Cockerell, 1926: 216.

#### Type.

*Saropoda bombiformis* Smith, 1854 (original designation).

#### Diagnosis.

The diagnosis for this subgenus as given by Brooks (1988) was largely based on the type species of the subgenus, *A. bombiformis*, but is revised to include species that he did not examine at the time, as well as the new species discovered here. The inclusion of these species extends the morphological variety in this subgenus.

In the following text, the main subgeneric diagnostic characters are in bold: **body length 12–24 mm; forewing length 8–18 mm; hairs absent in third submarginal cell and second medial cell**; hairs absent in first medial cell with the exception of *A. albiclypeata* and *A. youngi* which have a few short hairs, and *A. nitidiventris* which has more than ten hairs; hairs absent in the second submarginal cell with the exception of *A. albiceps*, *A. aurantia*, *A. frogatti*, and *A. rhodoscymna* which have less than ten hairs, and *A. crenata*, *A. preissi*, and *A. albiclypeata* which have 10–20 hairs; the pubescence of the majority of the species is orange, brown or grey, occasionally with black hairs anteriorly on T2 or following segments; however, there are two species groups with an aberrant pattern colouration of pubescence: the *houstoni*-group (*A. houstoni*, *A. epaphrodita*, *A. xylocopoides*), which has an orange to yellow mesosomal pubescence, and predominantly black metasomal pubescence, and the *youngi*-group (*A. nitidiventris* and *A. youngi*), which has brown mesosomal and black metasomal pubescence, with white to orange hairbands on the posterior margins. Colour dimorphism between the sexes is weak, apart from in *A. dawsoni*, where females have white to grey, and males brown mesosomal pubescence; maxiliary palpus with five segments; **apical margins of male metasomal sterna modified, with emarginations that vary in size and depth and are diagnostic at the species level**; S4 (and occasionally S3) bent medially; **the shape and size of the thick brush of stiff setae on S4 of males is diagnostic at the species level**; posterior margins of S5 and S6 emarginate medially of which its width, depth, size and shape is diagnostic at the species level; S6 medially with one or two patches of hair; S7 usually with slender neck, with the exception of *A. scoparia* and *A. frogatti* where the neck is robust; S7 apical margin of head among species variable in shape; S8 apically narrowed and usually emarginate; apex of gonocoxite of male bearing one or two gonostyli of which number, size and shape are diagnostic at the species level; gonostyli with setae of variable length and densities; penis valves laterally rounded, drawn into rounded lobes, or with angular lobes; **S6 of females medially with raised area which varies** from inconspicuous roughened, broadly parabolic with well-defined rim, slender spine, to almost a pygidium like structure.

We now recognise 21 species in Amegilla (Asaropoda).

##### Identification key to the Australian subgenera of *Amegilla*

**Table:** 

1	Forewing: hairs absent in 3^rd^ submarginal cell and 2^nd^ medial cell, usually also in 2^nd^ submarginal cell and 1^st^ medial cell; S4 in males with a rounded, triangular or elongated brush of dense usually forward-directed bristles, S6 of females medially with raised area which varies among species from inconspicuously roughened, broadly parabolic with well-defined rim and slender spine, to almost a pygidium like structure	**subgenus Asaropoda**
–	Forewing: hairs present in in 3^rd^ submarginal cell, 2^nd^ medial cell and most other cells; S4 in males and S6 in females not modified as described above	**2**
2	Female: Integument of paraclypeal areas black; fore and mid femora and tibiae with iridescent blue-green hairs, male: S6 gently convex; apex S7 broadly triangular	**subgenus Notomegilla**
–	Female: Integument of paraclypeal areas partly yellow, white or ivory; hair on fore and mid legs never iridescent, male: S6 with broad depressions either side of midline; apex S7 ovate	**subgenus Zonamegilla**

##### Identification key to the 21 species of Amegilla (Asaropoda)

**Table:** 

1	Female: Body size large (> 19 mm), pubescence on head and mesosoma white, metasomal integument predominantly dark red (Fig. 10J, K). Male: S4 apicomedially with two small adjacent patches of anteriorly directed, black bristles (Fig. 10C), S5 with parabolically shaped emargination (Fig. 10C)	***A. dawsoni***
–	Not with above combination of characters (20 spp.)	**2**
2	Metasomal pubescence predominantly black, some species with light coloured posterior bands of white pubescence on terminal terga (5 spp.)	**3**
–	Metasomal pubescence predominantly orange, brown or grey, with or without an anterior band of black pubescence on T2 (15 spp.)	**7**
3	Light coloured posterior bands present on all terga (Figs 15, 17, 22) (3 spp.)	**4**
–	Light coloured posterior bands lacking, or only on last 1-3 terminal terga (Figs 21, 11) (2 spp.)	**6**
4	Female: T1 with thin, faint white posterior band, T2-4 with well-developed white posterior hair bands (Fig. 15). Male unknown	***A. houstoni***
–	All terga with well-developed posterior hair bands. Hair bands on T1-2 orange (2 spp.)	**5**
5	Female: clypeus ivory with two large black marks, which are sometimes combined at the top, scape black. Male: S4 with two adjacent small patches of downward-directed bristles on apicomedial margin and two large comma-shaped patches of bristles on S5 (Fig. 17C)	***A. nitidiventris* sp. nov**.
–	Female: clypeus with two small brown marks, scape with pale yellow mark (Fig. 22C). Male unknown	***A. youngi* sp. nov.**
6	Metasomal pubescence entirely black (Fig. 21B, K)	***A. xylocopoides* sp. nov.**
–	T4-5 (female), T5-6 (male) with predominantly white hairs (Fig. 11B, K)	***A. epaphrodita***
7	Male	**8**
–	Female	**21**
8	Both S3 and S4 medially with round patch of bristles, the patch on S3 smaller than on S4 (Figs 5C, 9C, 19C) (3 spp.)	**9**
–	Only S4 with median patch of bristles of varying size and shape (Figs 7C, 8C, 20C) (12 spp.)	**11**
9	S2-4 without apicomedial emarginations; S5 apicomedial area deeply emarginated, emargination deeper than wide and almost parallel to body axis (Figs 9C, 19C) (2 spp.)	**10**
–	S2-4 with distinct apicomedial emarginations (Fig. 5C); apicomedial emargination on S5 wider than deep, not parallel to body axis	***A. aurantia* sp. nov.**
10	Apicomedial emargination on S5 parallel-sided, slightly deeper than wide (Fig. 19C)	***A. rhodoscymna***
–	Apicomedial emargination on S5 more than twice as deep as wide (Fig. 9C)	***A. crenata* sp. nov.**
11	S4 shape of patch of bristles round (Figs 8C, 13C, 16C) (3 spp.)	**12**
–	S4 shape of patch of bristles triangular or bell-shaped (Figs 2C, 4C, 7C, 14C) (4 spp.)	**14**
–	S4 shape of patch of bristles elongated (Figs 3C, 18C, 20C) (5 spp.)	**17**
12	S4 patch of bristles relatively large, circa 1/7 of width of sternum (Figs 13C, 16C), pubescence around apicomedial area of S5 strongly branched (2 spp.)	**13**
–	S4 patch of bristles small, less than 1/10 of width of sternum (Fig. 8C), pubescence around apicomedial area of S5 almost simple, only occasionally with side branches	***A. calva***
13	Face marks pale yellow, scape brown (Fig. 16D), inner gonostylus very small (Fig. 16G, H)	***A. incognita* sp. nov.**
–	Face marks ivory, underside of scape with ivory mark (Fig. 13D), inner gonostylus well developed, almost as long as outer gonostylus, club-shaped (Fig. 13G, H) .	***A. frogatti***
14	S4 patch of bristles small, circa 1/5 of width of sternum (Fig. 7C), S5 apicomedial area with large triangular emargination (Fig. 7C), the area with sparse to openly placed branched hairs, lateral corners of S5 rounded	***A. bombiformis***
–	S4 patch of bristles larger, S5 apicomedial emargination shallow (Figs 2C, 4C, 14C), densely covered in branched hairs, lateral corners of S5 with pointed lobe (3 spp.)	**15**
15	S4 patch of bristles large, circa 1/3 of width of sternum, bristles brown (Fig. 4C)	***A. albigenella***
–	S4 patch of bristles less than 1/3 of width of sternum, bristles black (2 spp.)	**16**
16	S4 patch of bristles triangular (Fig. 2C), legs on outer surface with orange pubescence.	***A. albiceps***
–	S4 patch of bristles bell-shaped (Fig. 14C), legs on outer surface with white pubescence	***A. griseocincta* sp. nov.**
17	S4 patch of black bristles broad and circa 1/2-2/3 of sternum width (Fig. 20C), S6 apicomedial area with conspicuous fimbria of black and orange fringed hairs, T2 anteriorly with area of black hairs as wide as marginal zone	***A. scoparia* sp. nov.**
–	S4 patch of pale bristles narrow, circa 1/3 of width of sternum, S6 apicomedial area without conspicuous fimbria of black and orange-fringed hairs, T2 anteriorly with area of black hairs much narrower than marginal zone, or without black hairs (4 spp.)	**18**
18	Face marks ivory/white, elongated patch of bristles on S4 black (Fig. 3C)	***A. albiclypeata* sp. nov.**
–	Face marks pale to bright yellow, patch of bristles on S4 dark or light brown (3 spp.)	**19**
19	T2 anteriorly without dark hairs, S5 apicomedial emargination circa 6 × as wide as deep, (Fig. 6C)	***A. batleyi* sp. nov.**
–	T2 anteriorly with entire narrow band of dark hairs, S5 apicomedial emargination less than 4 × as wide as deep	**20**
20	S6 marginal zone smooth without punctures or hairs, some short hairs medially (Fig. 18C)	***A. preissi***
–	S6 marginal area, smooth with open punctures and hairs, medially with patch of stiff dark hairs (Fig. 12C)	***A. flava***
21	Hind leg: tibial scopa on outer surface with white or grey-white hairs (5 spp.)	**22**
–	Hind leg: tibial scopa on outer surface with pale yellow, ochre or orange hairs (8 spp.)	**26**
22	Paraclypeal area with pale yellow mark (3 spp.)	**23**
–	Paraclypeal area black (3 spp.)	**25**
23	Paraclypeal marks small (Fig. 6M), anterior part of T2 without black hairs	***A. batleyi* sp. nov.**
–	Paraclypeal marks large (Figs 13M, 14M), anterior part of T2 with distinct band of black contiguous hairs	**24**
24	Scutum and T1 pubescence light brown, T2 and T3 anteriorly with black hairs	***A. griseocincta* sp. nov.**
–	Scutum and T1 pubescence appears grey, hairs light ochre intermixed with longer black hairs, only T2 with black hairs anteriorly	***A. frogatti***
25	Supraclypeal area black, clypeus with yellow inverted T shaped mark (Fig. 18M), anterior part of T2 with narrow band of black hair, terga with greyish pubescence, S6 with smooth and clearly defined parabolic expression (Fig. 18L)	***A. preissi***
–	Supraclypeal area with yellow or pale yellow mark, clypeus with two vertical brown marks (Fig. 8M), T2 without black hairs anteriorly, surface of S6 roughend, expression on S6 not clearly defined (Fig. 8L)	***A. calva***
26	Paraclypeal area with light coloured mark (3 spp.)	**27**
–	Paraclypeal area without light coloured mark (6 spp.)	**29**
27	T2 anteriorly with narrow band of black hairs across whole width, sterna with dark hairs	***A. albiceps***
–	Dark hairs on T2 restricted to antero-lateral corners or lacking, sterna on disk with orange hairs (2 spp.)	**28**
28	Integument of T3 and following segments anteriorly dark brown, clypeus with two well defined vertical dark orange marks (Fig. 4M), S6 with small angular raised median ridge (Fig. 4L).	***A. albigenella***
–	Integument of T3 and following segments anteriorly orange, marks on clypeus undefined and small (Fig. 5M), S6 with narrow raised area and short transverse posterior ridge (Fig. 5L)	***A. aurantia* sp. nov.**
29	T2 anteriorly with black hairs (2 spp.)	**30**
–	Pubescence on T2 uniformly coloured (4 spp.)	**31**
30	Scape orange, T2 anterior with wide area of black hairs, clypeus marks undefined (Fig. 7M), the orange pubescence on terga has an iridescent shine	***A. bombiformis***
–	Scape black, apically brown, T2 anteriorly with narrow area of erect black hairs, clypeus with brown horseshoe-like mark (Fig. 20M), sometimes just with two dark patches, pubescence on terga not with iridescent shine	***A. scoparia* sp. nov.**
31	Clypeus colouration yellow, orange pubescence on terga with iridescent shine, S6 projection broadly parabolic, well defined (Figs 9L, 19L) (2 spp.).	**32**
–	Clypeus colouration pale yellow or ivory, pubescence on terga without iridescent shine, S6 projection rounded, not well defined (Figs 3L, 12L) (2 spp.)	**33**
32	Clypeus marked with inverted yellow T (Fig. 19M), scape and antennae brown	***A. rhodoscymna***
–	Clypeus yellow, with two faint brown patches (Fig. 9M), scape orange-brown, flagellum brown below, black above	***A. crenata* n.sp.**
33	Clypeus with pale yellow inverted T-shape, supraclypeal area black (Fig. 12M). Hind leg: tibial scopa on outer surface with pale yellow or ochre coloured hairs (Fig. 12K). [Note: this couplet may also lead to *A.* (*A*) *scymna*, but shape of pale yellow clypeus colouration similar to Fig. 3M]	***A. flava***
–	Clypeus colouration ivory, supraclypeal area black with narrow ivory line at base (Fig. 3M). Hind leg: tibial scopa on outer surface with orange coloured hairs (Fig. 3K)	***A. albiclypeata* sp. nov.**

### Amegilla (Asaropoda) albiceps

Taxon classificationAnimaliaHymenopteraApidae

(Rayment, 1951)

45B6BE68-EB66-56DF-94D2-CF3AEBAA7A54

Figure 2A–M

Asaropoda albiceps Rayment, 1951: 71.
Asaropoda dentiventris Rayment, 1951: 73, **syn. nov.**
Asaropoda meltonensis Rayment, 1951: 74, **syn. nov.**
Asaropoda victoriensis Rayment, 1951: 77, **syn. nov.**

#### Specimens examined

(22 males, 5 females).

#### Types.

Holotype of *A. albiceps*: male, Studley Park, N40, VIC, MV T-11866.

Holotype of *A. dentiventris*: male, Broadmeadows, N50, VIC, donated by FP Spry, Oct. 1922, MV T-20875, the specimen is lacking terga and sterna from S5 onwards.

Holotype of *A. meltonensis*: female, Melton, VIC, FE Wilson 21 Jun. 1920, on flowers of *Loranthus*, MV T-11867.

Holotype of *A. victoriensis*: male, Broadmeadows, N32, VIC, donated by FP Spry, 5 Oct. 1922, MV T-11868.

#### Decision for synonymy.

The emargination and hair patches of the sterna of the males of *A. dentiventris* and *A. victoriensis* are not different from those of *A. albiceps*. The female diagnostic characters of *A. albiceps*, including the colour of the pubescence on the tibial scopa, pale marks on paraclypeal areas and presence of black hairs on the anterior margin of T2, are also present on the holotype of *A. meltonensis*.

#### Diagnosis.

Male with triangular patch of black bristles on S4 and shallow emarginated apicomedial area of S5. Female with narrow yellow paraclypeal mark along clypeus, supraclypeal mark present, tibial scopa orange, T2 with black hairs anteriorly, process on S6 irregularly striate, not well defined posteriorly (Fig. 2L).

#### Redescription.

Male (AM K470111): Body length 14 mm, forewing length 9.7 mm, head width 5.0 mm.

***Structure***: Inner orbits of eyes diverging above; head wider than long; clypeal protuberance in profile 0.53 × eye width; mandible with subapical tooth; F1 equal to combined length of next 2.5 flagellomeres; F1 0.96 × as long as scape; F2 0.57 × as long as F3; F3-F10 1.25 × as long as wide; last flagellomere 0.77 × as long as F1; marginal cell length 0.85 distance from apex of cell to wing tip; cu-v of hind wing 0.87 × length of second abscissa of M+Cu; S5 with apicomedial emargination round, 2.9 × as wide as deep; posterior lateral corners drawn into short spine; S6 with apicomedial emargination more than six times as wide as deep.

*Genitalia*: penis valves with well extended shoulders; volsella slightly elongated with circa 12 setae (Fig. 2H); gonocoxa laterally with a few short setae; apex of gonocoxa with rounded dorsal and ventral lobes; outer gonostylus robust, with strong setae on inner surface; inner gonostylus very small, reduced to transparent membrane with a few setae (Fig. 2G); S7 (Fig. 2E); S8 narrowed towards apex, slightly emarginate (Fig. 2F).

***Pubescence***: Head white, apart from some dark hairs on vertex, lower ocellocular area and upper supraclypeal area; scutum, scutellum and metanotum with pale orange hairs intermixed with dark hairs; mesosoma laterally and ventrally with pale orange hairs under the wing base, remaining hairs white; fore leg with white hairs on femur and pale orange hairs on tibia and tarsus; mid and hind legs orange on outer surfaces, brown-black on inner surfaces; metasomal terga with pale orange hairs; T2 anteriorly with wide band of black hairs; S1-S5 with fringes of pale brown hairs near premarginal line and longer brown hairs on disks; S4 apicomedial area with triangular patch of black anteromedial directed bristles, laterally with plumes of long grey hairs; S5 with area preceding the emargination covered in long branched posteromedial directed hairs; S5 laterally with plumes of long orange hairs; S6 with small patch of orange hairs posterior to rounded ridge, longer hairs in the emargination.

***Colouration***: Integument of head, mesosoma and anterior metasomal terga black; posterior margins of metasomal terga translucent orange; sterna orange-brown; legs: coxa, trochanter and femur and inner surface of tibia brown, remaining parts orange; scape pale yellow below; flagellum dark brown; labrum yellow with vague brown dots near dorsolateral corners; clypeus yellow, supraclypeal area yellow; paraclypeal area yellow; mandible yellow at base, black-brown at tip; proboscis orange.

**Female** (WAM 5440): Body length 14 mm, forewing length 10.2 mm, head width 5.5 mm.

***Structure***: Inner orbits of eyes diverging above; head wider than long; clypeal protuberance in profile 0.49 × eye width; mandible with subapical tooth; F1 equal to combined length of next 2.8 flagellomeres; F1 as long as scape; F2 0.83 × as long as F3; F3-F10 slightly longer than wide; last flagellomere 0.54 × as long as F1; marginal cell length 0.83 distance from apex of cell to wing tip; cu-v of hind wing 0.89 × length of second abscissa of M+Cu; S6 with small rounded raised transverse median ridge (Fig. 2L).

***Pubescence***: Head white, with some dark hairs on vertex, lower ocellocular area and upper supraclypeal area; scutum, scutellum and metanotum with pale orange hairs intermixed with dark hairs; mesosoma laterally and ventrally with pale orange hairs under the wing base, paler below, remaining ventral hairs dark brown; fore leg with pale orange hairs; mid and hind legs orange on outer surfaces, brown-black on inner surfaces; metasomal terga with pale orange hairs intermixed with scattered erect black hairs; T2 anteriorly with narrow band of black hairs; T5 with brown prepygidial fimbria; T6 with strong orange-brown hairs flanking the pygidial plate; S1-S5 with brown-black hairs.

***Colouration***: Integument of head black, apart from: scape orange-brown, flagellum brown below, black above; labrum pale yellow with translucent dots near dorsolateral corners; clypeus pale yellow with large brown dorsolateral patches resulting in narrow pale yellow midline; supraclypeal area pale yellow; paraclypeal area pale yellow; mandible yellow at base, black-brown at tip; proboscis orange; mesosoma and anterior metasomal terga black; posterior margins of metasomal terga translucent orange; sterna orange-brown; legs: coxa, trochanter and femur and inner surface of tibia brown, remaining parts orange.

**Table:** **Phenology.**

**Month**:	**Jan**	**Feb**	**Mar**	**Apr**	**May**	**Jun**	**Jul**	**Aug**	**Sep**	**Oct**	**Nov**	**Dec**
No. of records:	4	12	2	0	0	1	0	0	0	2	1	0

#### Flower records.

*Loranthus preissi*, *Amyema* sp. (Loranthaceae), *Eremophila* sp. (Scrophulariaceae).

#### Distribution.

Figure 2I.

### Amegilla (Asaropoda) albiclypeata

Taxon classificationAnimaliaHymenopteraApidae

Leijs, sp. nov.

55168373-3DE6-5844-B4DB-BC7B702FAC76

http://zoobank.org/654C6060-89FF-48B4-86B9-E0A49A7A7FAD

Figure 3A–M

#### Specimens examined

(7 males, 2 females).

#### Types:

Holotype, male, Useless loop road, 8-9 km from Derham Road, WA (26.5166S; 114.0056E), 21 Nov. 1998, TF Houston, on *Calothamnus formosus formosus*, WAM 21905;

Allotype, female, WAM 21904, same locality data as holotype;

Paratype, male, WAM 21906, same locality data as holotype.

#### Diagnosis.

**Figure 3.**
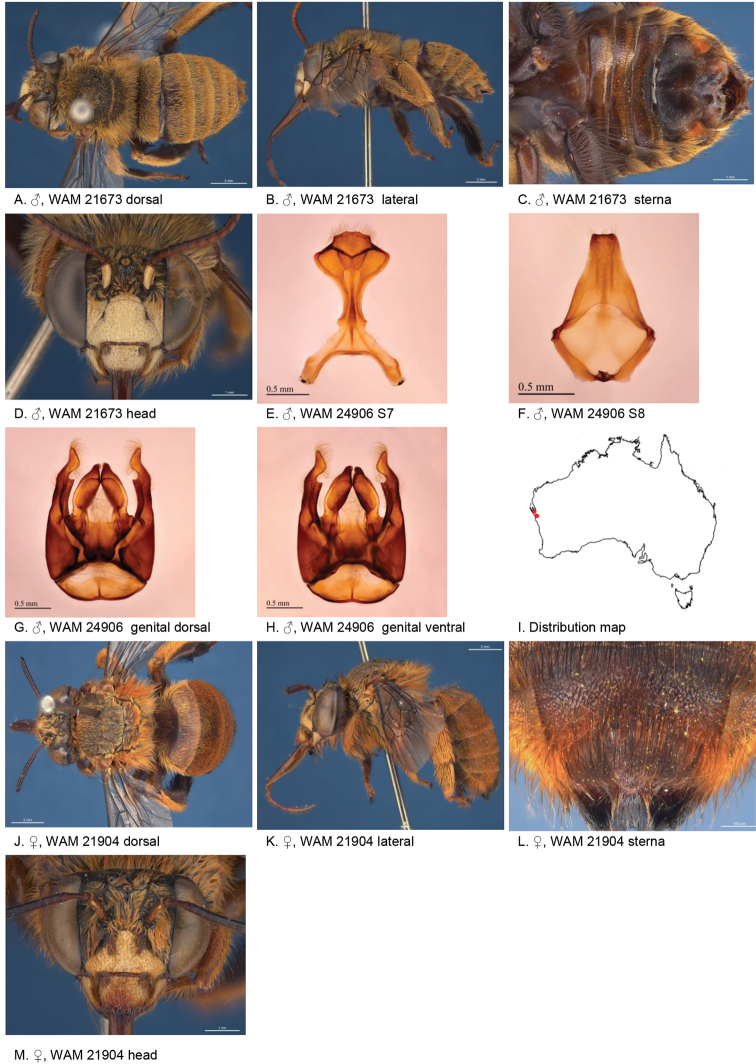
Amegilla (Asaropoda) albiclypeata Leijs, sp. nov.

Face marks of both sexes ivory. Male S4 with rectangular shaped patch of black bristles, and broadly emarginated posterior rim of S5. Female paraclypeal and supraclypeal marks absent, tibial scopa outer surface orange, T2 with a few black hairs in anterolateral corners, process on S6 striate, little defined posteriorly (Fig. 3L).

#### Description.

Male holotype (WAM21905): Body length 14 mm, forewing length 9.2 mm, head width 5.0 mm.

***Structure***: Inner orbits of eyes diverging above; head wider than long; clypeal protuberance in profile 0.32 × eye width; mandible with subapical tooth; F1 equal to combined length of next 2 flagellomeres; F1 0.75 × as long as scape; F2 0.53 × as long as F3; F3-F10 1.2 × as long as wide; last flagellomere 0.83 × as long as F1; marginal cell length 0.76 × distance from apex of cell to wing tip; cu-v of hind wing 1.3 × length of second abscissa of M+Cu; S5 with apicomedial emargination triangular, 2.9 × as wide as deep; S6 with apicomedial emargination three times as wide as deep.

*Genitalia*: penis valves with well extended shoulders; volsella elongated with circa 20 setae of variable length (Fig. 3H); gonocoxa laterally with a few short setae; apex of gonocoxa with acute lobe ventrally; outer gonostylus truncated, short with robust setae on inner surface; inner gonostylus very small, rounded, with setae; S7 (Fig. 3E); S8 apex straight, a few short setae around the mid line (Fig. 3F).

***Pubescence***: Head pale yellow, darker on frons and vertex, some black hairs around ocelli and vertex; scutum, scutellum and metanotum with pale orange intermixed with black hairs; mesosoma laterally and ventrally pale orange; fore leg pale orange, mid and hind legs: coxa, trochanter, and inner surface of femur, tibia, and metatarsus with dark brown pubescence, outer surface pale orange; metasomal terga with pale orange hairs, T2 anteriorly with narrow band of black hairs; T6 posterior margin bare; S1-S5 with sparse rows of dark hairs near premarginal line and pale hair bands on posterior margins laterally; S4 apicomedial area with elongated patch of black anteromedially-directed bristles, patch width circa 0.4 sternum width; S5 with area preceding the emargination covered in long branched posteromedial directed hairs; S5 laterally with plumes of long orange hairs; S6 with smooth and shiny posterior margin almost without hairs.

***Colouration***: Integument of head black, apart from: scape ivory below; flagellum brown; labrum ivory with small brown marks in dorsolateral corners; clypeus ivory; supraclypeal area with ivory triangle; paraclypeal area ivory; mandible ivory at base, black at tip; proboscis orange-brown; mesosoma and metasomal terga black; mid and hind coxa, trochanter and femur and inner surface of tibia; front legs, metasomal sterna and posterior margins of metasomal terga orange.

**Female** allotype (WAM21904): Body length 15 mm, forewing length 10.2 mm, head width 5.3 mm.

***Structure***: Inner orbits of eyes diverging above; head wider than long; clypeal protuberance in profile 0.73 × eye width; mandible with subapical tooth; F1 equal to combined length of next 3 flagellomeres; F1 0.86 × as long as scape; F2 0.75 × as long as F3; F3-F10 circa as long as wide; last flagellomere 0.66 × as long as F1; marginal cell length 0.65 distance from apex of cell to wing tip; cu-v of hind wing 0.93 × length of second abscissa of M+Cu; S6 with raised area and parabolic posterior ridge (Fig. 3L)

***Pubescence***: Head pale yellow, darker on frons and vertex, some black hairs around ocelli and vertex; scutum, scutellum and metanotum pale orange intermixed with black hairs; mesosoma laterally and ventrally pale orange; fore leg orange; mid and hind legs: coxa, trochanter and inner surface of femur, tibia and metatarsus with dark brown to black pubescence, outer surface orange; metasomal terga orange, T2 anteriorly with a few black hairs at anterolateral corners, T5 with black prepygidial fimbria, T6 with strong black hairs flanking the pygidial plate; S1-S5 with rows of black hairs near premarginal line; hairs on S4 and S5 much longer and denser than on previous sterna.

***Colouration***: Integument of head black, apart from: scape with ivory streak below; flagellum brown; labrum ivory with brown marks in dorsolateral corners; clypeus ivory with two small brown marks; supraclypeal area and paraclypeal area black; mandible ivory at base, black at tip; proboscis brown, mesosoma and metasomal terga black; mid and hind coxa, trochanter and femur and inner surface of tibia; metasomal sterna and posterior margins of metasomal terga orange.

**Table:** **Phenology.**

**Month**:	**Jan**	**Feb**	**Mar**	**Apr**	**May**	**Jun**	**Jul**	**Aug**	**Sep**	**Oct**	**Nov**	**Dec**
No. of records:	0	0	0	0	0	0	0	0	0	0	9	0

#### Flower records.

*Calothamnus formosus formosus*, *C. blepharospermus* (Myrtaceae).

#### Distribution.

Figure 3I.

#### Etymology.

The specific epithet refers to the white face marks of this species.

### Amegilla (Asaropoda) albigenella

Taxon classificationAnimaliaHymenopteraApidae

Michener, 1965

077252B9-6FD0-5D4A-AF11-87D8744148E7

Figure 4A–M

Amegilla (Asaropoda) albigenella Michener 1965: 217.
Asaropoda albigena Rayment, 1931: 182.

#### Specimens examined.

17 males, 12 females.

Holotype of *A. albigena*, male, Landor Stn, 29-882, WAM 23013.

#### Diagnosis.

**Figure 4.**
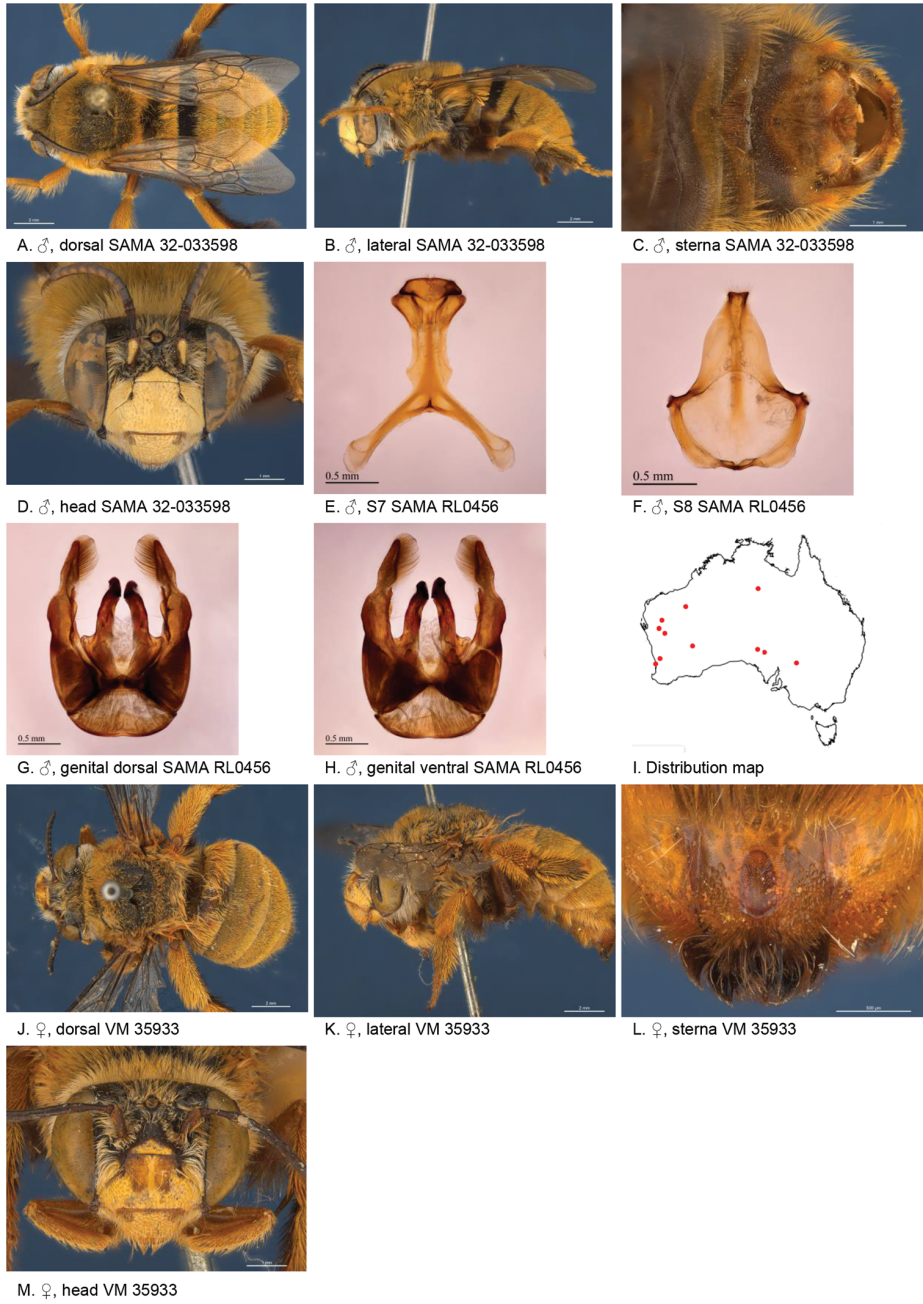
Amegilla (Asaropoda) albigenella Michener, 1965.

Male with almost triangular patch of light brown stiff bristles on S4 and emarginate apicomedial area of S5 surrounded by dense branched hairs, lateral corners of S5 protruding. Female paraclypeal and supraclypeal marks present, tibial scopa ochre, T2 with a few black hairs anteriorly, process on S6 large narrow, posteriorly with angular smooth ridge Fig. 4L).

#### Redescription.

Male (SAMA 32-033598, RL0456) Body length 14 mm, forewing length 10.1 mm, head width 4.7 mm.

***Structure***: Inner orbits of eyes diverging above; head wider than long; clypeal protuberance in profile 0.87 × eye width; mandible with small subapical tooth; F1 equal to combined length of next 2.3 flagellomeres; F1 0.87 × as long as scape; F2 0.6 × as long as F3; F3-F10 1.25 × as long as wide; last flagellomere 0.78 × as long as F1; marginal cell length 0.72 distance from apex of cell to wing tip; cu-v of hind wing longer than second abscissa of M+Cu; S5 with apicomedial emargination circa five times as wide as deep; posterior lateral corners drawn into distinct lobe, S6 with apicomedial emargination circa 3.5 × as wide as deep; emargination preceded by parabolic raised smooth ridge.

*Genitalia*: penis valves with well extended shoulders; volsella with circa ten long setae (Fig. 4H); gonocoxa laterally with setae lacking; apex of gonocoxa with broadly rounded dorsal lobe and small ventral lobe; outer gonostylus robust, with strong setae on inner surface; inner gonostylus very small, reduced to transparent membrane (Fig. 4G); S7 (Fig. 4E); S8 narrowed towards apex, slightly emarginate (Fig. 4F).

***Pubescence***: Head white, apart from pale yellow on vertex, a few dark hairs below ocelli, scutum, scutellum and metanotum with pale orange hairs intermixed with dark hairs, mesosoma laterally pale orange on dorsal half under the wing base, remaining hairs white, fore leg with pale yellow, mid femur mid and hind tibia and tarsus pale orange on outer surfaces, femur, tibia and tarsus on inner surfaces black-brown, metasomal terga with pale orange hairs, T2 anteriorly with wide band of black hairs; T3 with few black hairs in anterolateral corners, S1-S5 with pale yellow hairs, S4 apicomedial area with triangular patch of brown anteromedially-directed bristles, circa 1/3 the width of the sterna (Fig. 4C), S5 area preceding the emargination covered in long brown branched posteromedially directed hairs; S5 laterally with plumes of long orange hairs on posteriolateral lobes, S6 with patch of orange stiff hairs posterior to rounded ridge, longer branched hairs in the emargination.

***Colouration***: Integument of head black, apart from scape orange-brown, flagellum brown below, black above, labrum pale yellow with translucent dots near dorsolateral corners, clypeus pale yellow, supraclypeal area pale yellow, paraclypeal area pale yellow, mandible pale yellow at base, black at tip, proboscis orange-brown, mesosoma and anterior metasomal terga black; posterior margins of metasomal terga translucent orange; sterna orange-brown; legs: coxa, trochanter and femur and inner surface of tibia brown, remaining parts orange.

#### Female

redescription (VM 35933): Body length 14.5 mm, forewing length 10.6 mm, head width 5.2 mm.

***Structure***: Inner orbits of eyes diverging above; head wider than long; clypeal protuberance in profile 0.69 × eye width; mandible with subapical tooth; F1 equal to combined length of next 3 flagellomeres; F1 0.9 × as long as scape; F2 0.75 × as long as F3; F3-F10 slightly longer than wide; last flagellomere 0.6 × as long as F1; marginal cell length 0.76 × distance from apex of cell to wing tip; cu-v of hind wing as long as second abscissa of M+Cu; S6 with small angular raised median ridge (Fig. 4L).

***Pubescence***: Head white, apart from pale yellow on vertex, a few dark hairs below ocelli; scutum, scutellum and metanotum with pale orange hairs intermixed with dark hairs; mesosoma laterally and ventrally pale orange under the wing base, remaining hairs pale yellow to white; fore leg pale yellow; mid and hind tibia and tarsus pale orange on outer surfaces, femur, and tibia and tarsus on inner surfaces black-brown; metasomal terga with pale orange hairs; T2 anteriorly only with a few erect black hairs; T3 without black hairs in anterolateral corners; T5 with brown prepygidial fimbria, T6 with strong orange-brown hairs flanking the pygidial plate and much darker at the apex; S1-S5 with pale yellow hairs and few stiff erect dark hairs on disks of S1-S4.

***Colouration***: Integument of head black, apart from: scape orange-brown, flagellum brown below, black above; labrum pale yellow with translucent dots near dorsolateral corners; clypeus pale yellow with large brown dorso lateral patches resulting in pale yellow midline; supraclypeal area pale yellow; paraclypeal area pale yellow; mandible pale yellow at base, black at tip; proboscis orange-brown. Mesosoma and anterior metasomal terga dark brown; posterior margins of metasomal terga somewhat translucent, sterna orange-brown; legs: coxa, trochanter and femur and inner surface of tibia brown, remaining parts orange.

**Table:** **Phenology.**

**Month**:	**Jan**	**Feb**	**Mar**	**Apr**	**May**	**Jun**	**Jul**	**Aug**	**Sep**	**Oct**	**Nov**	**Dec**
No. of records:	1	0	1	2	15	0	0	0	1	7	0	1

#### Flower records.

*Eremophila glabra, Eremophila alternifolia, Eremophila longifolia* (Scrophulariaceae), *Eucalyptus* (Myrtaceae).

#### Distribution.

Figure 4I.

### Amegilla (Asaropoda) aurantia

Taxon classificationAnimaliaHymenopteraApidae

Leijs, sp. nov.

80CED679-978E-56FC-B283-827F2DA339DB

http://zoobank.org/CCA3E970-8F98-43EA-96CA-8FE2827632AC

Figure 5A–M

#### Specimens examined.

(6 males, 8 females).

#### Types.

Holotype, male, Mount Augustus, WA (24.3261S; 116.8400E), 03 Sept. 1980, CA Howard & TF Houston, WAM 5476, on *Pityrodia augustensis*;

Allotype, female, Yampire Gorge, WA (22.3738S; 118.4675E), 26 Aug. 1971, TF Houston, WAM 5457.

#### Diagnosis.

**Figure 5.**
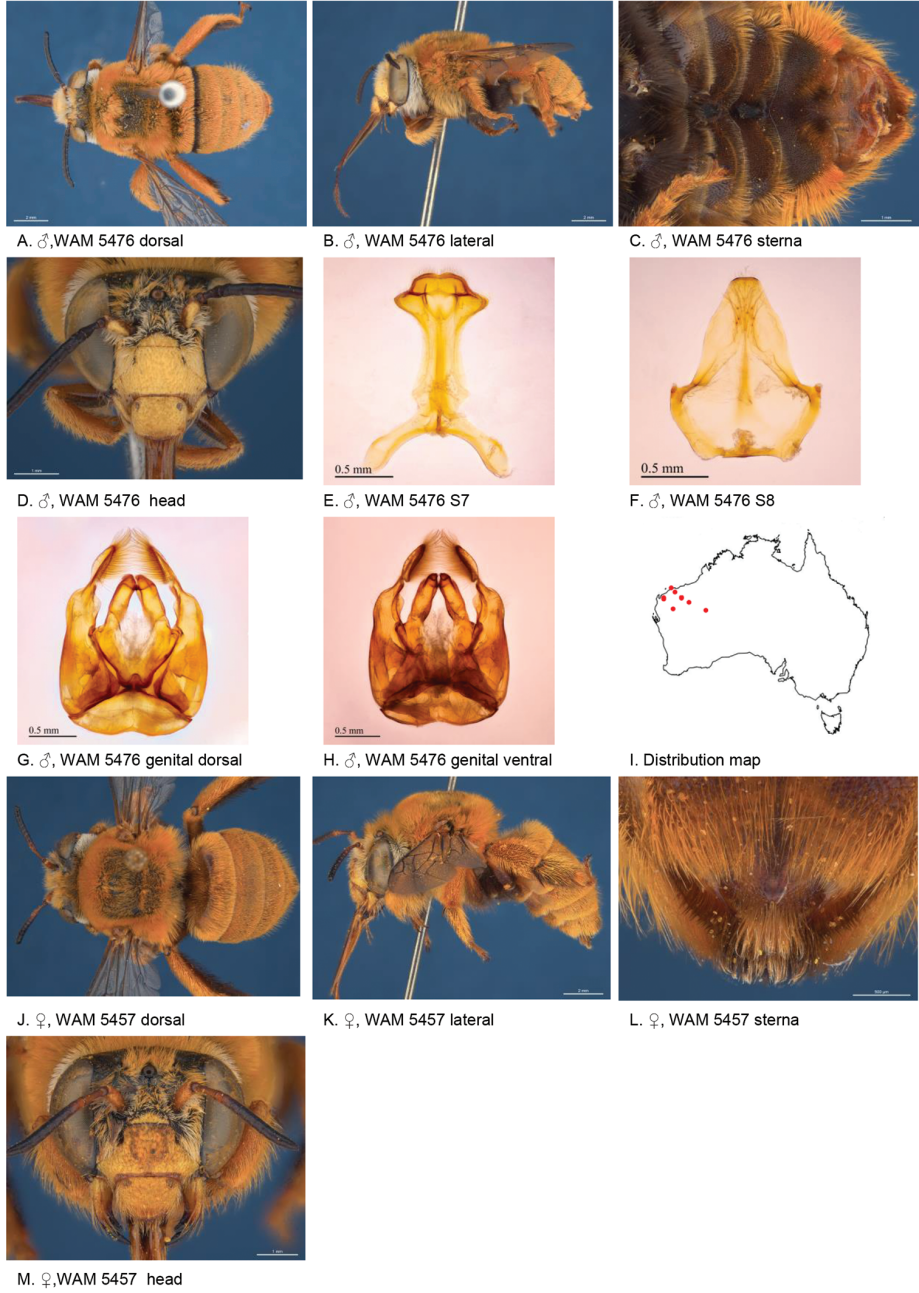
Amegilla (Asaropoda) aurantia Leijs, sp. nov.

Male S3 and S4 broadly emarginated each with round patch of black bristles. Female paraclypeal and supraclypeal area with pale yellow marks, tibial scopa orange, T2 with some black hairs in anterolateral corners, process on S6 irregularly striate, not well defined posteriorly (Fig. 5L)

#### Description.

Male holotype (WAM5476): Body length 13 mm, forewing length 9 mm, head width 4.4 mm.

***Structure***: Inner orbits of eyes diverging above and below; head wider than long; clypeal protuberance in profile 0.67 × eye width; mandible with subapical tooth; F1 equal to combined length of next 1.6 flagellomeres; F1 0.63 × as long as scape; F2 0.67 × as long as F3; F3-F10 1.3 × as long as wide; last flagellomere 1.1 × as long as F1; marginal cell length 0.69 × distance from apex of cell to wing tip; cu-v of hind wing 1.5 × length of second abscissa of M+Cu; S2-6 with distinct emarginations; S2 deep almost semicircular; S3 circa three times as wide as deep; S4 circa six times as wide as deep; S5 circa six times as wide as deep; S6 with apicomedial emargination circa 2.5 × as wide as deep; S6 medially with raised posteriorly directed rounded ridge.

*Genitalia*: penis valves with well extended shoulders; volsella short, with ten setae (Fig. 5H); gonocoxa laterally with a few short setae; apex of gonocoxa constricted with a dorsal rounded lobe; outer gonostylus robust, with rounded basal lobe at inner ventral side, and row of long robust setae on inner surface dorsally and dense fine setae ventrally; inner gonostylus very small, reduced to transparent lobe (Fig. 5G); S7 (Fig. 5E); S8 broad with rounded apex bearing fine setae, and a few strong setae around midline (Fig. 5F).

***Pubescence***: Head white and pale orange around ocelli and on vertex; scutum, scutellum and metanotum orange and some darker hairs in the area between the tegulae; mesosoma laterally orange, ventrally orange and dark brown; fore leg pale orange; mid and hind legs: coxa, trochanter and inner surface of femur, tibia and metatarsus with dark brown pubescence, outer surface orange; metasomal terga with pale orange hairs; T2 anteriorly with narrow band of black hairs; S1-S5 with narrow rows of pale orange hairs on posterior margins increasing in length laterally; apicomedial area of S3 and S4 with distinct round patch of black, forward-directed bristles; S5 with long brown branched hairs around the emargination, and long plumes of orange hairs laterally; S6 with small patch of orange hairs posterior to rounded ridge, short and dense pale hairs in the emargination.

***Colouration***: Integument black-brown, apart from: top of femur, tibia and tarsus orange; sterna brown, most of metasoma orange; scape pale yellow below; flagellum dark brown; labrum pale yellow with small black dot near dorsolateral corners; clypeus pale yellow; supraclypeal area pale yellow; paraclypeal area pale yellow; mandible pale yellow at base, greyish at tip; proboscis orange.

**Female** holotype (WAM5457): Body length 14 mm, forewing length 10.2 mm, head width 4.9 mm.

***Structure***: Inner orbits of eyes diverging above; head wider than long; clypeal protuberance in profile 0.74 × eye width; mandible with subapical tooth; F1 equal to combined length of next 3 flagellomeres; F1 0.86 × as long as scape; F2 0.67 × as long as F3; F3-F10 circa as long as wide; last flagellomere 0.63 × as long as F1; marginal cell length 0.61 × distance from apex of cell to wing tip; cu-v of hind wing 1.4 × length of second abscissa of M+Cu; S6 with narrow raised area and short transverse posterior ridge (Fig. 5L).

***Pubescence***: Head white with pale orange hairs around ocelli and on vertex; scutum, scutellum and metanotum orange and some darker hairs between the tegulae; mesosoma laterally and ventrally pale orange; fore leg pale orange hairs; mid and hind legs: coxa, trochanter and inner surface of femur, tibia and metatarsus dark brown, outer surface orange; metasomal terga pale orange, T2 with some black hairs in anterolateral corners; T5 fimbria brown medially; T6 with strong brown hairs flanking the pygidial plate; S1-S5 with narrow rows of pale orange hairs on posterior margins increasing in length laterally; S2–S4 with some black hairs medially.

***Colouration***: Integument of head and mesosoma black-brown, apart from: scape orange-brown; F1–F2 brown remaining segments brown below, black above; labrum yellow with small brown dots near dorsolateral corners; clypeus yellow with undefined narrow brown marks; supraclypeal area with pale yellow triangle; paraclypeal area with pale yellow mark along epistomal suture; mandible pale yellow at base, black at tip; proboscis orange; top of femur, tibia, tarsus orange.

**Table:** **Phenology.**

**Month**:	**Jan**	**Feb**	**Mar**	**Apr**	**May**	**Jun**	**Jul**	**Aug**	**Sep**	**Oct**	**Nov**	**Dec**
No. of records:	0	0	0	0	0	0	0	11	3	0	0	0

#### Flower records.

*Eremophila maculata* ?, *Stemodia grossa* (Scrophulariaceae), *Trichodesma zeylanicum* (Boraginaceae), *Pityrodia augustensis* (Chloanthaceae).

#### Distribution.

Figure 5I.

#### Etymology.

The specific epithet refers to the orange habitus of the species.

### Amegilla (Asaropoda) batleyi

Taxon classificationAnimaliaHymenopteraApidae

Leijs, sp. nov.

DE425299-5F92-536D-B003-25211CA77F26

http://zoobank.org/D3B4AA5A-B6A0-48AF-9319-013FE836DF42

Figure 6A–M

#### Specimens examined.

3 males, 2 females.

#### Types.

Holotype, male, 7 km NW Barkly Roadhouse, NT (19.6697S; 135.7675E), 14 May 2008, M Batley, sweep sample, AM K.361599;

Allotype, female, 29 km South of Tennant Creek, NT (19.8108S; 134.2331E), 20 May 1973, TF & CA Houston, on *Eucalyptus*, WAM 5471;

Paratype, male WAM 5473, female WAM 5472, same locality data as allotype.

#### Diagnosis.

**Figure 6.**
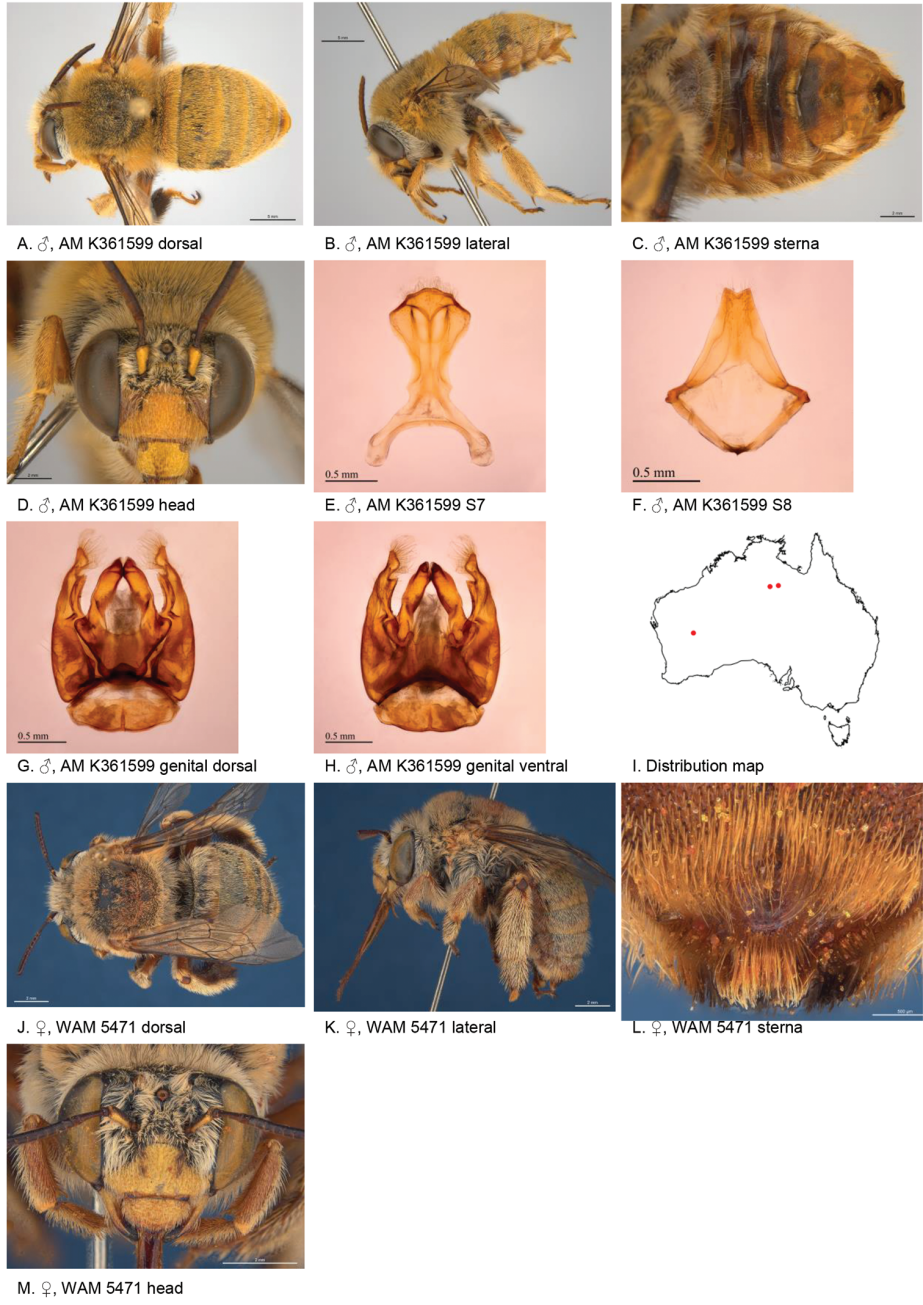
Amegilla (Asaropoda) batleyi Leijs, sp. nov.

Male with rectangular patch of pale bristles on S4 and shallow emarginated apicomedial area of S5. Female small paraclypeal mark in lower corners, supraclypeal mark present, tibial scopa grey-white, T2 without black hairs anteriorly, process on S6 openly punctate, little defined posteriorly (Fig. 6L).

#### Description.

Male holotype (AM K361599): Body length 14 mm, forewing length 9 mm, head width 4.9 mm.

***Structure***: Inner orbits of eyes parallel; head wider than long; clypeal protuberance in profile 0.6 × eye width; mandible with weak subapical tooth; F1 slightly shorter than combined length of F2+3; F1 0.75 × as long as scape; F2 equal in length to F3; F3-F10 circa as long as wide; last flagellomere 0.5 × length of F1; marginal cell length 0.79 distance from apex of cell to wing tip; cu-v of hind wing equal to length of second abscissa of M+Cu; S5 with apicomedial emargination circa six times as wide as deep, S6 with apicomedial emargination 5.1 × as wide as deep, S6 marginal zone narrow, smooth, with open punctures and hairs, medially without patch of bristles (Fig. 6C).

*Genitalia*: penis valves laterally with extended rounded shoulders; volsella with circa six long setae (Fig. 6H); gonocoxa laterally with four long setae and six short setae; apex of gonocoxa ventrally with acute process (Fig. 6H); outer gonostylus short and truncated, with long setae on inner and outer surface; inner gonostylus well developed, circa as long as width of outer gonocoxa at apex, with long setae (Fig. 6G); S7 (Fig. 6E); S8 triangular, apex with sharp corners and emarginated (Fig. 6F).

***Pubescence***: Head white hairs and scattered dark hairs on frons and vertex, genae white, scutum, scutellum and metanotum light brown with scattered dark brown hairs; mesosoma laterally and ventrally with pale to white, fore leg with long pale hair on outer surface; fore leg inner surfaces of tibia and tarsus with short orange-brown hair; mid femur anterior and posterior surfaces pale; mid tibia pale; mid metatarsus pale, last segments orange, mid tarsus inner surface orange with two dark brown streaks, hind leg outer surface of femur, tibia and metatarsus pale, hind leg inner surface black; metasomal terga with pale ochre adpressed hairs intermixed with scattered erect black hairs on T1-T3 and white erect hairs on T4-T6, T2 anteriorly without dark hairs, T7 short white pubescence, S1-S5 with pale apical fringes laterally only; S3 with open row of simple setae on premarginal line, S4 apicomedial area with rectangular patch of pale apically directed bristles circa 1/3 of sternal width Fig. 6C); S5 with dark branched hairs on marginal zone around emargination, flanked with pale hairs on disc, S6 with smooth marginal zone and sparse to openly placed branched hairs.

***Colouration***: Integument of head black apart from: scape yellow below, labrum yellow with brown dots in dorsolateral corners, clypeus orange-yellow, supraclypeal area orange-yellow, paraclypeal area brown, mandible yellow with dark brown tip, proboscis orange; mesosoma black, metasoma brown with orange translucent marginal zones; legs orange apart from coxae, trochanters and basal half of femur brown.

**Female** allotype (WAM 5471): Body length 14 mm, forewing length 10 mm, head width 5.6 mm.

***Structure***: Inner orbits of eyes slightly diverging above; head wider than long; clypeal protuberance in profile 0.67 × eye width; mandible with weak subapical tooth; F1 equal to combined length of next 3.3 flagellomeres; F1 0.93 × as long as scape; F2 0.75 × as long as F3; F3-F10 circa as long as wide; last flagellomere 0.57 × length of F1; marginal cell length 0.42 × the length of costal vein R; cu-v of hind wing 0.86 × length of second abscissa of M+Cu; S6 with broadly rounded area raised posteriorly (Fig. 6L).

***Pubescence***: Head white with scattered dark hairs on frons and vertex; genae white; scutum, scutellum and metanotum light ochre with scattered dark brown hairs; mesosoma laterally and ventrally with light ochre hairs under the wing bases, remaining hairs white; fore leg with pale hair on inner and outer surface; mid and hind legs whitish on outer surfaces, black-brown on inner surfaces, including black hairs on hind trochanters, mid tibia, mid metatarsus and mid tarsus; metasomal terga with pale ochre adpressed hairs intermixed with scattered erect black hairs on T1–T3 and white erect hairs on T4–T6; T2 anteriorly without dark hairs; T5 with brown prepygidial fimbria, T6 with strong orange-brown hairs flanking the pygidial plate; S1–S5 with ochre erect hairs on posterior margins.

***Colouration***: Integument of head black, apart from scape yellow below; flagellum orange below, brown above; labrum pale yellow with translucent dots in dorsolateral corners; clypeus pale yellow with two vague subparallel brown linear marks; supraclypeal area with small pale yellow mark; paraclypeal area with small pale yellow mark in ventral corner; mandible pale yellow at base, black at tip; proboscis orange-brown; mesosoma black; metasoma brown with orange transparent marginal zones; legs orange apart from coxae, trochanters and basal half of femur brown.

**Table:** **Phenology.**

**Month**:	**Jan**	**Feb**	**Mar**	**Apr**	**May**	**Jun**	**Jul**	**Aug**	**Sep**	**Oct**	**Nov**	**Dec**
No. of records:	0	0	0	0	4	0	0	0	0	0	0	0

#### Flower records.

*Eucalyptus* (Myrtaceae).

#### Distribution.

Figure 6I.

#### Etymology.

The specific epithet refers to Michael Batley, the collector of the type specimen, in honour of his contribution to Australian bee taxonomy and identification services.

### Amegilla (Asaropoda) bombiformis

Taxon classificationAnimaliaHymenopteraApidae

(Smith, 1854)

40B49D06-11C1-5B03-B17E-5EB264B57F9A

Figure 7A–M

Saropoda bombiformis Smith, 1854: 318.
Asaropoda anomala Cockerell, 1929: 15, **syn. nov.**
Asaropoda imitata Rayment, 1951: 74, **syn. nov.**
Asaropoda punctata Rayment, 1931: 182, **syn. nov.**
Asaropoda rubricata Rayment, 1951: 76, **syn. nov.**
Asaropoda rubricata dentata Rayment, 1951: 76, **syn. nov.**
Asaropoda rufa Rayment, 1931: 181, **syn. nov.**

#### Specimens examined.

149 males, 340 females

#### Types.

Lectotype of *bombiformis*: female, Richmond River, NSW, BMNH 99-303.

Holotype of *A. anomala*: male, Brisbane, AMNH.

Holotype of *A. imitata*: female, New South Wales, ANIC.

Holotype of *A. punctata*: male, Sydney, NSW, whereabouts unknown.

Holotype of *A. rubricata*: male, Lismore, NSW, whereabouts unknown.

Holotype of *A. rubricata dentata*: male, Sydney, 7.2.43, O. Dawson, No 702, ANIC, the specimen is lacking terga and sterna from S5 onwards.

Holotype of *A. rufa*: female, Enoggera, QLD, whereabouts unknown.

**Decision for synonymy** was based on examination of the type specimens, when available, combined with original descriptions and diagnostic characters.

#### Diagnosis.

**Figure 7.**
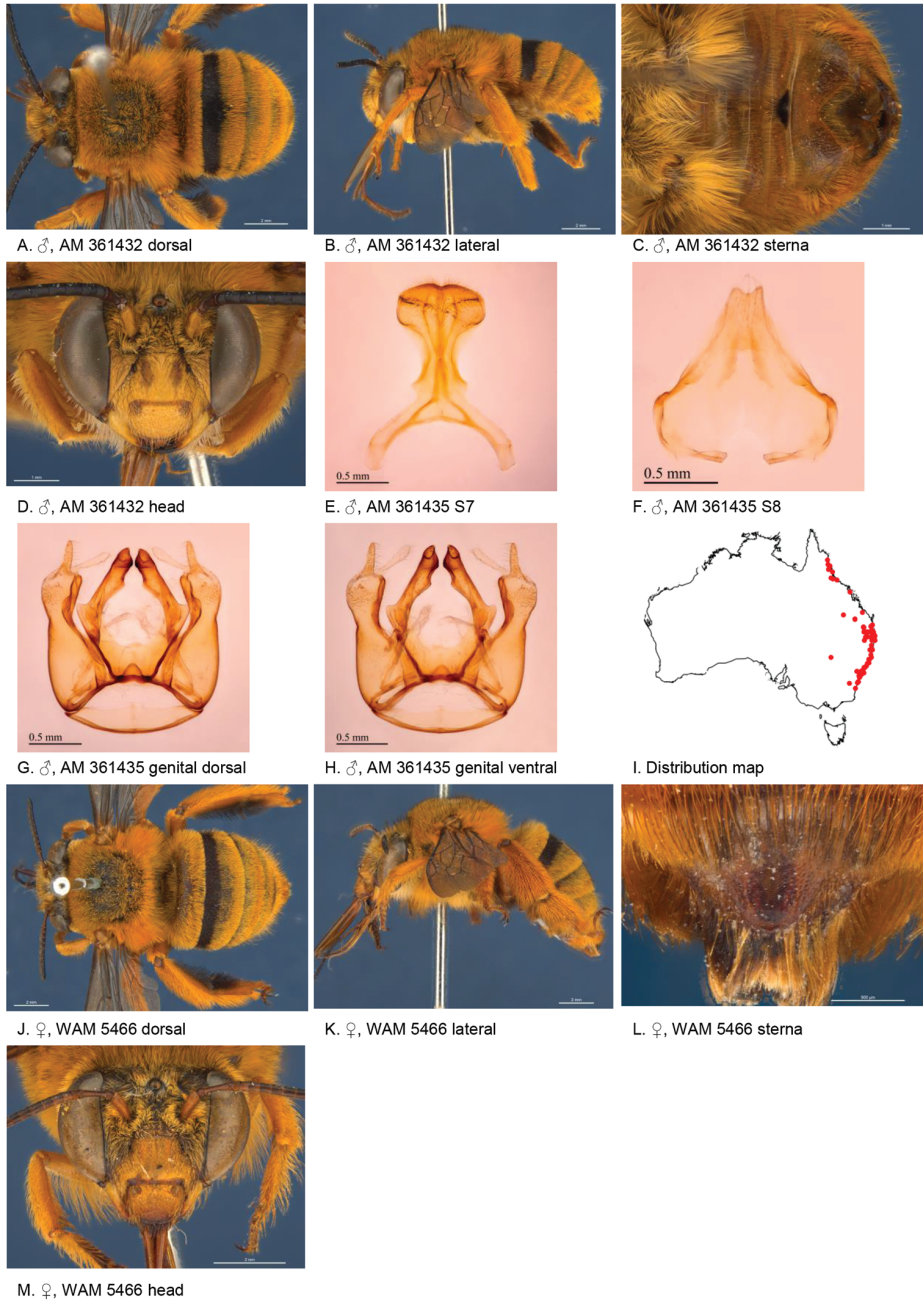
Amegilla (Asaropoda) bombiformis (Smith, 1854).

Both sexes with orange iridescent metasomal pubescence. Male with small triangular patch of black bristles on S4 and wide triangular emarginated apicomedial area of S5. Female paraclypeal and supraclypeal marks absent, tibial scopa orange, T2 with black hairs anteriorly, process on S6 reticular striate, broadly defined posteriorly (Fig. 7L).

#### Redescription.

Male (AM 361432): Body length 12.5 mm, forewing length 9.7 mm, head width 4.7 mm.

***Structure***: Inner orbits of eyes diverging above; head wider than long; clypeal protuberance in profile 0.70 × eye width; mandible with subapical tooth; F1 equal to combined length of next 1.6 flagellomeres; F1 0.7 × as long as scape; F2 0.63 × as long as F3; F3-F10 1.5 × as long as wide; last flagellomere 1.3 × as long as F1; marginal cell length 0.81 distance from apex of cell to wing tip; cu-v of hind wing 0.75 × as long as second abscissa of M+Cu; S5 with apicomedial emargination triangular, circa 2.3 × as wide as deep, with wide translucent posterior margins; S6 with apicomedial emargination circa 4.4 × as wide as deep, emargination preceded by smooth median area widening posteriorly.

*Genitalia*: penis valves slender, with well extended shoulders; volsella slightly rectangular with 6 short setae (Fig. 7H); gonocoxa laterally with a few short setae; apex of gonocoxa broadened without extended lobes; outer gonostylus small and narrow, with few small setae; inner gonostylus a slightly longer than outer gonostylus, directed inwards with a few scattered setae (Fig. 7G); S7 (Fig. 7E); S8 emarginated, with few setae (Fig. 7F).

***Pubescence***: Head with genae white, vertex pale yellow intermixed with black hairs, labrum white, clypeus and paraclypeal area black, face pale orange with black hairs around median ocellus and ocellocular area; scutum, scutellum and metanotum orange intermixed with black hairs; mesosoma laterally and ventrally orange, paler towards ventral side; fore and mid legs pale orange, mid femur, mid and hind tibia and tarsus pale orange on outer surfaces, femur, and tibia and tarsus on inner surfaces black-brown; hind leg outer surface of femur, tibia and metatarsus orange; hind leg inner surface of femur, tibia and metatarsus black; metasomal terga orange, T1 intermixed with black hairs, T2-T6 with adpressed hairs with metallic shine; T2 anteriorly with wide band of black hairs; S2-S4 with fringes of pale yellow branched hairs on posterior margins; S4 apicomedial area with small triangular patch of ventrally directed black bristles circa 1/5 of sternal width; S5 with area preceding the emargination with a sparse row of pale orange branched hairs, lateral corners with plumes of long orange hairs; S6 with dense patches of erect branched hairs flanking the median smooth area.

***Colouration***: Integument of head, mesosoma and metasomal terga including posterior margins black apart from scape brown above, yellow below; flagellum black; labrum yellow with translucent dots near dorsolateral corners; clypeus yellow with two brown longitudinal patches on either side of the midline; supraclypeal area yellow; paraclypeal area yellow; mandible yellow at base, black at tip; proboscis orange; sterna and legs orange, apart from hind femur brown.

**Female** redescription (WAM 5466): Body length 15 mm, forewing length 11.3 mm, head width 5.8 mm.

***Structure***: Inner orbits of eyes diverging above; head wider than long; clypeal protuberance in profile 0.65 × eye width; mandible with subapical tooth; F1 equal to combined length of almost next three flagellomeres; F1 circa as long as scape; F2 0.85 × as long as F3; F3-F10 1.14 × as long as wide; last flagellomere 0.62 × as long as F1; marginal cell length 0.9 distance from apex of cell to wing tip; cu-v of hind wing 0.82 × as long as second abscissa of M+Cu; S6 with broad parabolically raised area (Fig. 7L).

***Pubescence***: Head with genae grey-white, vertex pale yellow intermixed with black hairs, labrum pale orange, clypeus and paraclypeal area black, face pale orange with black hairs around median ocellus and ocellocular area; scutum, scutellum and metanotum orange intermixed with black hairs; mesosoma laterally and ventrally orange, paler towards ventral side; fore and mid legs pale orange, mid femur and hind leg outer surface of femur, tibia and metatarsus orange; hind leg inner surface of femur, tibia and metatarsus black; metasomal terga orange intermixed with black hairs on T1; T2-T5 adpressed hairs with metallic shine; T2 anteriorly with wide band of black hairs; T2-T4 with dark hairs laterally below the gradulus; T5 with orange-brown prepygidial fimbria; T6 with strong light-brown hairs flanking the pygidial plate; S1-S5 with fringes of pale branched hairs on posterior margins of S2-S4, S3 and S4 laterally denser and brown.

***Colouration***: Integument of head black, apart from: scape orange, flagellum brown below and black above; labrum orange yellow; clypeus orange yellow; supraclypeal area vague orange ventrally; paraclypeal area black; mandible yellow at base, black at tip; proboscis orange-brown; mesosoma and metasomal terga black, sterna and legs orange.

**Table:** **Phenology.**

**Month**:	**Jan**	**Feb**	**Mar**	**Apr**	**May**	**Jun**	**Jul**	**Aug**	**Sep**	**Oct**	**Nov**	**Dec**
No. of records:	53	66	168	112	6	2	2	2	3	5	12	21

#### Flower records.

*Lantana, Duranta* (Verbenaceae). Considering the large number of records (*N* = 491) there is remarkably little information recorded about flower visitation. However, photos on the internet show that a large variety of plant species are visited.

#### Distribution.

Predominantly East coast, Figure 7I.

### Amegilla (Asaropoda) calva

Taxon classificationAnimaliaHymenopteraApidae

(Rayment, 1935)

01EFA0AA-49C5-5512-A7E2-95AE32475400

Figure 8A–M

Asaropoda calva Rayment, 1935: 712.
Amegilla (Asaropoda) paracalva Brooks 1993: 279, **syn. nov.**

#### Specimens examined.

55 males, 49 females

#### Types.

Holotype of *A. calva*, female, Davis Creek, NSW, ANIC.

Holotype of *A. paracalva*, male, 16 km WSW of Lyons River HS (24°38’S; 115°20’E) 30 Aug.-1 Sep. 1980, CA Howard & TF Houston, 344-27, Reared from brood cell. Became adult 5 Sept. 1983, WAM 90/879, Reg no. E5430.

**Decision for synonymy** was based on careful examination of the characters mentioned by Brooks (1993) to distinguish *A. calva* from *A. paracalva*. The types of *A. paracalva* were from freshly emerged reared specimens. The creamy integumental facial marks of these specimens suggests that they were killed before they had fully coloured out to the adult pale yellow observed in other specimens examined. Further, while the male apicomedial emargination of S5 and S6 varied slightly among specimens, consistent differences between East and West Australia, as suggested by Brooks, were not found. Additionally, there were no differences in male genital characters between East and West Australian specimens. The number of specimens examined by Brooks to support his decision to raise the new species is unclear.

#### Diagnosis.

**Figure 8.**
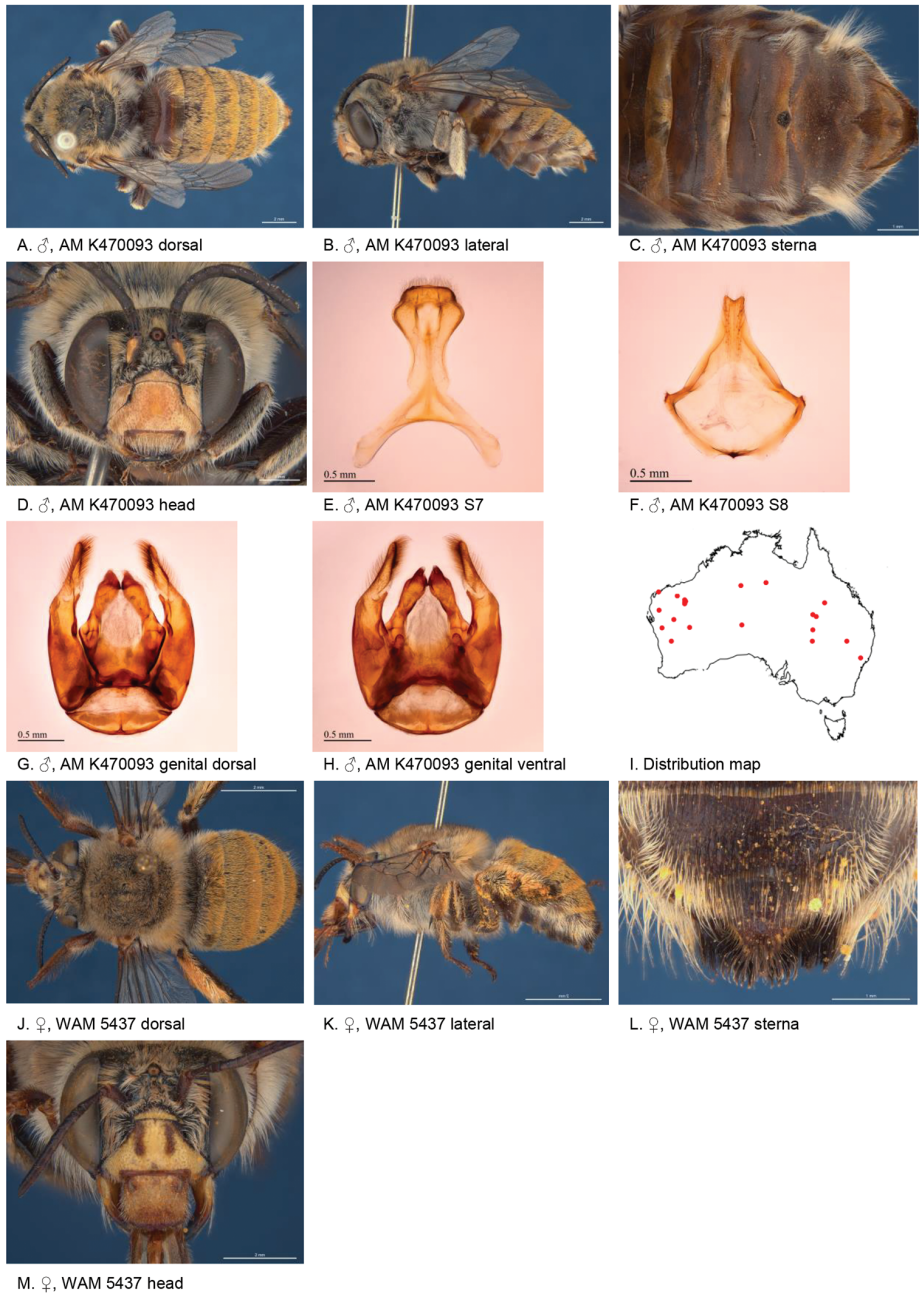
Amegilla (Asaropoda) calva (Rayment, 1935).

Male with small round patch of black bristles on S4 and triangular emarginated apicomedial area of S5. Female paraclypeal mark absent, supraclypeal mark present, tibial scopa white, T2 without black hairs anteriorly, process on S6 irregular striate and punctate, not defined posteriorly (Fig. 8L).

#### Redescription.

Male holotype *paracalva*: Body length 15 mm, forewing length 10 mm.

***Structure***: Inner orbits of eyes parallel; head wider than long; clypeal protuberance in profile 0.67 × eye width; mandible with weak subapical tooth; F1 equal to combined length of next 1.5 flagellomeres; F1 0.68 × as long as scape; F2 equal to 0.77 length of F3; F3-F10 gradually increasing in length, subequal to length of F1; distance between posterior ocelli 1.1 ocellocular distance; distance from median ocellus to posterior ocellus 0.68 ocellocular distance; marginal cell length 0.82 distance from apex of cell to wing tip; cu-v of hind wing equal to length of second abscissa of M+Cu; S5 with apicomedial emargination narrow (Figure 8C); S6 with apicomedial emargination narrow (Figure 8C).

*Genitalia*: penis valves laterally with rounded shoulders; volsella with circa 12 long setae (Fig. 8H); gonocoxa laterally with setae lacking; outer gonostylus long, circa as long as width of penis valve base, with long setae on inner and outer surface; inner gonostylus very small, circa as long as width of outer gonostylus at apex (Fig. 8G); S7 (Fig. 8E); S8 apex slender, emarginated (Fig. 8F).

***Pubescence***: Head with pale buff hair, lighter on lower half; scutum with light orange brown and scattered black hairs; scutellum and metanotum with light orange-brown hair; mesosoma laterally and ventrally with pale hair; fore leg with pale hair on outer surface; fore leg inner surfaces of tibia and tarsus with dark orange-brown hair; mid femur anterior and posterior surfaces pale; mid tibia pale; mid metatarsus with mixture of dark and pale hair; mid tarsus all dark on inner surface; hind leg outer surface of femur, tibia and metatarsus pale, hind leg inner surface dark; metasomal terga with adpressed orange brown hair that is lighter and longer laterally but with a few dark hairs laterally on T6; T2 anteriorly with a few black hairs in lateral corners, T7 apicolaterally with dark dense patch; S1-S5 with pale apical fringes which are longer laterally; S4 apicomedial area with round patch of black apically directed bristles; S6 with basal band of pale hair.

***Colouration***: Integument black, with pale yellow facial marks on mandibular base, labrum, clypeus, paraclypeal and supraclypeal areas and anteriorly on scape; proboscis reddish-brown.

**Female** redescription (SAMA RL0462): Body length 16 mm, forewing length 11 mm, head width 5.3 mm.

***Structure***: Inner orbits of eyes parallel; head wider than long; clypeal protuberance in profile 0.63 × eye width; mandible with weak subapical tooth; F1 equal to combined length of next 2.7 flagellomeres; F1 circa as long as scape; F2 equal to 0.92 length of F3; F3-F10 circa 1.15 × as long as wide; last flagellomere 0.6 length of F1; distance between posterior ocelli circa equal to ocellocular distance; distance from median ocellus to posterior ocellus 0.64 ocellocular distance; marginal cell length 0.69 × distance from apex of cell to wing tip; cu-v of hind wing 1.26 × length of second abscissa of M+Cu; S6 with small raised ridge (Fig. 8L).

***Pubescence***: Head white, with some grey hairs on vertex; scutum, scutellum and metanotum grey intermixed brown hairs; mesosoma laterally and ventrally white; fore leg: outer posterior surface of fore tarsus with long curved dark hairs; fore leg inner surfaces of tibia and tarsus with dark hair; mid femur anterior and posterior surfaces pale; mid tibia outer surface pale with dark band on apico-posterior surface; mid metatarsus with scattered white hairs on anterior surface; mid tarsus with white posterior band; hind leg outer surface of femur, tibia and metatarsus white except anterior edge of tarsus black; scopa white; hind leg inner surface black; metasomal terga with pale yellow adpressed hairs; white hairs laterally below gradulus, T2 anteriorly without dark hairs, T5 with brown-black prepygidial fimbria, T6 all black; S1-S5 with long dark hairs medially, pale hairs laterally.

***Colouration***: Integument head black, apart from: scape brown; flagellum brown below dark above, labrum ivory with translucent dots in dorsolateral corners, clypeus ivory with two linear brown patches in dorsolateral corners leaving wide ivory midline; supraclypeal area ivory, paraclypeal area black, mandible ivory at base brown-black at tip, proboscis orange brown; mesosoma black, metasoma brown with wide orange translucent posterior margins.

**Table:** **Phenology.**

**Month**:	**Jan**	**Feb**	**Mar**	**Apr**	**May**	**Jun**	**Jul**	**Aug**	**Sep**	**Oct**	**Nov**	**Dec**
No. of records:	2	0	0	0	4	1	0	24	27	26	8	4

#### Flower records.

*Eremophila polyclada, Eremophila polyclada bignoniiflora, Eremophila, stenophylla, Eremophila maculata* (Scrophulariaceae), *Trichodesma zeylanica* (Boraginaceae), *Cassia* (Fabaceae).

#### Distribution.

When Brooks (1993) described *A. paracalva* he mentioned that *calva* was restricted to New South Wales and Queensland and *A. paracalva* to Western Australia. As we now understand *A. calva*, including *A. paracalva*, has a wide central distribution. Figure 8I.

### Amegilla (Asaropoda) crenata

Taxon classificationAnimaliaHymenopteraApidae

Leijs, sp. nov.

D4B325F2-4C20-55D4-9D50-AC1FD9AECF2C

http://zoobank.org/241585BB-FFE4-4468-9008-C42C1340761A

Figure 9A–M

#### Specimens examined.

(18 males, 15 females).

#### Types.

Holotype, male, Wongalara Station, NT (14.2042S; 134.1884E), 30 May 2012, R. Leijs, on *Melastoma* sp., SAMA RL2121

Allotype, female, same locality data as holotype, SAMA RL2122.

#### Diagnosis.

**Figure 9.**
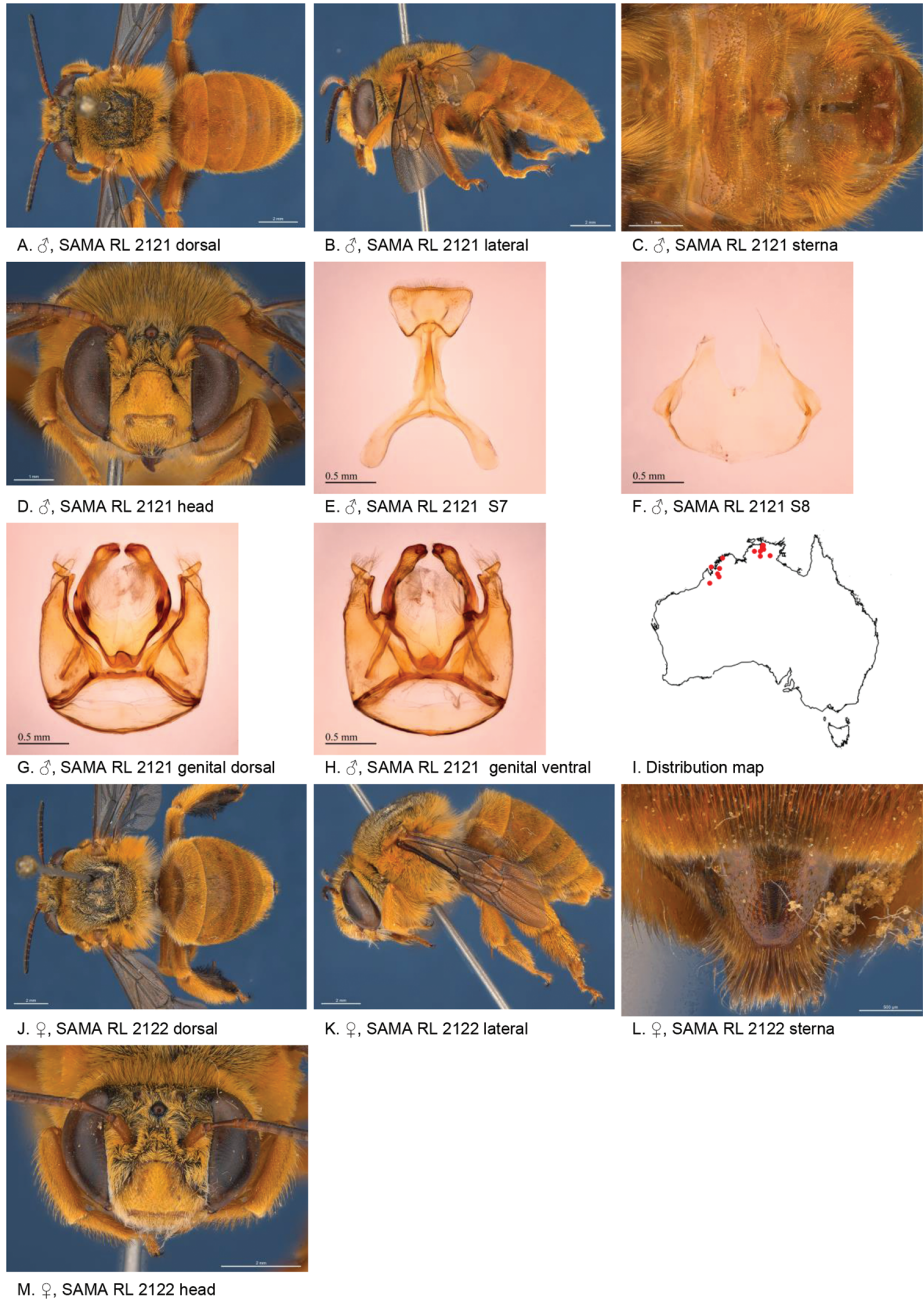
Amegilla (Asaropoda) crenata Leijs, sp. nov.

Both sexes body pubescence mostly orange. Male with small round patch of orange bristles on S4, a much smaller patch on S3 and a deep parallel sided emargination on apicomedial area of S5. Female paraclypeal mark absent, supraclypeal mark present, tibial scopa orange, T2 without black hairs anteriorly, process on S6 broadly parabolic and well defined shiny transverse lineo-reticulate and openly punctate (Fig. 9L).

#### Description.

Male holotype (SAMA 32-002684, RLb2121): Body length 13 mm, forewing length 10.1 mm, head width 4.7 mm.

***Structure***: Inner orbits of eyes almost parallel; head wider than long; clypeal protuberance in profile 0.66 × eye width; mandible with subapical tooth; F1 equal to combined length of next 1.7 flagellomeres; F1 0.78 × as long as scape; F2 0.67 × as long as F3; F3-F10 1.63 × as long as wide; last flagellomere 0.92 × as long as F1; marginal cell length 0.77 × distance from apex of cell to wing tip; cu-v of hind wing 0.82 × length of second abscissa of M+Cu; S5 with apicomedial emargination deep, with paralel sides, circa 2.9 as deep as wide (Fig. 9C); S6 without apicomedial emargination.

*Genitalia*: penis valves laterally broadly rounded; volsella short, rectangular, with 12 strong setae (Fig. 9H); gonocoxa laterally with numerous short setae; apex of gonocoxa with rounded lobes dorsally and ventrally; outer gonostylus very short, laterally extended, with openly placed setae; inner gonostylus very short, with openly placed setae; S7 (Fig. 9E).

***Pubescence***: Head white on lower half of genae, labrum, clypeus, along inner eye margins and around middle ocellus, remaining hairs pale orange, some long erect black hairs on clypeus, face and vertex; scutum, scutellum and metanotum with orange hairs intermixed with erect long black hairs; mesosoma laterally and ventrally with orange hairs, slightly paler ventrally; fore leg with pale orange hairs, fore leg inner surfaces of tibia and tarsus with pale orange hairs; mid and hind legs orange on outer surfaces, black on inner surfaces and on hind trochanters; metasomal terga T1 with pale orange erect hairs intermixed with few black hairs, remaining terga with adpressed orange slightly iridescent hairs; S1-S5 with rows of pale hairs on posterior margins; S3–S5 with patches of pale orange hairs on lateral corners increasing in length; S4 apicomedial area with distinct small round patch of orange forward directed bristles (Fig. 9C); S5 with simple orange brown hairs around the emargination; S6 with dense patches of branched orange hairs.

***Colouration***: Integument of head and mesosoma black; metasoma and legs orange; scape dark yellow below, brown above; F1-F3 orange, following segments brown, last three segments black above; labrum yellow with small brown marks in dorsolateral corners; clypeus yellow; anterior tentorial pits black; supraclypeal area yellow; paraclypeal area yellow; mandible yellow at base, black at tip; proboscis orange.

**Female** holotype (SAMA 32-002685, RLb2122): Body length 16 mm, forewing length 10.5 mm, head width 5.3 mm.

***Structure***: Inner orbits of eyes diverging above; head wider than long; clypeal protuberance in profile 0.61 × eye width; mandible with subapical tooth; F1 equal to combined length of next 3 flagellomeres; F1 circa as long as scape; F2 0.7 × as long as F3; F3-F10 1.2 × as long as wide; last flagellomere 0.57 × as long as F1; marginal cell length 0.76 × distance from apex of cell to wing tip; cu-v of hind wing 0.72 × length of second abscissa of M+Cu; S6 with broadly rounded area raised posteriorly (Fig. 9L).

***Pubescence***: Head with white hairs below, on lower half of genae, labrum, clypeus and around middle ocellus, remaining hairs pale orange, some long erect black hairs on clypeus, face and vertex; scutum, scutellum and metanotum with short grey hairs intermixed with a few longer black hairs; hairs on metanotum pale orange, propodeum white; mesosoma laterally and ventrally with orange hairs, slightly paler ventrally; all legs with orange hairs, apart from inner surface of hind tibia and metatarsus black; metasomal terga with T1 pale orange erect hairs intermixed with few black hairs; remaining terga with adpressed orange slightly iridescent hairs, T5 with apicomedial fimbria orange brown; T6 with strong brown hairs flanking the pygidial plate; S1-S5 with rows of orange hairs on posterior margins increasing in length laterally.

***Colouration***: Integument of head and mesosoma black; metasoma and legs orange; scape orange-brown, flagellum brown below, black above; labrum yellow; clypeus yellow, with two vague brown longitudinal patches; supraclypeal area with yellow triangle; paraclypeal area black; mandible yellow at base, greyish yellow at tip; proboscis orange.

**Table:** **Phenology.**

**Month**:	**Jan**	**Feb**	**Mar**	**Apr**	**May**	**Jun**	**Jul**	**Aug**	**Sep**	**Oct**	**Nov**	**Dec**
No. of records:	1	1	0	3	9	6	5	1	3	0	3	1

#### Remarks.

http://www.boldsystems.org/index.php/Public_RecordView?processid=AUSBS304-13, AUSBS305, AUSBS306, AUSBS307.

#### Flower records.

*Trichodesma zeylanicum* (Boraginaceae), *Melastoma* sp. (Melastomataceae), *Calytrix* sp. (Myrtaceae), *Acacia* (Fabaceae).

#### Distribution.

Figure 9L.

#### Etymology.

The specific epithet refers to the deep parallel medial incision in male S5.

### Amegilla (Asaropoda) dawsoni

Taxon classificationAnimaliaHymenopteraApidae

(Rayment, 1951)

E6C6AB81-7ABD-5474-BFE0-20F1F81CE795

Figure 10A–M

Asaropoda dawsoni Rayment, 1951: 77.

#### Specimens examined

(180 males, 164 females).

#### Types.

Holotype of *A. dawsoni*: male, Onslow, 2-8-46, O. Dawson, cotype, WA, MV T-11869. The female has not been formerly described and is described here.

#### Diagnosis.

**Figure 10.**
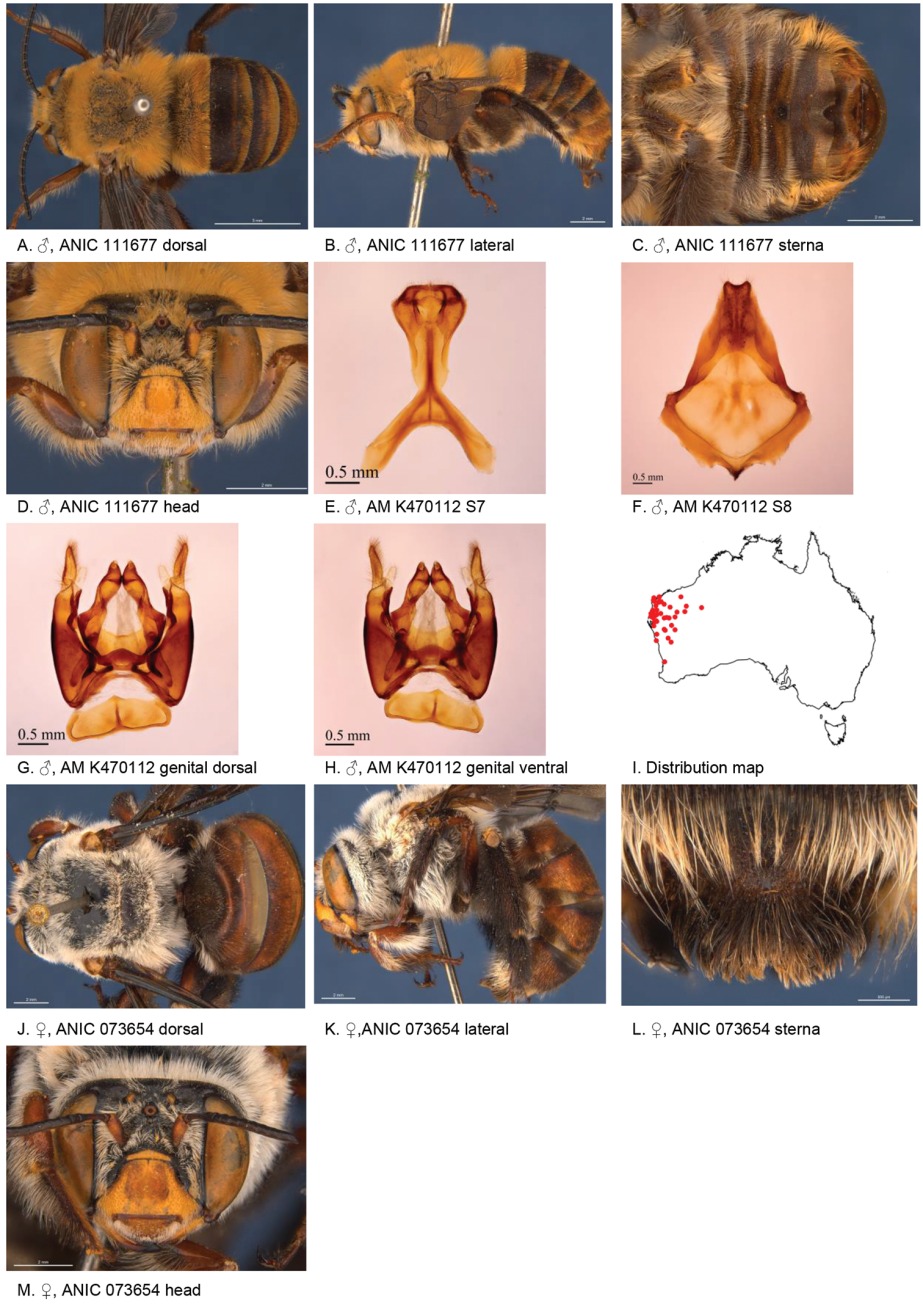
Amegilla (Asaropoda) dawsoni (Rayment, 1951).

The largest species. Sexes are colour-dimorphic: females head and mesosomal pubescence white, males light brown. Body sizes of males have a bimodal distribution, the largest males approximately as large as the females, small males circa ¾ the size. Male with round patch of dark bristles on S4, S5 with apicomedial emargination wide, parabolic. Female paraclypeal and supraclypeal mark present, tibial scopa black, process on S6 small irregular roughened, not well defined (Fig. 10L).

#### Redescription.

Male (VM Holotype T-11869): Body length 16 mm, forewing length 12.4 mm, head width 5.3 mm.

***Structure***: Inner orbits of eyes slightly diverging above; head wider than long; clypeal protuberance in profile 0.77 × eye width; labrum on ventral rim with two teeth; mandible with small subapical tooth; F1 almost equal to combined length of next 2 flagellomeres; F1 0.76 × as long as scape; F2 0.67 × as long as F3; F3-F10 almost twice as long as wide; last flagellomere 0.94 × as long as F1; marginal cell length 0.69 distance from apex of cell to wing tip; cu-v of hind wing 1.55 × as long as second abscissa of M+Cu; S5 with apicomedial emargination wide, parabolic, circa two times as wide as deep, S6 with apicomedial emargination very shallow and wide; T7 extended, broadly rectangular.

*Genitalia*: penis valves wide at base and well developed square shoulders; volsella large with 12 setae (Fig. 10H); gonocoxa laterally with a few short setae; apex of gonocoxa with inward-bent ridge and rectangular ventral lobe; outer gonostylus circa as long as penis valve base, with robust setae at inner surface; inner gonostylus circa half as long as outer gonostylus with slender setae (Fig. 10G); S7 (Fig. 10E); S8 apex broadly emarginated, some short slender setae along midline (Fig. 10F).

***Pubescence***: Head white on genae, labrum, clypeus, paraclypeal area and frons, pale yellow on vertex, around ocelli and antennal sockets; scutum with pale orange intermixed with a few black hairs; scutellum, metanotum and mesosoma laterally and ventrally pale orange, black ventrally; propodeum white; fore leg and trochanter with white hairs posteriorly, long pale orange hairs posteriorly on femur, tibia and metatarsus, with sparse short black hairs ventrally; tibia anteriorly with stiff brown hairs; middle legs with long white hairs posteriorly and black hairs anteriorly, hind leg coxa and trochanter white, remaining hairs black; metasomal terga with orange-brown on T1; T2-T7 black, with some orange hairs on posterior margins, lateral corners with plumes of white hairs; S1-S4 with sparse long white hairs on disks and rows of short branched white hairs on posterior margins; S5-S6 brown black hairs; S4 apicomedial area with two small adjacent patches of anteriorly directed black bristles; S5 with branched black hairs around the emargination; S6 with small patch of black hairs in the emargination.

***Colouration***: Integument of head and mesosoma black; metasomal terga dark red, posterior margins translucent orange, sterna and legs brown-orange; scape brown above, yellow below; flagellum black; labrum shiny, dark yellow with translucent dots near dorsolateral corners; clypeus shiny, dark yellow; supraclypeal area with triangular dark yellow mark; paraclypeal area with narrow dark yellow mark; mandible yellow at base, black at tip; proboscis orange-brown.

**Female** description Allotype (ANIC 32-073654): Body length 20 mm, forewing length 17.5 mm, head width 6.9 mm.

***Structure***: Inner orbits of eyes slightly diverging above; head wider than long; clypeal protuberance in profile 0.77 × eye width; labrum on ventral rim with two teeth; mandible with small subapical tooth; F1 equal to combined length of next three flagellomeres; F1 slightly longer than scape; F2 0.82 × as long as F3; F3-F10 1.38 × as long as wide; last flagellomere 0.58 × as long as F1; marginal cell length 0.68 distance from apex of cell to wing tip; cu-v of hind wing 1.40 × as long as second abscissa of M+Cu; S6 without raised area (Fig. 10L).

***Pubescence***: Head, scutum, scutellum, metanotum and mesosoma laterally and ventrally with white pubescence entirely; fore leg with white hairs, some intermixed long black hairs on inner surface of basal femur, metatarsus outer surface black, inner surface orange; mid and hind legs black with some white hairs on posterior rim of mid tibia and a few white hairs on inner rim of hind tibial scopa; metasomal terga T1 anteriorly with white hairs, on disk with brown hairs; T2-T5 hairs black; T1-T4 laterally below gradulus with white hairs; T6 with white hairs posteriorly; T2 anteriorly; T5 with black prepygidial fimbria, T6 with strong brown to black hairs flanking the pygidial plate; S1-S5 with black hairs on disks and rows of short branched white hairs on posterior margins.

***Colouration***: Head black, apart from: scape orange; labrum shiny, orange with translucent dots near dorsolateral corners; clypeus shiny, dark yellow, with vague large brown marks; supraclypeal area with wide triangular dark yellow mark; mandible dark yellow at base, black at tip; proboscis brown; mesosoma black; metasomal terga black anteriorly otherwise brown-red, posterior margins translucent orange; sterna and legs brown-orange.

**Table:** **Phenology.**

**Month**:	**Jan**	**Feb**	**Mar**	**Apr**	**May**	**Jun**	**Jul**	**Aug**	**Sep**	**Oct**	**Nov**	**Dec**
No. of records:	0	0	0	1	1	0	42	253	40	1	0	0

#### Remarks.

The ecology of the mating behaviour of this species has been studied in detail and is summarised in the introduction.

#### Flower records.

*Stemodia grossa* (Plantaginaceae), *Eremophila ‘crenulata*’, *Eremophila leucophylla*, *Eremophila longifolia* (Scrophulariaceae), *Trichodesma* (Boraginaceae), *Acacia* (Fabaceae).

#### Distribution.

Figure 10I.

### Amegilla (Asaropoda) epaphrodita

Taxon classificationAnimaliaHymenopteraApidae

Brooks, 1988

1749E8FA-102E-551B-A36D-26E9A4F570A5

Figure 11A–M

Amegilla (Asaropoda) epaphrodita Brooks, 1988: 554.

#### Specimens examined

(2 males, 5 females).

#### Type.

Holotype of *A. epaphrodita*: female, 15 km E. Mt Cahill, NT, ANIC.

#### Remarks.

The description of the female holotype of this species can be found in Brooks (1988), here the male allotype is described for the first time.

#### Diagnosis.

Pubescence black on T1-T3 (females) and T1-T4 (males), remaining terga white.

#### Description.

Male allotype ANIC Split Rock Qld. Note: Probably mislabelled, likely to be Ubir Rock NT: Body length 17 mm, forewing length 11.1 mm, head width 5.1 mm.

***Structure***: Inner orbits of eyes diverging above; head wider than long; clypeal protuberance in profile 0.62 eye width; mandible without subapical tooth; F1 equal to combined length of next 1.7 flagellomeres; F1 0.73 × as long as scape; F2 0.69 × as long as F3; F3-F10 1.2 × as long as wide; last flagellomere 1.09 × as long as F1; marginal cell length 0.81 × distance from apex of cell to wing tip; cu-v of hind wing 1.15 × length of second abscissa of M+Cu; S5 with apicomedial emargination 3.6 × as wide as deep, S6 with apicomedial emargination circa five times as wide as deep.

**Figure 11.**
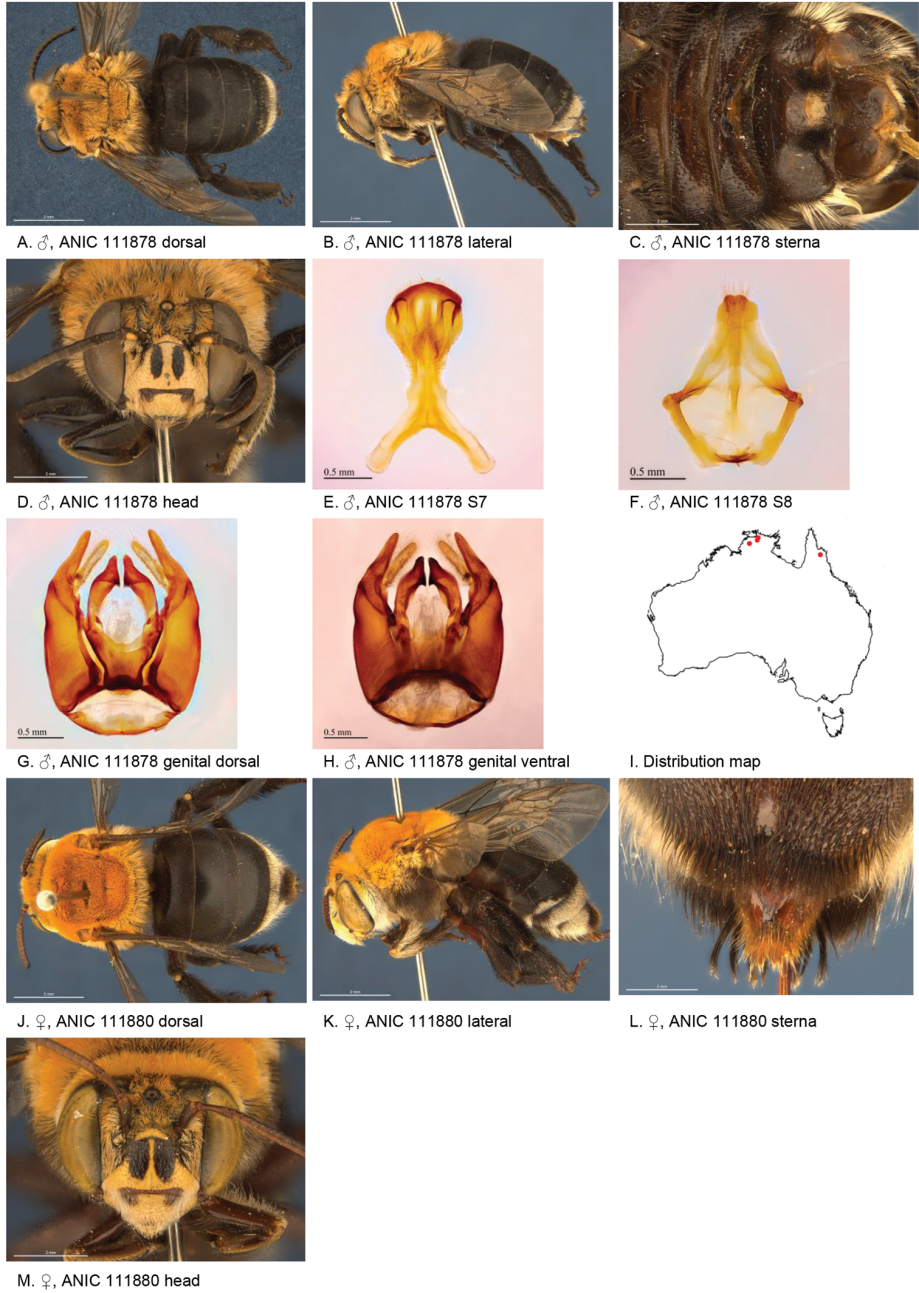
Amegilla (Asaropoda) epaphrodita Brooks, 1988.

*Genitalia*: penis valves with small shoulders; volsella small, with ten long setae (Fig. 11H); gonocoxa laterally with numerous small setae; apex of gonocoxa ventrally without process, but with notch near volsella (Fig. 11H); outer gonostylus long and robust with strong setae on inner surface; inner gonostylus circa as long as outer gonostylus, with numerous long setae (Fig. 11G); S7 (Fig. 11E); S8 apex wide slightly emarginate with fine setae on most of the surface (Fig. 11F).

***Pubescence***: Head white on labrum, clypeus and scape, pale hairs on genae and frons above antennal sockets; scutum, scutellum and metanotum with pale brown hairs; mesosoma laterally and ventrally with black hairs; pronotum, mespisternum anteriorly, and propodeum pale brown; fore leg with long white hairs on posterior parts of femur, tibia and tarsi; fore leg inner surfaces of tibia and tarsus and middle and hind legs with black hairs only; metasomal terga black, apart from white hairs covering T5-T7, and plumes on lateral corners of S3-S5; S4 apicomedial area with two small patches of downward directed black bristles, S5 with two longitudinal semi-parallel patches of posteriorly directed bristles flanking the emargination.

***Colouration***: Integument black, apart from: scape ivory patch below; labrum ivory with brown marks in dorsolateral corners; clypeus ivory, with two black parallel longitudinal marks; supraclypeal area with small ivory triangle; paraclypeal area ivory; mandible ivory at base, brown at tip; proboscis brown.

**Table:** **Phenology.**

**Month**:	**Jan**	**Feb**	**Mar**	**Apr**	**May**	**Jun**	**Jul**	**Aug**	**Sep**	**Oct**	**Nov**	**Dec**
No. of records:	2	3	2	0	0	0	0	0	0	0	0	0

#### Remarks.

The collection site Split Rock, Qld of the two males may be a labelling error, as it is outside the distribution of the species as we currently understand it.

#### Flower records.

no data.

#### Distribution.

Figure 11I.

### Amegilla (Asaropoda) flava

Taxon classificationAnimaliaHymenopteraApidae

(Friese, 1911)

0D816F9E-47E8-58B0-9D6E-0A1FBDD57626

Figure 12A–M

Anthophora flava Friese, 1911: 448.
Asaropoda cygni Rayment, 1931: 179, **syn. nov.**
Asaropoda rickae Rayment, 1951: 75, **syn. nov.**

#### Specimens examined.

23 males, 32 females

#### Types.

Holotype of *A. flava*, female, Freemantle, Australia, Friese Collection, ZMB.

Holotype of *A. cygni*, male, Swan River, WA, LJ Newman, Agriculture (Dept) Western Australia 28865.

Holotype of *rickae*, female, Bolgart, WA, May 1909, ANIC.

#### Decision for synonymy.

Examination of the holotype of *A. flava*, which was synonymised with *A. scymna* by Brooks (1988) without stating the reason for doing so, indicated its close similarity to the type of *A. rickae*, especially with respect to black supraclypeal and predominantly black paraclypeal areas, the absence of black hairs anteriorly on T2 and tibial scopa colour. Rayment’s male and female types are not different from the male and female specimens from Mount Wedge, SA, for which the sexes were associated using DNA.

#### Diagnosis.

**Figure 12.**
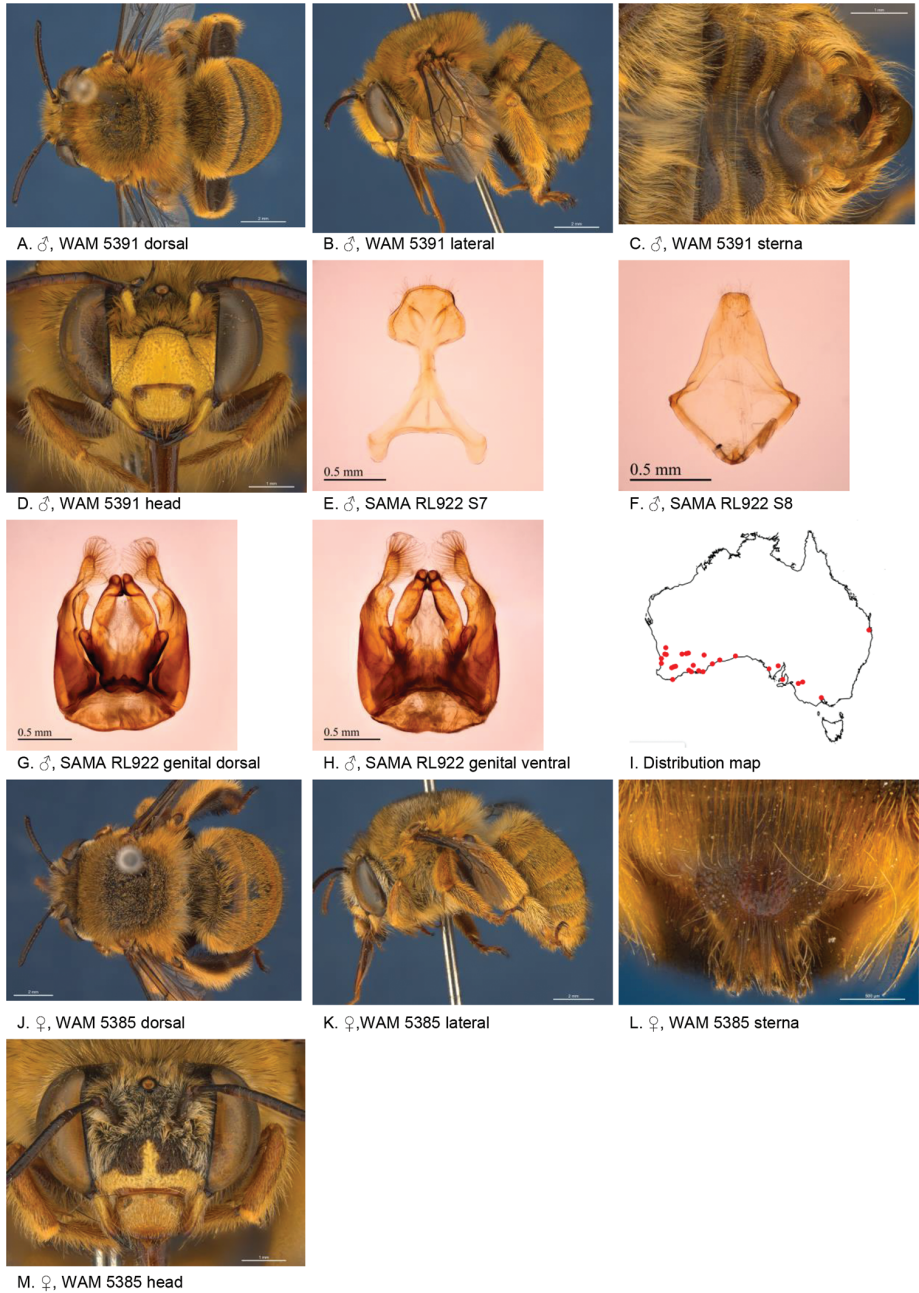
Amegilla (Asaropoda) flava (Friese, 1911).

Male with rectangular patch of light brown bristles on S4 and emarginated apicomedial area of S5 circa as wide as deep. Female paraclypeal and supraclypeal marks absent, tibial scopa pale yellow or ochre, T2 without black hairs anteriorly, process on S6 large roughly striate and punctate, not well defined posteriorly (Fig. 12L).

#### Redescription.

Male (WAM 5391): Body length 11.8 mm, forewing length 8.8 mm, head width 4.7 mm.

***Structure***: Inner orbits of eyes diverging above; head wider than long; clypeal protuberance in profile 0.57 × eye width; mandible with distinct subapical tooth; F1 equal to combined length of next 2.7 flagellomeres; F1 0.84 × as long as scape; F2 0.58 × as long as F3; F3-F10 1.23 × as long as wide; last flagellomere 0.63 × as long as F1; marginal cell length 0.72 distance from apex of cell to wing tip; cu-v of hind wing 0.6 × as long as second abscissa of M+Cu; S5 with apicomedial emargination parabolic shaped circa 2.3 × as wide as deep; S4 widely emarginated; S6 with apicomedial emargination circa 3.5 × as wide as deep.

*Genitalia*: penis valves with well extended shoulders; volsella slender with 12 long setae (Fig. 12H); gonocoxa laterally with a few short setae; apex of gonocoxa with almost rectangular ventral lobe; outer gonostylus circa as long as penis valve base, with long inwards bended setae at inner surface and apex; inner gonostylus 0.6 as long as outer gonostylus, slightly rectangular shaped with long setae at apex (Fig. 12G); S7 (Fig. 12E); S8 broad with almost straight apex (Fig. 12F).

***Pubescence***: Head white on labrum, clypeus, most of the genae and vertex; pale yellow on upper part of genae and, between eyes and antennal sockets, some intermixed black hairs on clypeus, around ocelli and vertex; scutum, scutellum and metanotum pale orange intermixed with black hairs; mesosoma laterally and ventrally pale orange, lighter towards venter; fore leg pale yellow; mid and hind legs black on inner surface and pale orange on outer surface; metasomal terga T1 anteriorly with long pale orange erect hairs intermixed with black hairs, posteriorly with adpressed pale orange hairs; T2-T6 with pale orange hairs; T2 anteriorly with entire narrow band of black hairs; T7 hairs brown; S1-S4 with fringes of long branched pale yellow hairs on posterior margins and openly spaced pale yellow simple hairs on disks; S4-S5 laterally with plumes of pale yellow hairs; S4 apicomedial area with two rectangular patches of anteromedially directed black bristles, together circa one third of sternum width; S5 with long brown branched hairs around the emargination; S6 with branched hairs medially and along anterior edges of marginal zone.

***Colouration***: Integument black-brown, posterior margins of terga and sterna translucent orange-brown; legs orange to brown; scape yellow below and dark brown above; flagellum brown below and dark brown above; labrum yellow with translucent dots near dorsolateral corners; clypeus yellow; supraclypeal and paraclypeal area yellow; mandible yellow at base black brown at tip; proboscis orange-brown.

**Female** redescription (WAM 5385): Body length 13 mm, forewing length 9.4 mm, head width 5.4 mm.

***Structure***: Inner orbits of eyes diverging above; head wider than long; clypeal protuberance in profile 0.59 × eye width; mandible with distinct subapical tooth; F1 equal to combined length of next 3.5 flagellomeres; F1 as long as scape; F2 0.8 × as long as F3; F3-F10 circa as long as wide; last flagellomere 0.47 × as long as F1; marginal cell length 0.70 distance from apex of cell to wing tip; cu-v of hind wing 0.63 × as long as second abscissa of M+Cu; S6 with broad parabolically raised area (Fig. 12L).

***Pubescence***: Head grey-white on labrum, clypeus and lower part of genae, face, vertex and upper part of genae pale orange some intermixed black hairs on clypeus, around ocelli and vertex; scutum, scutellum and metanotum with pale brown hairs intermixed with black hairs, grey on top of scutellum, and between tegulae; mesosoma laterally under wing base and ventrally pale brown, white towards venter, propodeum white; darker between coxae; outer surface of fore leg pale brown, darker on inner surface; mid and hind legs black on inner surface and pale orange on outer surface; scopa pale yellow, metasomal terga T1 anteriorly with long pale orange erect hairs intermixed with black hairs, posteriorly with adpressed pale orange hairs; T2-T5 with pale orange hairs; T2 anteriorly without adpressed black hairs; T5 with brown prepygidial fimbria; T6 with strong brown hairs flanking the pygidial plate; S2-S4 with fringes of long branched pale yellow hairs on posterior margins and openly spaced pale yellow simple hairs on disks; fringes and disks sometimes with dark hairs medially.

***Colouration***: Integument black-brown, apart from: posterior margins of terga and sterna translucent orange-brown; legs orange to brown; scape and flagellum brown; labrum pale yellow with translucent dots near dorsolateral corners; clypeus with pale yellow inverted T-shape; supraclypeal area black; paraclypeal area black without yellow mark; mandible pale yellow at base black brown at tip; proboscis orange-brown.

**Table:** **Phenology.**

**Month**:	**Jan**	**Feb**	**Mar**	**Apr**	**May**	**Jun**	**Jul**	**Aug**	**Sep**	**Oct**	**Nov**	**Dec**
No. of records:	16	7	5	0	1	0	0	0	0	2	4	4

#### Remarks.

The type of *A. flava* has a small yellow mark in ventral corner of the paraclypeal area, while in all other examined females the paraclypeal area is entirely black.

#### Flower records.

*Eremophila* (Scrophulariaceae), *Eucalyptus oleosa, Melaleuca subfalcata* (Myrtaceae).

#### Distribution.

Southern temperate Australia, Figure 12I.

### Amegilla (Asaropoda) frogatti

Taxon classificationAnimaliaHymenopteraApidae

Cockerell, 1914

91D1E89A-6011-5629-9447-67E16816EE81

Figure 13A–M

Anthophora preissi frogatti Cockerell, 1914: 468.

#### Specimens examined

(4 males, 3 females).

#### Type.

Holotype of *A. frogatti*: female, Brewarrina, NSW, 1914, WW Frogatt, NHM UK 010812649.

#### Diagnosis.

**Figure 13.**
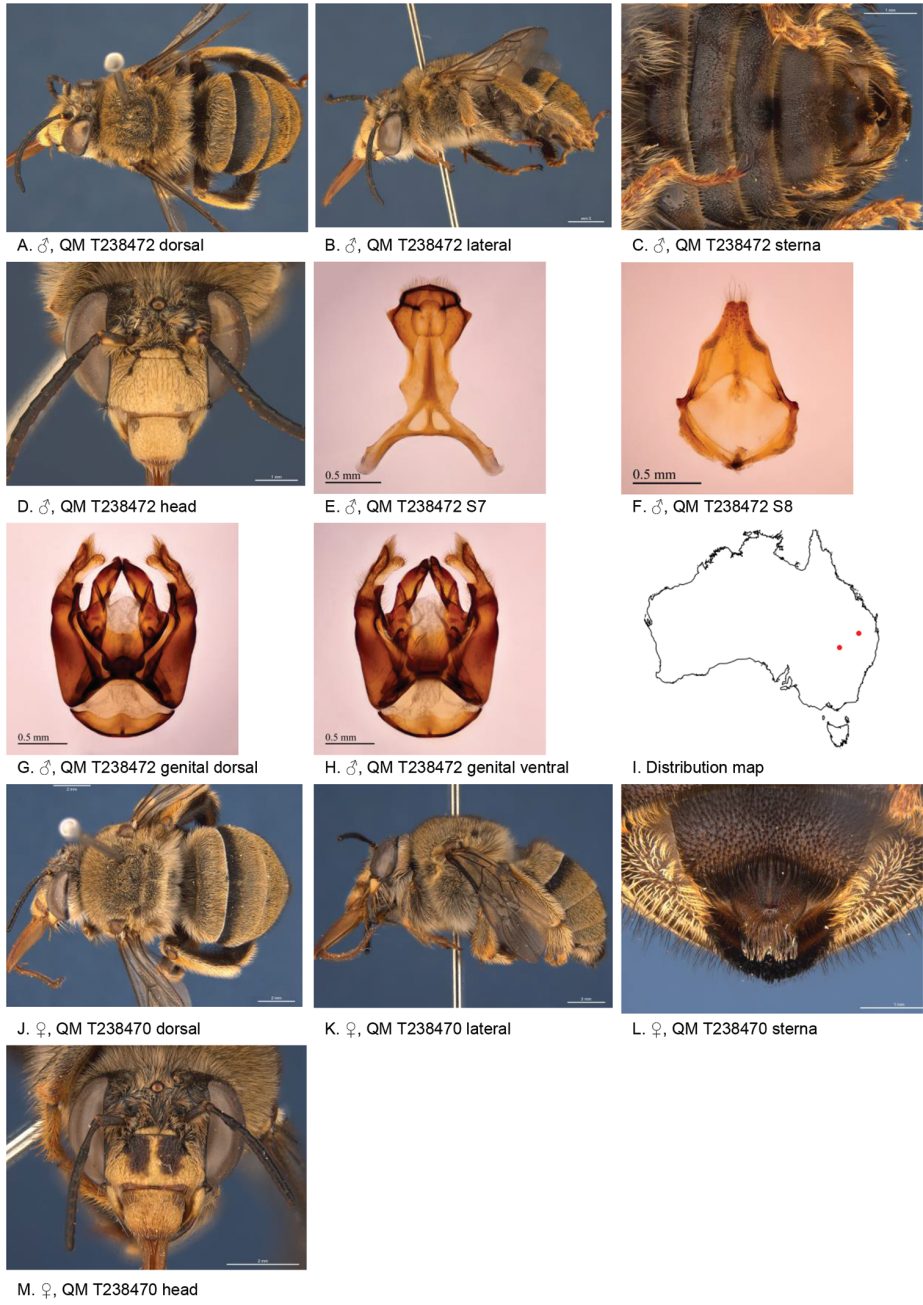
Amegilla (Asaropoda) frogatti (Cockerell, 1914).

Male with round patch of black bristles on S4 and shallow emarginated apicomedial area of S5. Female paraclypeal and supraclypeal marks present, tibial scopa grey, T2 with band of black hairs anteriorly, process on S6 small narrow transverse lineo-reticulate, shiny posteriorly, not well defined (Fig. 13L). Overall appearance grey, as in *preissi*.

#### Decision for synonymy.

*Anthophora preissi frogatti* was synonymised by Brooks, 1988 with *preissi* without stating the reason for doing so. Examination of the types of *A. preissi* and *A. preissi frogatti* clearly indicate different species, particularly because of the absence of yellow integument of the para- and supra-clypeal areas in female *A. preissi*. The type localities respectively, WA (*A. preissi*) and Brewarrina, NSW (*A. preissi frogatti*), may also indicate that these names belong to different species.

The description of *A. preissi frogatti* place it in the *calva* group. There is a male from the type locality: Brewarrina, NSW, 1914, WW Frogatt, in the QM collection with identification label from Hacker. The sternum morphology of this specimen shows that it is in the *calva* group, indicating that *A. frogatti* is a valid species. Although the male of *A. preissi* has never been described, the sternum morphology as well as the patch of bristles on S4, which has an elongated shape in *A. preissi* and is round in *A. frogatti*, also validates *A. frogatti* as a separate species.

#### Redescription.

Male (QM T238472): Body length 14 mm, forewing length 9.3 mm, head width 4.5 mm.

***Structure***: Inner orbits of eyes slightly diverging above; head wider than long; clypeal protuberance in profile 0.68 × eye width; mandible with distinct subapical tooth; F1 equal to combined length of next 1.6 flagellomeres; F1 0.6 × as long as scape; F2 0.58 × as long as F3; F3-F10 1.83 × as long as wide; last flagellomere 1.13 × as long as F1; marginal cell length 0.82 distance from apex of cell to wing tip; cu-v of hind wing circa as long as second abscissa of M+Cu; S5 with apicomedial emargination narrow triangular shaped circa five times as wide as deep; S6 with apicomedial emargination very shallow and wide; T7 extended emarginated resulting in two lateral teeth.

*Genitalia*: penis valves with well extended shoulders; volsella short with 12 short setae (Fig. 13H); gonocoxa laterally with a few short setae; apex of gonocoxa with acute ventral and dorsal lobes; outer gonostylus circa as long as penis valve base, with robust setae at inner surface; inner gonostylus little shorter than outer gonostylus, slightly club shaped with short robust setae at apex (Fig. 13G); S7 (Fig. 13E); S8 apex slightly emarginated, with slender setae at apex and along midline (Fig. 13F).

***Pubescence***: Head white on genae and labrum, clypeus and paraclypeal area black, vertex and around ocelli grey intermixed with black hairs; scutum with pale brown hairs anteriorly, remaining hairs grey intermixed with black hairs; scutellum and metanotum grey intermixed with black hairs; mesosoma laterally and ventrally white, under wing base grey-pale brown intermixed with black hairs; fore leg femur and tibia posteriorly with long white hair intermixed with some black hairs, pale brown hairs on outer surface; mid and hind legs black on inner surface and pale brown-grey on outer surface; metasomal terga T1 anteriorly with white hairs, on disk with pale brown hairs; T2-T5 hairs pale brown, T2 anteriorly with entire band of black hairs; T3 with entire band of black hairs, but narrower than on T2; S1-S4 with narrow fringes of adpressed white hairs on posterior margins, hairs on disks erect and pale brown darker towards sides; S4 apicomedial area with small round patch of posteriorly- and proximally-directed black bristles; S5 with long brown branched hairs around the emargination and long pale brown fringes at lateral corners; S6 with dense batch of dark brown branches hairs apicomedially.

***Colouration***: Integument black, apart from: posterior margins of sterna translucent orange-brown; legs dark brown; scape with ivory mark below, black above; labrum ivory with translucent dots near dorsolateral corners; clypeus ivory; supraclypeal area ivory; paraclypeal area ivory; mandible ivory at base brownish at tip; proboscis orange-brown.

**Female** redescription (QM T238470): Body length 14.5 mm, forewing length 10.4 mm, head width 5.2 mm.

***Structure***: Inner orbits of eyes almost parallel; head wider than long; clypeal protuberance in profile 0.69 × eye width; mandible with distinct subapical tooth; F1 equal to combined length of next 2.6 flagellomeres; F1 0.85 × as long as scape; F2 circa as long as F3; F3-F10 1.25 × as long as wide; last flagellomere 0.66 × as long as F1; marginal cell length 0.82 distance from apex of cell to wing tip; cu-v of hind wing 1.42 × as long as second abscissa of M+Cu; S6 with small median slightly raised area (Fig. 13L).

***Pubescence***: Head with white hairs on genae and labrum, clypeus and paraclypeal area black, grey intermixed with black hairs on vertex and around ocelli; scutum with anteriorly with pale brown hairs, remaining hairs grey intermixed with black hairs; scutellum and metanotum grey intermixed with black hairs; mesosoma laterally and ventrally with white pubescence and intermixed black hairs; pronotal lobes grey-pale brown, ventrally between hind coxa black; fore leg with femur and tibia posteriorly with long white hair intermixed with some black and pale brown hairs on outer surface; mid and hind legs black on inner surface and grey-white on outer surface; scopa on hind leg grey-white with streak of pale brown hair below basitibial plate; metasomal terga T1 anteriorly white intermixed with dark hairs, on disk with pale brown hairs; T2-T5 hairs pale brown; T2 anteriorly with entire band of black hairs; T3 with black hairs anterolaterally; T5 with black prepygidial fimbria; T6 with strong black hairs flanking the pygidial plate; S2-S4 on posterior margins with narrow fringes of white hairs laterally and black medially, the fringes with black hairs wider on consecutive terga, hairs on disks erect and black.

***Colouration***: Integument black, apart from: posterior margins of sterna translucent orange-brown; legs dark brown; labrum ivory with translucent dots near dorsolateral corners; clypeus ivory with two large dark brown patches in dorsolateral corners leaving a narrow ivory midline; supraclypeal area ivory; paraclypeal area ivory; mandible ivory at base brownish at tip; proboscis orange-brown.

**Table:** **Phenology.**

**Month**:	**Jan**	**Feb**	**Mar**	**Apr**	**May**	**Jun**	**Jul**	**Aug**	**Sep**	**Oct**	**Nov**	**Dec**
No. of records:	1	0	3	0	0	0	0	0	0	0	0	3

#### Flower records.

*Capparis mitchelli* (Capparaceae).

#### Distribution.

Figure 13I.

### Amegilla (Asaropoda) griseocincta

Taxon classificationAnimaliaHymenopteraApidae

Leijs, sp. nov.

7F2D3A7B-C6C8-56DB-BA55-2F62315C42F4

http://zoobank.org/844732B9-94C7-4A4D-B3CD-FB93BEB8DF32

Figure 14A-M

#### Specimens examined.

(5 males, 7 females).

#### Types.

Holotype, male, Henbury Station, Gloaming Dam, NT (24.5980S; 133.5027E), 22 May 2013, R Leijs & K Hogendoorn, on *Amyema preissi*, SAMA RL2222;

Allotype, female, Henbury Station, NT (24.4415S; 133.3363E), 18 May 2013, R Leijs & K Hogendoorn, on *Amyema preissi*, SAMA RL2214.

#### Diagnosis.

**Figure 14.**
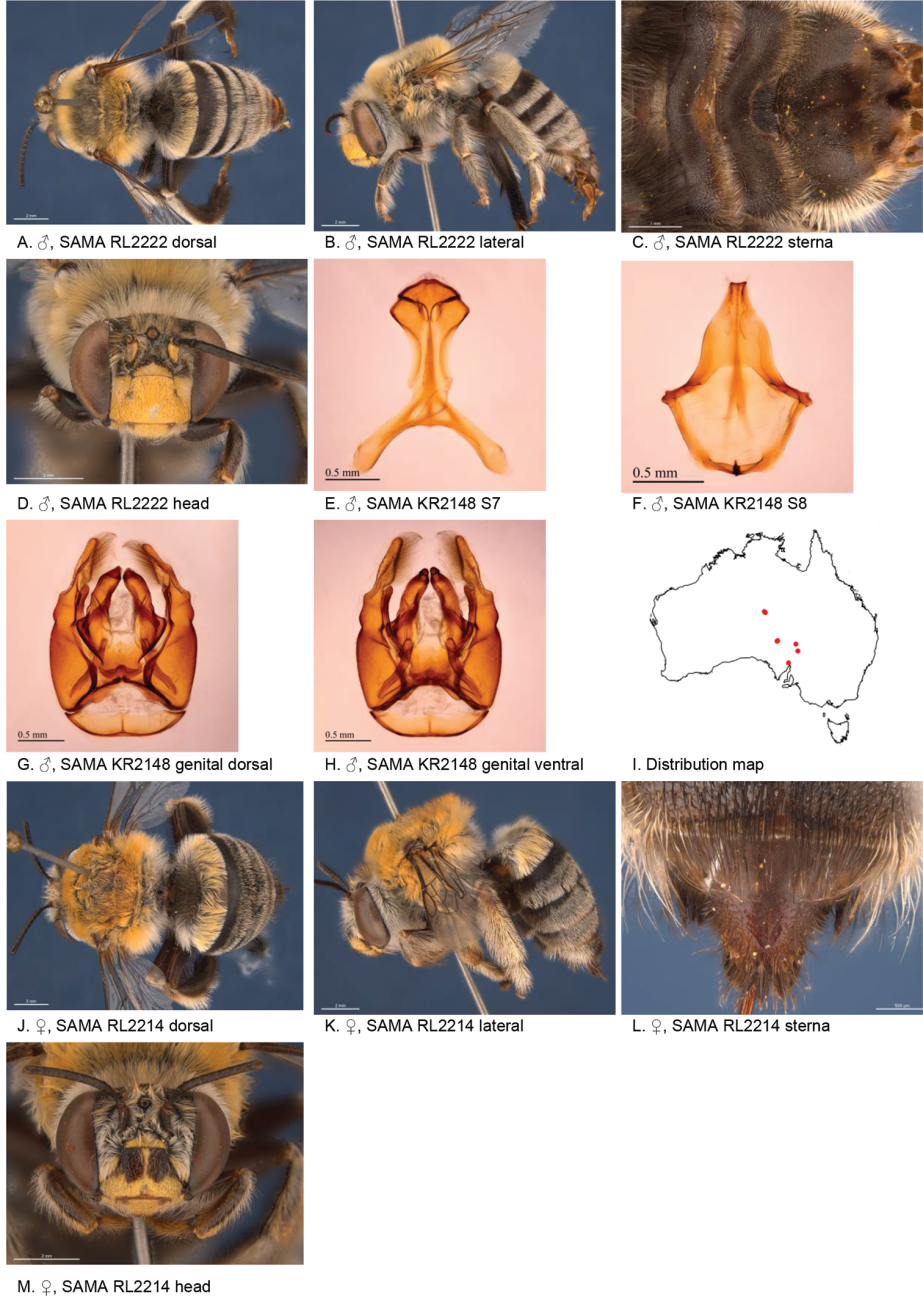
Amegilla (Asaropoda) griseocincta Leijs, sp. nov.

Male with large patch of dark brown stiff bristles on S4 and emarginated apicomedial area of S5 surrounded by branched hairs. Female paraclypeal and supraclypeal marks present, tibial scopa white, T2-T4 with black hairs anteriorly, process on S6 lineo-reticulate and well defined posteriorly (Fig. 14L).

#### Description.

Male holotype (SAMA 32-033609, RL 2222: Body length 16 mm, forewing length 10.3 mm, head width 4.9 mm.

***Structure***: Inner orbits of eyes diverging above; head wider than long; clypeal protuberance in profile 0.76 × eye width; mandible with subapical tooth; F1 equal to combined length of next 2.2 flagellomeres; F1 0.9 × as long as scape; F2 0.56 × as long as F3; F3-F10 1.33 × as long as wide; last flagellomere 0.89 × as long as F1; marginal cell length 0.81 distance from apex of cell to wing tip; cu-v of hind wing 0.74 × length of second abscissa of M+Cu; S5 with apicomedial emargination round, circa three times as wide as deep; posterior lateral corners of S5 with short spine, S6 with apicomedial emargination angular, circa four times as wide as deep; S6 medially with raised posteriorly-directed rounded ridge.

*Genitalia*: penis valves with well extended shoulders; volsella large, with 12 strong setae (Fig. 14H); gonocoxa laterally with numerous small setae rising from small depressions; apex of gonocoxa with rounded lobes dorsally and ventrally; outer gonostylus robust, slightly longer than gonocoxa base, with dense fine setae on inner surface; inner gonostylus lacking (Fig. 14G); S7 (Fig. 14E); S8 laterally slightly sigmoid, emarginate at apex, a few long setae at apex and some strong short setae at midline (Fig. 14F).

***Pubescence***: Head white with a few brown hairs at ocellocular areas and scattered dark hairs on clypeus; scutum pale brown, brown in fresh specimens; scutellum and metanotum pale brown; mesosoma laterally and ventrally pale brown under wing base and pronotal lobe, otherwise white; all legs white on outer surfaces, dark brown on inner surfaces; metasomal terga T1 anteriorly and laterally with long white hairs, on disk very pale brown, posteriorly with white adpressed hairs; T2–T4 anteriorly with brown to black hairs, remaining hairs white and adpressed; T4–6 with scattered long erect brown hairs, T7 white, with patches of dark brown hairs in posterio-lateral corners; S1-S5 white in lateral corners; S4 apicomedial area with large -circa a quarter of sterna width- patch of black anteriorly-directed bristles; S5 covered with long thin erect hairs and two large patches of posteriorly- and proximally-directed, branched brown hairs; S6 with posterior medial patch of backwards directed black branched hairs around the emargination.

***Colouration***: Integument black, apart from: posterior margins of metasomal segments translucent orange; scape yellow below; labrum yellow with small brown marks in dorsolateral corners; clypeus yellow, supraclypeal area with large triangular yellow shape; paraclypeal area yellow; mandible yellow at base, black at tip; proboscis orange.

**Female** allotype (SAMA 32-033608, RLa2214): Body length 17 mm, forewing length 10.7 mm, head width 5.6 mm.

***Structure***: Inner orbits of eyes diverging above; head wider than long; clypeal protuberance in profile 0.6 × eye width; mandible with subapical tooth [remark: mandible is worn. Specimens from Anna Creek have apparent subapical teeth], F1 equal to combined length of next 3 flagellomeres; F1 circa as long as scape; F2 0.64 × as long as F3; F3-F10 1.15 × as long as wide; last flagellomere 0.61 × as long as F1; marginal cell length 0.77 × distance from apex of cell to wing tip; cu-v of hind wing 0.87 × length of second abscissa of M+Cu; S6 with triangular area raised posteriorly (Fig. 14L).

***Pubescence***: Head white with a few brown hairs at ocellocular areas and scattered dark hairs on clypeus; scutum, scutellum and metanotum light brown; mesosoma laterally and ventrally light brown under wing base and pronotal lobe, otherwise white; fore leg femur and tibia with long plumes of white hairs; outer surface of tarsi brown, inner surface orange; mid and hind legs white on outer surfaces, black on inner surfaces; metasomal terga T1 anteriorly and laterally with long white hairs, on disk pale brown, posteriorly with white adpressed hairs; T2–T3 anteriorly with brown to black hairs, remaining hairs white and adpressed and scattered long erect brown hairs on T3–T5, T5 apically with dense band of stiff black hairs, T6 near pygidial plate with stiff orange hairs laterally and dark brown apically; S1-S5 with rows of long erect hairs on premarginal lines, dark medially and white laterally.

***Colouration***: Integument black, apart from: posterior margins of metasomal segments translucent orange; legs brown; labrum yellow with small brown marks in dorsolateral corners; clypeus with yellow inverted T-shape with broad base; supraclypeal area with triangular yellow shape; paraclypeal area partly yellow; mandible yellow at base, black at tip; proboscis orange.

**Table:** **Phenology.**

**Month**:	**Jan**	**Feb**	**Mar**	**Apr**	**May**	**Jun**	**Jul**	**Aug**	**Sep**	**Oct**	**Nov**	**Dec**
No. of records:	0	0	1	8	3	0	0	0	0	0	0	0

#### Flower records.

*Amyema preissi* (Loranthaceae), *Eremophila* (Scrophulariaceae).

#### Distribution.

Figure 14I.

#### Etymology.

The specific epithet refers to the greyish hair bands on the metasomal terga.

### Amegilla (Asaropoda) houstoni

Taxon classificationAnimaliaHymenopteraApidae

Brooks, 1988

43FDBD43-66C6-5A2C-826E-1704DFE2A216

Figure 15A-E

Amegilla (Asaropoda) houstoni Brooks, 1988: 555.

#### Specimens examined

(1 female).

#### Types.

Holotype of *A. houstoni*: female, Top of Napier Range, Windjana Gorge, Kimberley Div. W. Aust., 10 Apr. 1980, G Anderson & D Symon, on *Trichodesma*, WAM 87/1303, Reg no. 22605.

#### Remarks.

The description of the female holotype of this species can be found in Brooks (1988).

#### Diagnosis.

**Figure 15.**
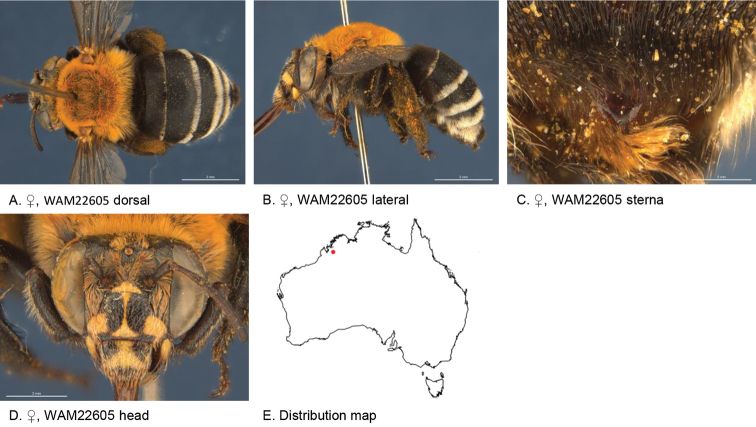
A-E. Amegilla (Asaropoda) houstoni Brooks, 1988.

Metasomal terga with black pubescence and white posterior hair bands, narrow on T1 (Fig. 15A, 15B). Male unknown.

**Table:** **Phenology.**

Month:	Jan	Feb	Mar	Apr	May	Jun	Jul	Aug	Sep	Oct	Nov	Dec
No. of records:	0	0	0	1	0	0	0	0	0	0	0	0

#### Flower records.

*Trichodesma* (Boraginaceae).

#### Distribution.

Figure 15E.

### Amegilla (Asaropoda) incognita

Taxon classificationAnimaliaHymenopteraApidae

Leijs, sp. nov.

CC1E023C-42C6-52B0-A6CF-6EC6C068C387

http://zoobank.org/1AD70374-ABAD-4A9D-8C1B-290C59D2AC68

Figure 16A–I

#### Specimens examined

(1 male).

#### Types.

Holotype, male, 55 mls South of Onslow, WA (22.4357S; 115.1119E), 23 Aug. 1971, TF Houston, on *Trichodesma*, WAM 26816.

#### Diagnosis.

Male with large round patch of dark brown stiff bristles on S4 and emarginated apicomedial area of S5. T2-T4 anteriorly with black hairs. Female unknown.

#### Description.

Male (WAM 26816): Body length 14 mm, forewing length 9.2 mm, head width 4.4 mm.

***Structure***: Inner orbits of eyes slightly diverging above; head wider than long; clypeal protuberance in profile 0.64 × eye width; mandible with subapical tooth; F1 equal to combined length of next 1.7 flagellomeres, as long as F3; F1 0.60 × as long as scape; F2 0.63 × as long as F3; F3-F10 circa 1.3 × as long as wide; last flagellomere as long as F1; marginal cell length 0.73 × distance from apex of cell to wing tip; cu-v of hind wing 1.14 × length of second abscissa of M+Cu; S5 with apicomedial emargination 4.5 × as wide as deep, S6 with apicomedial emargination 4.6 × as wide as deep.

**Figure 16.**
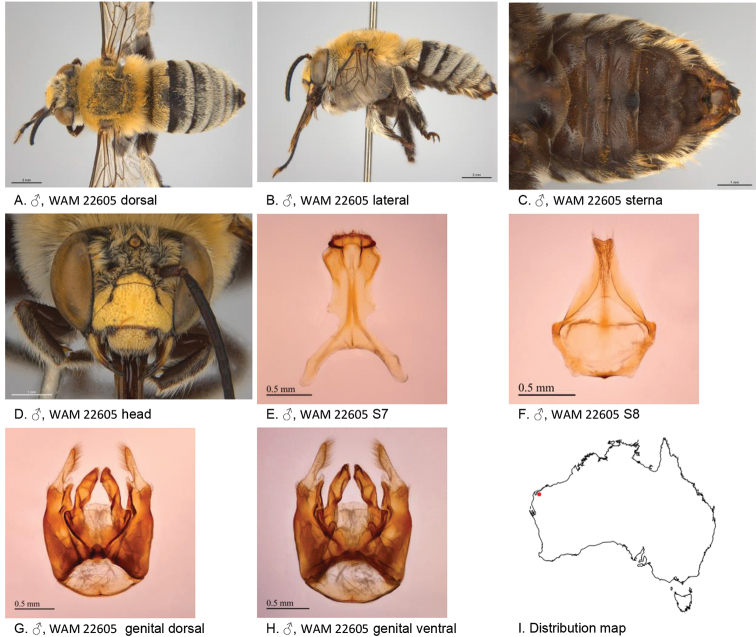
Amegilla (Asaropoda) incognita Leijs, sp. nov.

*Genitalia*: penis valves laterally with extended rounded shoulders; volsella with circa 10 long setae (Fig. 16H); gonocoxa laterally with setae lacking; apex of gonocoxa ventrally with rounded process (Fig. 16H); outer gonostylus long, circa as long as width of gonocoxa base, with long setae on inner surface; inner gonostylus small, circa the size of process on the gonocoxa apex; S7 (Fig. 16E); S8 apex emarginated, strong and dense setae along the midline (Fig. 16F).

***Pubescence***: Head white on face, vertex and genae, some dark hairs below lateral ocelli; scutum, scutellum and metanotum light brown with a few longer dark hairs; mesosoma laterally and ventrally pale to white; fore leg white on outer surface, inner surface of tibia and tarsus with short dark brown to black hair; mid femur dorsally almost bare, ventrally brown, apically with a few white hairs, mid tibia outer surface white, inner surface almost bare, mid metatarsus outer surface white, inner surface with brown to black hairs, mid tarsus remaining segments with black hairs; hind leg outer surface of femur, tibia and metatarsus white; metatarsus with white streak, hind leg inner surface black; metasomal terga: T1 with light brown erect branched hairs; T2 anteriorly with adpressed black hairs, forming a black band; T3-T6 with adpressed white hairs, some black hairs on anterolateral corners; T7 covered in black hairs; S1-S5 with laterally with a few white hairs, fringes of dark hairs on marginal zones; S3 apicomedial area with tiny streak of black setae; S4 apicomedial area with round patch of robust black apically-directed bristles and long white hairs on lateral margins; S5 with long dark branched hairs around the apico-median emargination and long white hairs on lateral margins; S6 with streaks of black hairs on the lateral margins, short black hairs on the disc and dense patch of hairs covering the marginal zone.

***Colouration***: Integument mostly black; sterna brown; scape brown below; labrum pale yellow with brown dots in dorsolateral corners; clypeus, supraclypeal area and paraclypeal area pale yellow; mandible basal half pale yellow, remaining part brown and black; proboscis orange-brown.

**Table:** **Phenology.**

**Month**:	**Jan**	**Feb**	**Mar**	**Apr**	**May**	**Jun**	**Jul**	**Aug**	**Sep**	**Oct**	**Nov**	**Dec**
No. of records:	0	0	0	0	0	0	0	1	0	0	0	0

#### Remarks.

This single male specimen from Onslow, WA in the WA Museum collection has an identification label by Brooks with identification ‘*A. preissi*’. This is puzzling, because a description of the male of *A. preissi* did not exist at the time, and the specimen was not associated with a female because series of females and males of this species with same collecting data do not exist. While the male sternum and morphology of the genitalia the clearly show that the specimen belongs to the *calva* group these and other characters are sufficiently different from the other species in that group to justify description of an additional species.

#### Flower records.

*Trichodesma* (Boraginaceae).

#### Distribution.

Figure 16I.

#### Etymology.

The specific epithet refers to: incognita meaning ‘unknown’ or unrecognised.

### Amegilla (Asaropoda) nitidiventris

Taxon classificationAnimaliaHymenopteraApidae

Leijs, sp. nov.

0AD98D38-0A2C-5FA9-85CC-F1496D3EEFF4

http://zoobank.org/1EBFCC51-2888-465B-8CB5-D9830B373BDB

Figure 17A-M

#### Specimens examined.

(9 males, 6 females):

#### Types.

Holotype male, Laura Roadhouse, Qld (15.5657S; 144.4488E), 10 Mar. 2017. R Leijs, QM T238477;

Allotype, female, same locality data as holotype, QM T238478;

Paratypes, 1 male, same locality data as holotype, SAMA KR06079; 1 female, Laura Roadhouse, Qld (15.5657S; 144.4461E), 6 Mar. 2017, R Leijs, on *Hibiscus*, SAMA KR06006; 1 male, 6.7km NE of Laura, Qld, (15.5011S; 144.4838E), R Leijs, from blue vane trap, SAMA KR06027; 2 males, Laura General Store, Qld (15.5583S; 144.4461E), 11 Mar. 2017, R Leijs, on *Turnera subulata*, SAMA KR06084, KR06092; 3 males, 3 females, Laura General Store, Qld (15.5583S; 144.4461E), 15 Mar. 2017, R Leijs, on *Turnera subulata*, SAMA KR06191-96.

#### Diagnosis.

**Figure 17.**
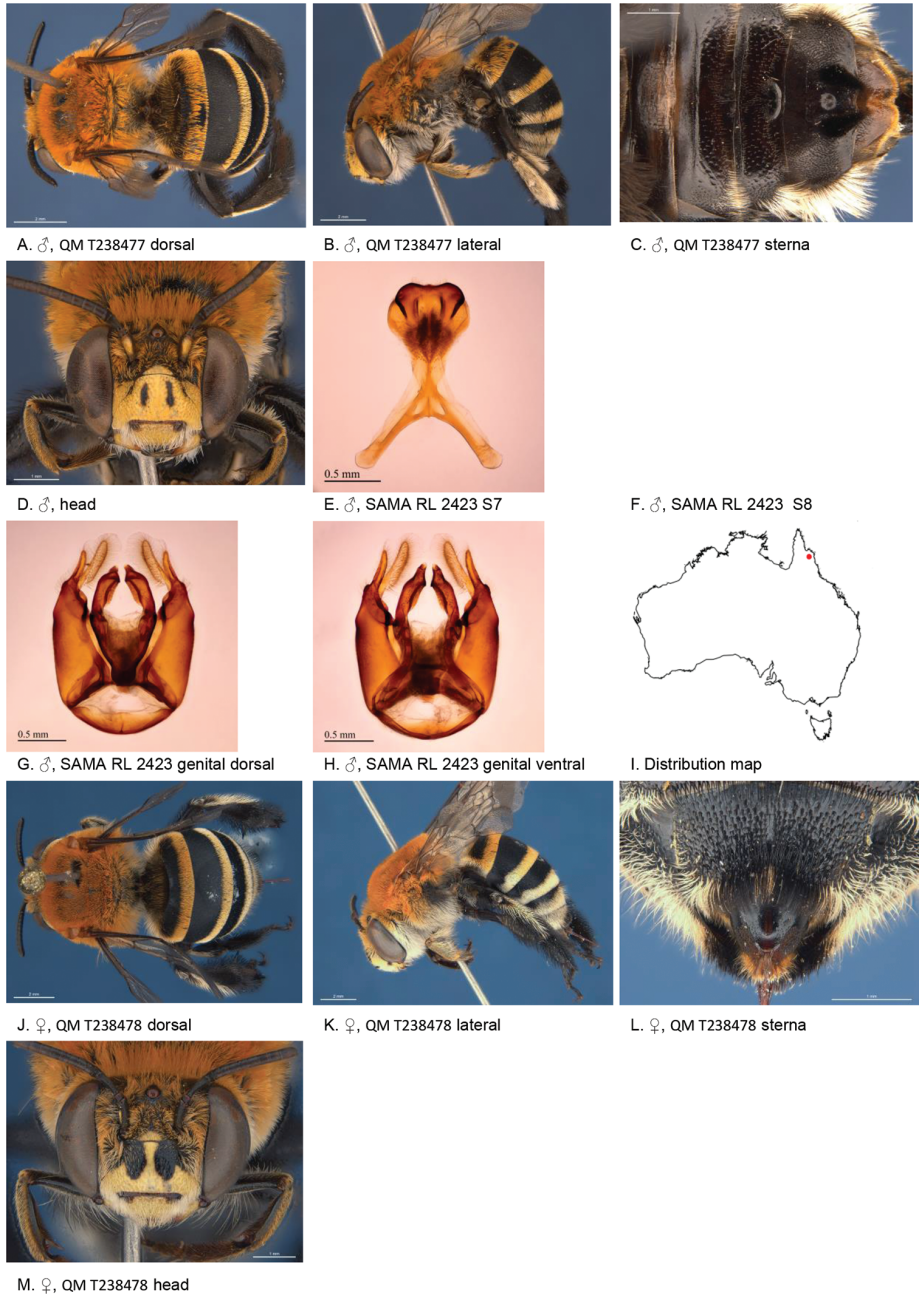
Amegilla (Asaropoda) nitidiventris Leijs, sp. nov.

Both sexes metasomal terga with black pubescence and light posterior hair bands, sterna black and shiny. Male with two small adjacent patches of black bristles on S4 and two large comma-shaped patches of bristles on S5. Female paraclypeal marks absent and supraclypeal marks present, tibial scopa black with white dorsal streak, process on S6 well defined with smooth posteriorly protruding lobe (Fig. 17L).

#### Description.

Male holotype (QM T238477, RLa2427): Body length 12 mm, forewing length 9.2 mm, head width 4.3 mm.

***Structure***: Inner orbits of eyes diverging above; head wider than long; clypeal protuberance in profile 0.64 × eye width; mandible without subapical tooth; F1 equal to combined length of next 1.8 flagellomeres; F1 0.59 × as long as scape; F2 0.58 × as long as F3; F3-F10 1.17 × as long as wide; last flagellomere 1.24 × as long as F1; marginal cell length 0.78 × distance from apex of cell to wing tip; cu-v of hind wing 0.81 × length of second abscissa of M+Cu; S5 with apicomedial emargination vary shallow, and a single knob medially; S6 with apicomedial emargination very shallow.

*Genitalia*: penis valves shoulders not extended, base narrow; volsella small, with 6 long setae (Fig. 17H); gonocoxa laterally with numerous small setae; apex of gonocoxa broad, ventrally without process, but with rounded notch near volsella (Fig. 17H); outer gonostylus slender, circa 0.6 × as long as inner gonostylus, with setae on inner surface; inner gonostylus robust, with numerous strong setae (Fig. 17G); S7 (Fig. 17E).

***Pubescence***: Head white on labrum, clypeus, scape and lower half of genae; pale orange on upper genae and frons above antennal sockets; scutum, scutellum and metanotum orange-brown; mesosoma laterally and ventrally orange-brown under wing basis and on pronotal lobe, white ventrally, distinct patch of black hair below hind wing; fore leg with long white hairs on posterior parts of femur, outer surface of tibia and metatarsus with greyish hairs; fore leg inner surfaces of tibia and tarsus with short white to brown hairs; mid and hind legs pubescence black apart from long white hairs on trochanters and outer surface of tibia; metasomal terga T1 pale with orange hairs anteriorly, narrow band of black hairs on disk and orange posterior adpressed hair band; T2–T4 with short adpressed black hairs on disk, posterior adpressed hairband on T2 pale orange, posterior hairband on T3 white with pale orange tint, T4 with white posterior hairband extended to disk and scattered erect black and white hairs across whole segment; T5 white hairband broad and extended to disk; T6 white; S1-S5 with black hairs and patches of white hairs at lateral corners; S4 apicomedial area with small triangular patch of ventrally directed black bristles, S5 with two longitudinal semi parallel patches of posteriorly- and proximally-directed black bristles and long fringes of white hairs at lateral corners.

***Colouration***: Integument black, apart from: scape brown-black yellow patch below; labrum pale yellow with brown marks in dorsolateral corners; clypeus pale yellow, with two small black parallel lines in the centre; supraclypeal area with pale yellow triangle; paraclypeal area pale yellow; mandible pale yellow at base, brown at tip; proboscis brown.

**Female** allotype (QM T238478, RL a2422): Body length 15 mm, forewing length 10.3 mm, head width 4.8 mm.

***Structure***: Inner orbits of eyes diverging above; head wider than long; clypeal protuberance in profile 0.59 eye width; mandible without subapical tooth; F1 equal to combined length of next 3.3 flagellomeres; F1 almost as long as scape (0.96 times), F2 0.88 × as long as F3; F3-F10 circa as long as wide; last flagellomere 0.72 × as long as F1; marginal cell length 0.80 × distance from apex of cell to wing tip; cu-v of hind wing 0.77 × length of second abscissa of M+Cu; S6 with distinctly rounded posteriorly projecting subapical lobe, visible as spine when viewed laterally (Fig. 17L).

***Pubescence***: Head white on labrum, clypeus, scape and lower half of genae; pale orange hairs on upper genae and frons above antennal sockets; scutum, scutellum and metanotum orange-brown; mesosoma laterally and ventrally with orange-brown hairs under wing basis and on pronotal lobe, white ventrally, distinct patch of black hair below hind wing; fore leg with long white hairs on posterior parts of femur, outer surface of tibia and metatarsus with adpressed white hairs, and metatarsus with long black hairs; fore leg inner surfaces of tibia and tarsus with short brown hairs, mid and hind legs pubescence black apart from long white hairs on trochanters, very short adpressed hairs ventrally on femur, outer surface of mid tibia, and white streak on tibial scopa basally; metasomal terga T1 pale with orange hairs anteriorly, band of black hairs on disk, posteriorly with orange adpressed hair band, long white hairs on lateral corners; T2–T4 with short adpressed black hairs on disk, posterior pale orange adpressed hairband on T2, posterior hairband on T3 white, few strong erect hairs at premarginal line black medially and white laterally, T4 posterior hairband extended to disk, scattered erect black and white hairs across whole segment, T5 with white adpressed hairs and dense patch of black hairs apicomedially, T6 with strong black hairs flanking the pygidial plate; S1-S5 with black hairs and patches of white hairs at lateral corners.

***Colouration***: Integument black, apart from: labrum ivory with brown marks in dorsolateral corners, clypeus ivory, with two large black ovoid marks in dorsolateral corners, supraclypeal area with ivory triangle, paraclypeal area black, mandible ivory at base, brown at tip, proboscis brown.

**Table:** **Phenology.**

**Month**:	**Jan**	**Feb**	**Mar**	**Apr**	**May**	**Jun**	**Jul**	**Aug**	**Sep**	**Oct**	**Nov**	**Dec**
No. of records:	0	0	15	0	0	0	0	0	0	0	0	0

#### Flower records.

*Hibiscus* (Malvaceae), *Turnera subulata* (Passifloraceae)

#### Distribution.

Figure 17I.

#### Etymology.

The specific epithet refers to shiny black metasomal sterna in both sexes.

### Amegilla (Asaropoda) preissi

Taxon classificationAnimaliaHymenopteraApidae

(Cockerell, 1910)

C1E40EEE-7969-5EBA-95C1-61C90CE207E4

Figure 18A–M

Anthophora preissi Cockerell, 1910: 107.
Asaropoda sordidula Rayment, 1931: 179, **syn. nov.**
Asaropoda sordida Rayment, 1931: 180, **syn. nov.**
Asaropoda grisescens Rayment, 1931: 181, **syn. nov.**

#### Specimens examined.

45 males, 57 females.

#### Types.

Holotype of *A. preissi*, female, N.H. occ. Preiss (as New Holland, occidental = Western Australia), 1405, ZMB.

Holotype of *A. sordidula*, male, Swan River, WA, L. J. Newman, Agriculture (Dept) Western Australia 28866.

Holotype of *A. grisescens*: female, Geraldton, W. Australia, ANIC.

Holotype of *A. sordida*: male, Geraldton, W. Australia, L. J. Newman, ANIC.

#### Decision for synonymy.

Examination and comparison of the female types of *A. preissi* and *A. grisescens* did not reveal morphological differences with respect of female diagnostic characters, therefore the name *A. preissi* has precedence. The sexes of this species were associated using a series of specimens of both sexes with identical locality data in the WAM and VM collections linking females of *A. grisescens* with males of *A. sordida* and *A. sordidula*. The emargination and hair patches of the sterna of the male type of *sordida* are not different from those of the male type of *A. sordidula*.

#### Diagnosis.

**Figure 18.**
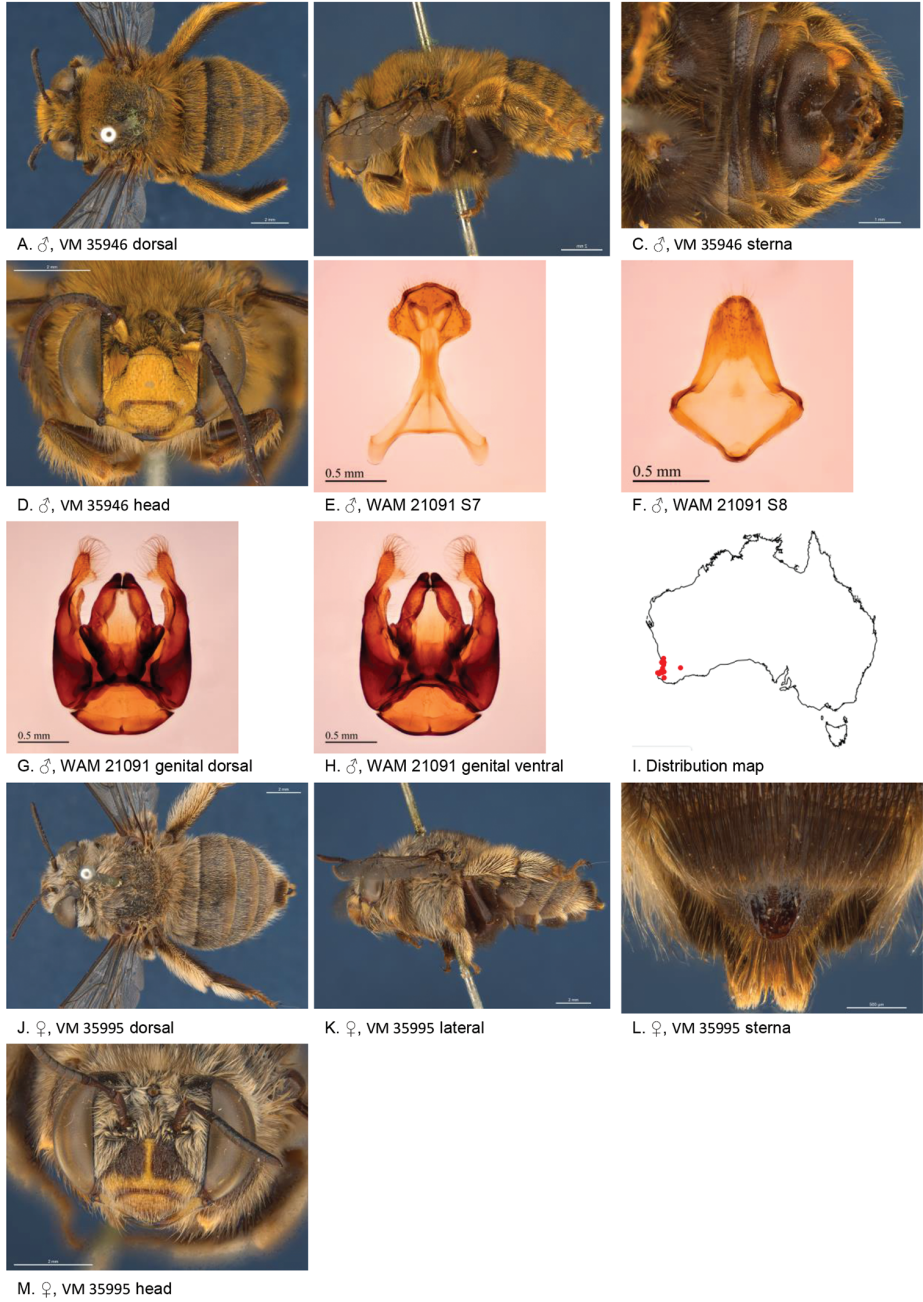
Amegilla (Asaropoda) preissi (Cockerell, 1910).

Male with rectangular patch of dark and light brown bristles on S4 and angular emarginated apicomedial area of S5 surrounded by branched hairs. Female paraclypeal and supraclypeal marks absent, tibial scopa grey-white, T2 with narrow band of black hairs anteriorly, process on S6 smooth and well defined posteriorly (Fig. 18L).

#### Redescription.

Male (VM HYM-35946): Body length 13.5 mm, forewing length 9.7 mm, head width 5.1 mm.

***Structure***: Inner orbits of eyes almost parallel; head wider than long; clypeal protuberance in profile 0.69 × eye width; mandible with distinct subapical tooth; F1 equal to combined length of next 2.2 flagellomeres; F1 0.63 × as long as scape; F2 0.62 × as long as F3; F3-F10 1.27 × as long as wide; last flagellomere 0.78 × as long as F1; marginal cell length 0.75 distance from apex of cell to wing tip; cu-v of hind wing 0.83 × as long as second abscissa of M+Cu; S5 with apicomedial emargination triangular shaped circa 2.6 × as wide as deep; posterior edge of S5 undulated; S6 with apicomedial emargination circa 3.6 × as wide as deep; marginal zone broad and smooth.

*Genitalia*: penis valves with well extended shoulders; volsella slender with 5 setae (Fig. 18H); gonocoxa laterally with some slender setae raising from depressions; apex of gonocoxa with rounded ventral lobe; outer gonostylus circa as long as penis valve base, with long setae at inner surface and apex; inner gonostylus 0.6 as long as outer gonostylus, slightly rectangular shaped with long setae at apex (Fig. 18G); S7 (Fig. 18E); S8 broadly rounded (Fig. 18F).

***Pubescence***: Head white-grey on genae and labrum, pale brown intermixed with black hairs on clypeus, paraclypeal area vertex and area around ocelli; scutum, scutellum and metanotum pale brown intermixed with black hairs; mesosoma laterally pale brown, black ventrally; fore leg pale brown; mid and hind legs black on inner surface and pale brown on outer surface; metasomal terga T1 with long pale brown erect hairs; T2–T6 with semi adpressed pale brown hairs; T7 hairs brown; T2 anteriorly with entire narrow band of black hairs; S1–S4 with narrow fringes of adpressed short white branched hairs laterally on posterior margins and single rows of erect black hairs near pre-marginal line, hairs on disks erect and black; S4–S5 laterally with plumes of pale brown hairs; S4 apicomedial area with two ovoid patches of anteriomedial directed black bristles, together circa one third of sternum width; S5 with long brown branched hairs on both sides of the emargination; S6 without hairs on marginal zone.

***Colouration***: Integument dark-brown, apart from: posterior margins of sterna translucent orange-brown; scape yellow below black above; flagellum brown below; labrum yellow with translucent dots near dorsolateral corners; clypeus, supraclypeal and paraclypeal area yellow; mandible yellow at base dark brown at tip; proboscis orange-brown.

**Female** redescription (VM HYM-35995): Body length 15 mm, forewing length 10.3 mm, head width 5.5 mm.

***Structure***: Inner orbits of eyes slightly diverging above; head wider than long; clypeal protuberance in profile 0.8 × eye width; mandible with distinct subapical tooth; F1 equal to combined length of almost next three flagellomeres; F1 0.83 × as long as scape; F2 0.83 × as long as F3; F3-F10 circa as long as wide; last flagellomere 0.57 × as long as F1; marginal cell length 0.78 distance from apex of cell to wing tip; cu-v of hind wing 0.8 × as long as second abscissa of M+Cu; S6 with broad parabolically raised smooth area (Fig. 18L).

***Pubescence***: Head grey-white on genae, labrum, clypeus, paraclypeal, vertex and around ocelli, intermixed with black hairs on clypeus, around ocelli and vertex; scutum, scutellum and metanotum grey-white intermixed with black hairs; mesosoma laterally grey-white, black hairs ventrally; fore leg with femur and tibia posteriorly with long brown and white hairs; anteriorly with short adpressed white hairs; tarsi pale brown; mid and hind legs black on inner surface and grey-white on outer surface; scopa on hind leg grey-white with streak of pale brown hair below basitibial plate, metasomal terga with grey-white hairs; T2 anteriorly without adpressed black hairs; T5 with brown prepygidial fimbria; T6 with strong pale brown hairs flanking the pygidial plate; S1-S3 with fringes of adpressed white branched hairs laterally on posterior margins and single rows of erect simple black hairs near pre-marginal line, hairs on disks erect and black; S4-S5 on posterior margins with dense rows of erect brown branched hairs.

***Colouration***: Integument of head, mesosoma and metasomal terga dark-brown with pale brown translucent posterior margins; sterna and legs brown; scape brown; flagellum brown below and darker above; labrum pale yellow with translucent dots near dorsolateral corners; clypeus with yellow inverted T-shape, otherwise brown; supraclypeal area black without yellow mark; paraclypeal area black without yellow mark; mandible yellow at base yellowish brown at tip; proboscis orange-brown.

**Table:** **Phenology.**

**Month**:	**Jan**	**Feb**	**Mar**	**Apr**	**May**	**Jun**	**Jul**	**Aug**	**Sep**	**Oct**	**Nov**	**Dec**
No. of records:	3	54	27	0	0	0	0	0	0	0	0	0

#### Remarks.

When Brooks (1993) described *A. paracalva* he mentioned that *A. calva* and *A. preissi* are restricted to New South Wales and Queensland. The ‘*A. preissi* ‘referred to by Brooks (1993) actually was *A. frogatti*, a species from south-east Queensland, that he had synonymised with *A. preissi* without giving a reason for doing so. *Amegilla preissi* as we now know it is restricted to south-west Western Australia.

#### Flower records.

*Eucalyptus calophylla* (Myrtaceae).

#### Distribution.

Figure 18I.

### Amegilla (Asaropoda) rhodoscymna

Taxon classificationAnimaliaHymenopteraApidae

(Cockerell, 1905)

AAB833A6-4C6D-558C-B4E0-EADC1FE90EDA

Figure 19A–M

Asaropoda rhodoscymna Cockerell, 1905: 395.
Anthophora rufescens Friese, 1911: 449, **syn. nov.**

#### Specimens examined

(32 males, 22 females).

#### Types.

Syntypes of *A. rhodoscymna*, 3 males, Mackay district QLD, NHMUK.

Holotype of *A. rufescens*, male, Mackay, Queensland, March 1899, G. Turner leg., ZBM.

#### Decision for synonymy.

Examination of the male holotype of *Anthophora rufescens* (Berlin Museum) and the original description of *An. rufescens*, particularly regarding the colour of the integument and the morphology of the male sterna supports the synonymisation with *A. rhodoscymna*.

#### Diagnosis.

**Figure 19.**
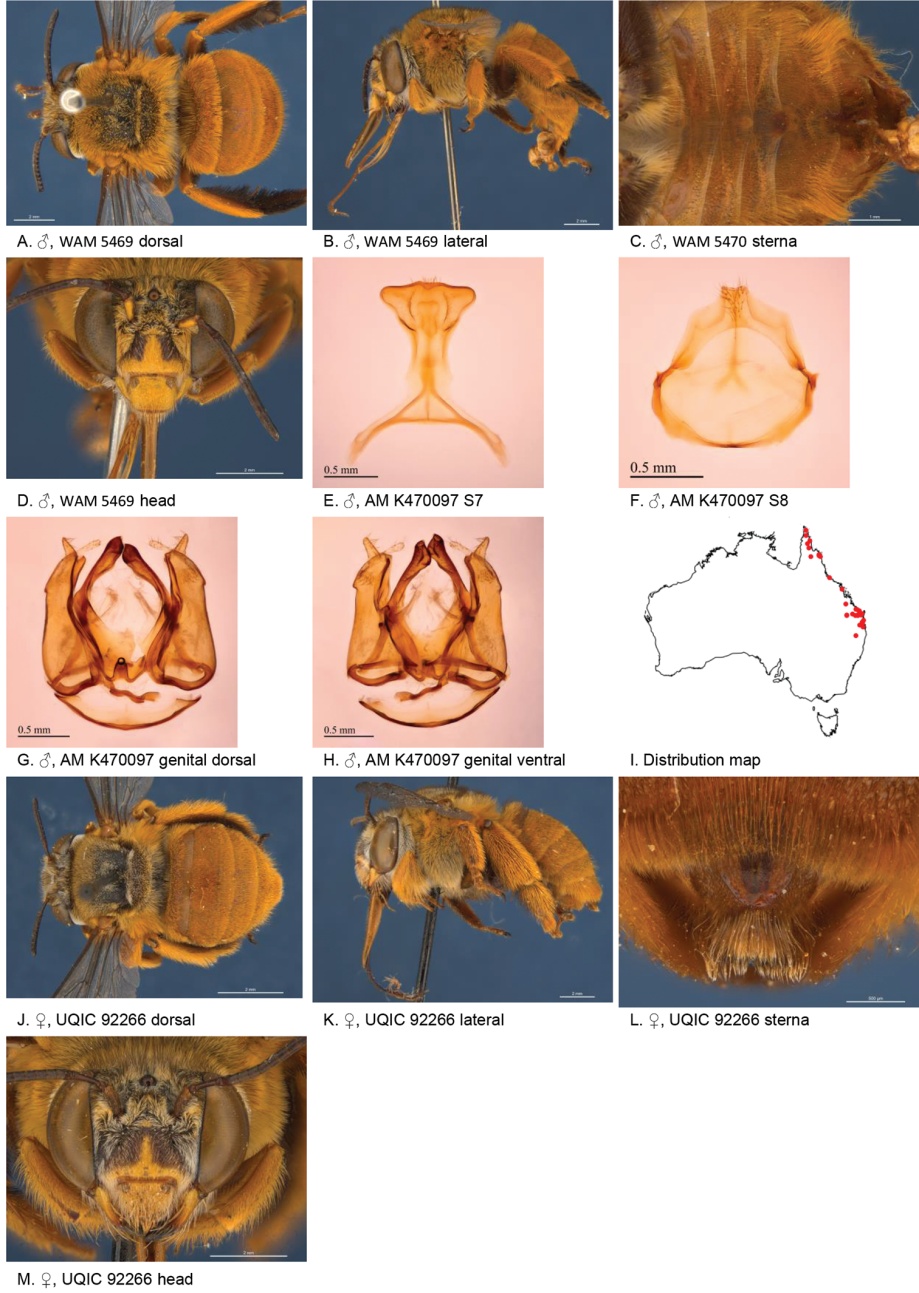
Amegilla (Asaropoda) rhodoscymna (Cockerell, 1905).

Both sexes metasomal integument reddish. Male with small round patch of orange bristles on S4, a much smaller patch on S3 and a deep (circa half the width of the segment) emargination on apicomedial area of S5. Female paraclypeal marks absent and supraclypeal marks small, tibial scopa orange, terga lacking black hairs anteriorly, process on S6 well defined broad parabolic and lineo-reticulate (Fig. 19L).

#### Redescription.

Male (WAM 5469): Body length 13 mm, forewing length 10.4 mm, head width 5 mm.

***Structure***: Inner orbits of eyes diverging above; head wider than long; clypeal protuberance in profile 0.5 × eye width; mandible with subapical tooth; F1 equal to combined length of next 2 flagellomeres; F1 0.79 × as long as scape; F2 0.67 × as long as F3; F3-F10 1.38 × as long as wide; last flagellomere 0.88 × as long as F1; marginal cell length 0.85 distance from apex of cell to wing tip; cu-v of hind wing 0.8 × as long as second abscissa of M+Cu; S5 with apicomedial emargination deep and parabolic shaped (Fig. 19C), 1.3 × as wide as deep; S6 with apicomedial emargination very shallow, preceded by smooth median area.

*Genitalia*: penis valves slender, laterally rounded without clear shoulders; volsella slightly rectangular with three long setae (Fig. 19H); gonocoxa laterally with a few short setae; apex of gonocoxa broadened with hardly extended dorsal and ventral lobes; outer gonostylus small and narrow, slightly laterally directed with few strong short setae; inner gonostylus small, inwards with a few strong and short setae (Fig. 19G); S7 (Fig. 19E); S8 short and wide, apex emarginated, some short robust setae along midline (Fig. 19F).

***Pubescence***: Head white apart from pale yellow intermixed with black hairs on vertex and around ocelli, and some long black hairs on clypeus laterally and in paraclypeal area; scutum, scutellum and metanotum orange intermixed with black hairs, some white hairs mid dorsally and in fissure between scutum and scutellum; propodeum white; mesosoma laterally and ventrally orange, paler towards ventral side, white ventrally; fore leg with pale orange hairs; mid femur, mid and hind tibia and tarsus pale orange on outer surfaces; femur, and tibia and tarsus on inner surfaces black-brown; metasomal terga with orange hairs, T1 intermixed with black hairs, T2-T5 with adpressed hairs with metallic shine; T2 anteriorly without black hairs; S1-S5 with orange hairs; S4 apicomedial area with small round patch of posteriorly directed orange-brown bristles; a smaller patch of orange, posteriorly directed bristles on S3; S5 with simple brown hairs around the emargination, S6 with simple orange hairs flanking the median smooth area.

***Colouration***: Integument of head black, apart from: scape brown above, yellow below; flagellum brown; labrum yellow with translucent dots near dorsolateral corners; clypeus yellow with two large brown patches beside the midline, making the yellow inverted T-shape; supraclypeal area with narrow triangular yellow mark; paraclypeal area yellow; mandible yellow at base, black at tip; proboscis orange-brown; mesosoma black; metasomal terga light brown; sterna and legs orange, hind coxa and femur brown.

**Female** redescription (UQIC 92266): Body length 14 mm, forewing length 11.2 mm, head width 5.6 mm.

***Structure***: Inner orbits of eyes diverging above; head wider than long; clypeal protuberance in profile 0.65 × eye width; mandible with subapical tooth; F1 equal to combined length of next 3.3 flagellomeres; F1 circa as long as scape; F2 slightly shorter than F3; F3-F10 slightly longer than wide; last flagellomere 0.58 × as long as F1; marginal cell length 0.77 distance from apex of cell to wing tip; cu-v of hind wing 0.8 × as long as second abscissa of M+Cu; S6 with broad parabolically raised area (Fig. 19L).

***Pubescence***: Head white on genae, labrum, clypeus, paraclypeal area and frons, pale yellow intermixed with black hairs on vertex and around ocelli, some long black hairs on clypeus laterally and in paraclypeal area; scutum with pale orange to grey and intermixed black hairs, white hairs mid dorsal and in fissure between scutum and scutellum; scutellum and metanotum grey intermixed black hairs, propodeum white; mesosoma laterally and ventrally with orange hairs, paler towards ventral side, white ventrally; fore leg with pale orange hairs; mid and hind tibia and tarsus orange on outer surfaces, femur and inner surface of mid leg brown, hind leg black; metasomal terga orange, intermixed with black hairs on T1; T2-T5 adpressed hairs with metallic shine; T2 anteriorly without black hairs; T5 with orange prepygidial fimbria; T6 with strong orange-brown hairs flanking the pygidial plate; S1 with narrow fringe of adpressed white hairs on posterior margin; S2-S4 with black erect hairs; S5 with dense band of branched light brown hairs on posterior margin.

***Colouration***: Integument of head black apart from: scape brown, flagellum brown; labrum yellow with translucent dots near dorsolateral corners; clypeus brown with yellow inverted T-shaped mark; supraclypeal area with small triangular yellow mark; paraclypeal area black; mandible yellow at base, black at tip; galea orange, remaining parts brown; mesosoma black; metasomal terga light brown; sterna and legs orange, hind coxa and femur brown.

**Table:** **Phenology.**

**Month**:	**Jan**	**Feb**	**Mar**	**Apr**	**May**	**Jun**	**Jul**	**Aug**	**Sep**	**Oct**	**Nov**	**Dec**
No. of records:	7	7	5	2	1	3	1	2	0	2	4	10

#### Flower records.

*Angophora costata* (Myrtaceae), *Xanthorrhoea* (Xanthorrhoeaceae).

#### Distribution.

Figure 19I.

### Amegilla (Asaropoda) scoparia

Taxon classificationAnimaliaHymenopteraApidae

Leijs, sp. nov.

BFCED0B6-1996-5789-AD66-16F502240C89

http://zoobank.org/70ECA1D2-E6BE-4AC8-80A8-10263D49FAB9

Figure 20A-M

#### Specimens examined.

(45 males, 66 females).

#### Types.

Holotype, male, Beltana Station, SA (30.6179S: 139.3214E), 15 Jun. 2009, R & P Leijs, on *Eremophila*, SAMA 32-033613, RL1452.

Allotype, female, Andamooka Homestead, SA (30.7263S; 137.2015E), 29 Aug. 2016, R Leijs, on *Eremophila*, SAMA 32-033614, KR04604.

Paratypes, 3 females, Port Augusta Arid Lands Botanic Garden, SA (32.4630S; 137.7433S), 25 Sep. 2014, R Leijs, on *Eremophila*, SAMA KR01290-2; 1 female, Great Victoria Desert, SA (29.00673S; 130.2604E), 26/08/2015, R. Leijs, on *Senna pleurocarpa*, SAMA KR02653; 1 male, Great Victoria Desert, SA (29.3329S; 130.1971E), 26 Aug. 2015, R Leijs, from vehicle net, SAMA KR02652; 3 females, Great Victoria Desert, SA (28.9503S; 130.1445E), 01 Sep. 2015, R Leijs, on *Senna pleurocarpa*, SAMA KR02971-3; 2 males, 2 females, SAMA KR04601-3,5, same locality data as for allotype.

#### Diagnosis.

**Figure 20.**
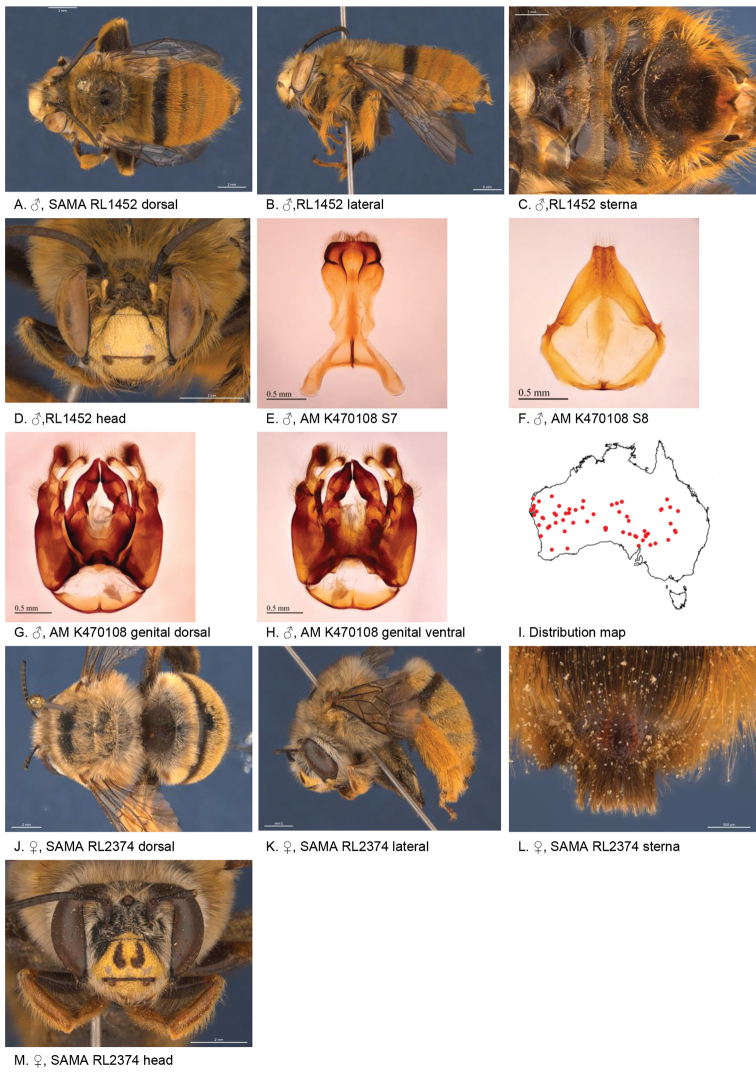
Amegilla (Asaropoda) scoparia Leijs, sp. nov.

Male with wide rectangular patch of brown bristles on S4 and widely emarginated apicomedial area of S5, apex of S6 with conspicuous two-coloured y-shaped hair brush. Female paraclypeal marks absent, supraclypeal marks present, tibial scopa pale orange, T2 with band of black hairs anteriorly, process on S6 large transverse lineo-reticulate, not well defined posteriorly (Fig. 20L).

#### Description.

Male (SAMA 32-033613, RL1452): Body length 15 mm, forewing length 11 mm, head width 4.9 mm.

***Structure***: Inner orbits of eyes slightly diverging above; head wider than long; clypeal protuberance in profile 0.93 × eye width; mandible with weak subapical tooth; F1 equal to combined length of next two flagellomeres; F1 0.93 × as long as scape; F2 0.68 × as long as F3; F3-F10 circa 1.5 × as long as wide; last flagellomere as long as F1; marginal cell length 0.75 distance from apex of cell to wing tip; cu-v of hind wing equal to length of second abscissa of M+Cu; S5 with apicomedial emargination 3.3 × as wide as deep; S6 with apicomedial emargination circa three times as wide as deep, covered in dense black bristles anteriorly flanked by dense branched orange hairs that form a median patch.

*Genitalia*: penis valves with slightly extended shoulders; volsella with circa 12 setae (Fig. 20H); gonocoxa laterally with numerous small setae; apex of gonocoxa ventrally with small bilobed process (Fig. 20H); outer gonostylus club shaped with dense setae on inner surface; inner gonostylus circa as long as outer gonostylus, apex slightly widened, bearing strong setae (Fig. 20G); S7 (Fig. 20E); S8 apex wide, emarginated, edges rounded, fine setae at apex and along the midline (Fig. 20F).

***Pubescence***: Head white on labrum, mandibles, vertex and lower two-thirds of genae, some dark hairs below lateral ocelli, along eye margins and the clypeus; scutum, scutellum and metanotum light ochre brown with scattered longer dark hairs; mesosoma laterally and ventrally pale; fore leg with long orange hairs on outer surface; fore leg inner surfaces of tibia and tarsus with short dark brown to black hairs; mid femur with brown-black hairs, short and widespread on outer surface and longer on inner surface; mid tibia outer surface with orange hairs, inner surface almost bare, with long dark hairs; mid tarsus, hind leg outer surface of femur, tibia and metatarsus with long orange hairs; hind leg inner surface black; metasomal terga with orange erect branched hairs; T1 with narrow row of pale hairs on posterior margin; T2 anteriorly with erect black hairs, forming a black band about half the terga width; T7 orange on disk and two black patches at posterior corners; S1-S5 with fringes of pale hairs on posterior margins of S1 and S2; S2-S5 with lateral patches of long orange hair; S4 apicomedial area with broad, patch of strong simple black forward directed bristles, ½ - 2/3 of width of sternum (Fig. 20C); S5 with long dark branched hairs around the apicomedial emargination and sorter thin simple pale hairs on disc; S6 with dense dark branched hairs on disc and dense long branched orange hairs posteriorly, forming a dense median patch.

***Colouration***: Integument mostly black; metatarsi and tarsi orange; scape pale yellow below; labrum pale yellow with brown dots in dorsolateral corners; clypeus pale yellow; supraclypeal area pale yellow; paraclypeal area pale yellow; mandible basal 3/5 ivory remaining part brown and black; proboscis orange-brown.

**Female** (SAMA 32-033614, KR04604): Body length 17 mm, forewing length 11.2 mm, head width 5.6 mm.

***Structure***: Inner orbits of eyes slightly diverging above; head wider than long; clypeal protuberance in profile 0.87 × eye width; mandible with subapical tooth; F1 equal to combined length of next 3.5 flagellomeres; F1 1.2 × as long as scape; F2 0.79 × as long as F3; F3-F10 1.3 × as long as wide; last flagellomere 0.56 × as long as F1; marginal cell length 0.73 × distance from apex of cell to wing tip; cu-v of hind wing equal to length of second abscissa of M+Cu.

***Pubescence***: Head white on face, vertex and genae, some darker hairs scattered on face and on vertex; scutum, scutellum and metanotum with greyish and scattered longer dark hairs; mesosoma laterally and ventrally with pale to white hairs; fore leg femur and tibia with long white hair and tarsus with pale orange hairs on outer surface; fore leg inner surfaces of tibia and tarsus with short orange hair; mid femur basally with short pale orange hair, posteriorly short dense and white, ventrally long greyish black; mid tibia outer surface with orange hairs, inner surface almost bare, with streak of long dark hairs; mid metatarsus outer surface with orange hairs, inner with streak of black hairs; mid tarsus remaining segments with orange hairs; hind leg outer surface of femur, tibia and metatarsus with long orange hairs; hind leg inner surface black; metasomal terga: T1 anteriorly with long erect white hairs intermixed with a few black hairs, a small patch of brown hairs in the lateral corners, posterior margins with shorter adpressed pale yellow hairs; T2 anteriorly with entire band of erect brown hairs; T3-T6 with semi-adpressed pale orange hairs, which are slightly darker at posterior margins, intermixed with sparse erect long brown hairs; T5 with dense patch of brown hairs apicomedially, T6 with strong brown-black hairs flanking the pygidial plate; S1-S5 with rows of black hairs on inner posterior margins, lateral corners paler.

***Colouration***: Integument mostly black, posterior margins of terga, and tarsi orange; scape black, apically brown; labrum pale yellow with brown dots in dorsolateral corners; clypeus yellow with brown horseshoe like mark (Fig. 20M), supraclypeal area with yellow flat triangle; paraclypeal area black; mandible basal 3/5 ivory remaining part brown and black; proboscis orange-brown.

**Table:** **Phenology.**

**Month**:	**Jan**	**Feb**	**Mar**	**Apr**	**May**	**Jun**	**Jul**	**Aug**	**Sep**	**Oct**	**Nov**	**Dec**
No. of records:	0	0	0	0	3	8	17	48	24	2	1	0

#### Remarks.

Some field observations on mating behaviour and nests can be found on the following link: http://davotrip.blogspot.com/2009/08/interesting-inverts-burrowing-bees.html.

#### Flower records.

*Cassia charlesiana*, *C. luersenii*, *Senna glutinosa* subsp. *charlesiana, Petalostylis* sp. (Fabaceae), *Eremophila*, *E. gilesii*, *E. cf. georgei*, *E. maculata*, *E. longifolia* (Scrophulariaceae), *Goodenia maideniana* (Goodeniaceae), *Trichodesma zealanicum* (Boraginaceae), *Keraudrenia* sp. (Sterculiaceae), *Tecoma* sp. (Bignoniaceae).

#### Distribution.

Central Australia, the northern limit of the distributions is just north of the tropic of Capricorn, Figure 20I.

#### Etymology.

The specific epithet refers to the wide brush of stiff setae apicomedially on S4 of the male.

### Amegilla (Asaropoda) scymna

Taxon classificationAnimaliaHymenopteraApidae

(Gribodo, 1894)

7927D3B9-5C02-5089-BF15-449C758FA250

Anthophora scymna Gribodo, 1893: 389.

#### Type.

Holotype of *A. scymna*, female, Queensland Australia, Civic Museum of Natural History at Genoa (MSNG) /Gr (box 28) holotype ♀ “Australia / Queensland” (hw, G) “Anthophora / scymna / ♀ Grib / Tipo / D. Gribodo.” (hw, G) “revid. 1956 / M.A.Lieftinck” (pr + hw, L). References: Brooks 1988: 515, 572 (listed); Ruggiero 2009 (checklist); Ascher & Pickering 2014 (checklist). Remarks: in Ruggiero (2009) and Ascher and Pickering (2014) the date of description is erroneously reported as “1893” (Penati and Mariotti 2015).

#### Diagnosis.

Female paraclypeal and supraclypeal marks absent, tibial scopa pale orange, T2 without band of black hairs anteriorly. Scutum and terga pubescence uniformly ochre brown.

#### Remarks.

This specimen could not be matched with any other species, or any of the specimens examined. Based on the diagnostic characters mentioned above it would key out to *A. flava* (pg. 59 couplet 33), but the shape markings on the clypeus colouration and their distributions do not match.

### Amegilla (Asaropoda) xylocopoides

Taxon classificationAnimaliaHymenopteraApidae

Leijs, sp. nov.

CC6D41F3-331B-51CC-B6F3-1668D3CFB12B

http://zoobank.org/B9EADB16-AC8B-4051-890A-E7CBA8EED571

Figure 21A–O

#### Specimens examined.

(11 males, 1 female).

#### Types.

Holotype, male, 19 km SE of Laura, Qld (15.6705S; 144.5883E), 11 Mar.2017, R Leijs, QM T238476, DNA voucher RL2433b;

Allotype, female, Iron Range, Cape York Qld (12.72S; 143.30E), 1966, C O’Reilly, AM K470082;

Paratypes, 3 males, same locality data as holotype, QM T238475, SAMA KR06115, SAMA KR06118, 1 male 6.7 km NE of Laura, Qld (15.5011S; 144.4838E), 15 Mar. 2017, R Leijs, SAMA KR06205, 2 males 18 km SE of Laura, Qld (15.6609S; 144.5753E), 15 Mar.2017, R Leijs, SAMA KR06206, SAMA KR06207, 4 males, Cape York, Qld (15.5081S; 143.5081E), 4 Jun. 1985, NW Rodd, AM K470083-6.

#### Diagnosis.

Both sexes metasomal terga entirely with black pubescence.

#### Description.

Male holotype (QM T238476, RL2433b): Body length 16 mm, forewing length 12.7 mm, head width 5.6 mm.

***Structure***: Inner orbits of eyes diverging above; head wider than long; clypeal protuberance in profile 0.53 × eye width; mandible with weak subapical tooth; F1 equal to combined length of next 1.5 flagellomeres; F1 0.63 × as long as scape; F2 0.82 × as long as F3; F3-F10 1.3 × as long as wide; last flagellomere 1.14 × as long as F1, marginal cell length 0.81 × distance from apex of cell to wing tip; cu-v of hind wing 1.77 × length of second abscissa of M+Cu; S5 with apicomedial emargination very shallow, 6.3 × as wide as deep; S6 with apicomedial emargination circa four times as wide as deep.

**Figure 21.**
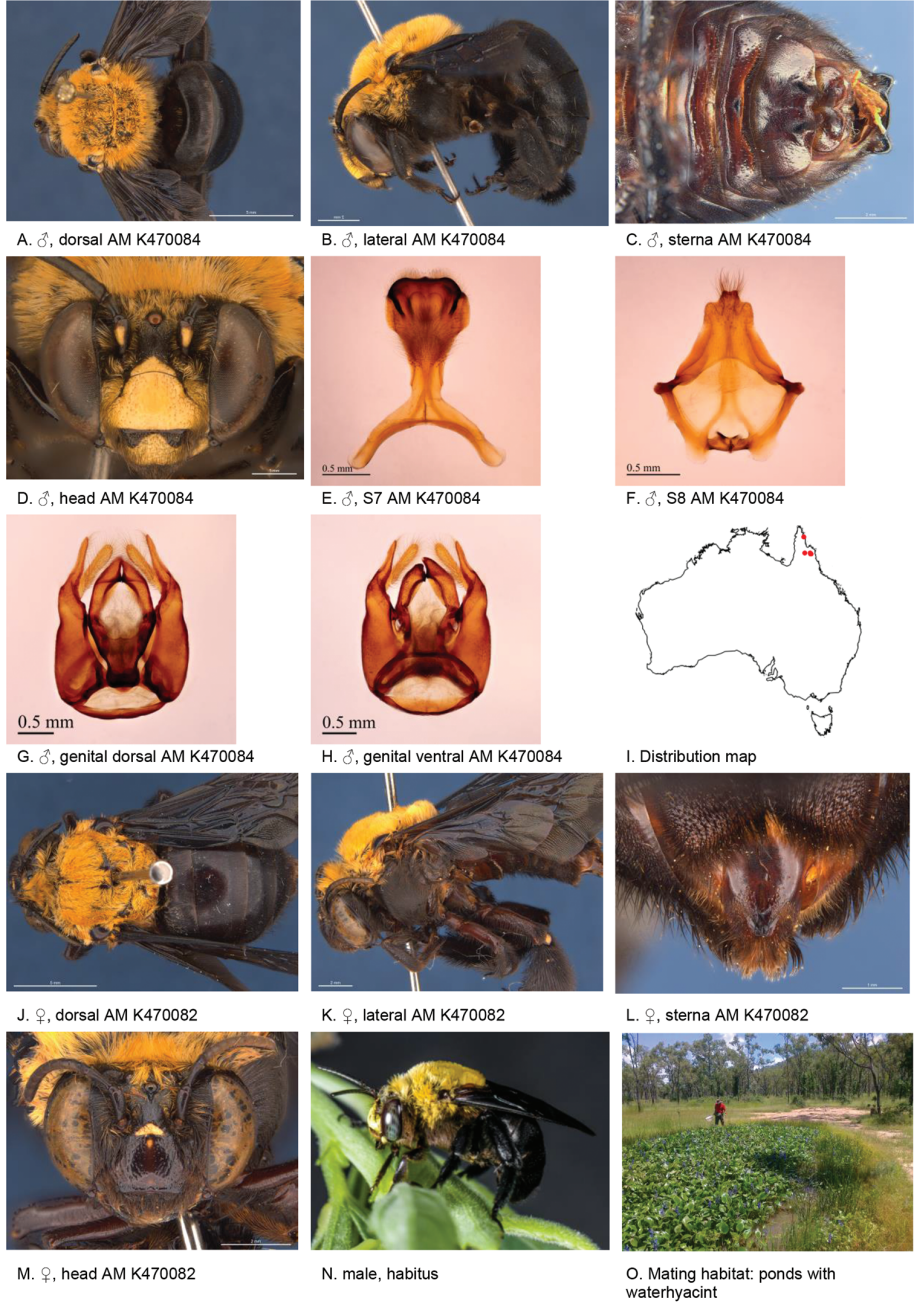
Amegilla (Asaropoda) xylocopoides Leijs, sp. nov.

*Genitalia*: penis valves shoulders not extended; volsella elongated, with 6 long setae (Fig. 21H); gonocoxa laterally with numerous small setae; apex of gonocoxa ventrally without process (Fig. 21H); outer gonostylus long and slender with fine setae on inner surface; inner gonostylus circa as long as outer gonostylus, but more robust, with numerous long setae (Fig. 21G); S7 (Fig. 21E); S8 apex almost tridentate middle process, emarginate with long setae (Fig. 21F).

***Pubescence***: Head black on labrum, clypeus and genae, orange brown around ocelli and vertex; scutum, scutellum, metanotum and metapostnotum orange-brown; mesosoma laterally and ventrally black; fore leg and metasoma with black hairs only; S4 apicomedial area with small patch of ventrally directed black bristles, S5 with two longitudinal semi-parallel patches of posteriorly directed bristles, S6 almost bare.

***Colouration***: Integument black, apart from: scape yellow patch below; labrum yellow with large black marks in dorsolateral corners; clypeus yellow; supraclypeal area with yellow triangle; paraclypeal area yellow; mandible yellow at base; proboscis dark brown; wings dark brown.

**Female** allotype (AM K470082): Body length 19 mm, forewing length 15.6 mm, head width 6.7 mm.

***Structure***: Inner orbits of eyes diverging above; head wider than long; clypeal protuberance in profile 0.75 × eye width; mandible with weak subapical tooth; F1 equal to combined length of next 2.7 flagellomeres; F1 0.88 × as long as scape; F2 circa as long as F3; F3-F10 slightly longer than wide; last flagellomere 0.57 × as long as F1; marginal cell length 0.82 × distance from apex of cell to wing tip; cu-v of hind wing 1.27 × length of second abscissa of M+Cu; S6 with distinct blunt posteriorly projecting subapical lobe, visible as spine when viewed laterally (Fig. 21L).

***Pubescence***: Head black on labrum, clypeus and genae, orange brown around ocelli and vertex; scutum, scutellum, metanotum and metapostnotum orange-brown; mesosoma laterally and ventrally with black hairs only; fore leg with legs and metasoma with black hairs only.

***Colouration***: Integument black to dark brown; legs and sterna brown; scape black; labrum black; clypeus dark brown with tiny yellow ventro- and dorso-median dots; supraclypeal area with small yellow triangle; paraclypeal area black; mandible with tiny yellow mark at base; proboscis dark brown; wings dark brown.

**Table:** **Phenology.**

**Month**:	**Jan**	**Feb**	**Mar**	**Apr**	**May**	**Jun**	**Jul**	**Aug**	**Sep**	**Oct**	**Nov**	**Dec**
No. of records:	0	0	7	0	0	4	0	0	0	0	0	0

#### Flower records.

At three different locations in 2017, male territorial behaviour was observed above water with native Water Hyacinth, *Monochoria australasica* (Pontederiaceae). Figure 21O.

#### Distribution.

Figure 21I.

#### Etymology.

The specific epithet refers to Australian female carpenter bees Xylocopa (Koptortosoma) sp., because of its superficial resemblance with respect to hair and wing colouration.

### Amegilla (Asaropoda) youngi

Taxon classificationAnimaliaHymenopteraApidae

Leijs, sp. nov.

734FC504-128A-5D9E-8551-A38F172C6135

http://zoobank.org/32B35BD9-E057-4EC0-AD62-BC2D4841E8CE

Figure 22A-E

#### Specimens examined.

(1 female):

#### Types.

Holotype, female, Litchfield, NT (13.4810S; 130.6990E), 18 Feb. 2006, DA Young., SAMA 32-033601, RL0707.

#### Diagnosis.

**Figure 22.**
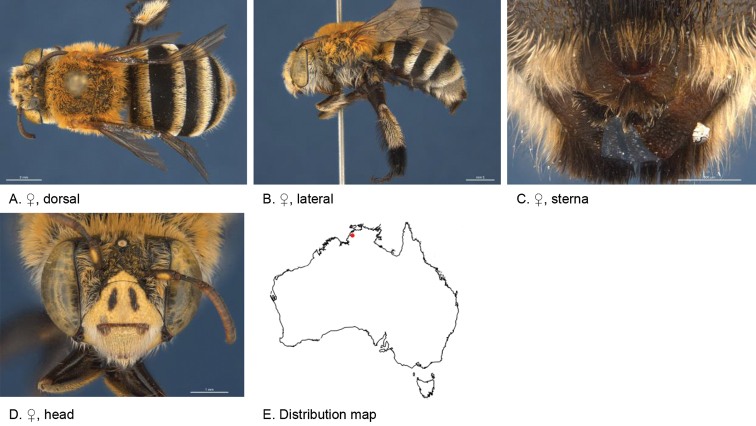
Amegilla (Asaropoda) youngi Leijs, sp. nov.

Male unknown. Female T2-T4 with black pubescence and pale posterior hairbands, T5 entirely with white hairs, clypeus with two black sub-parallel marks, process on S6 smooth with well-developed broad ridge (Fig. 22L).

#### Description.

Female holotype (SAMA 32-033601, RL707): Body length 13 mm, forewing length 9.1 mm, head width 4.5 mm.

***Structure***: Inner orbits of eyes diverging above; head wider than long; clypeal protuberance in profile 0.69 eye width; mandible with weak subapical tooth; F1 equal to combined length of next 3.1 flagellomeres; F1 0.9 × as long as scape; F2 circa as long as F3; F3-F10 slightly shorter than wide; last flagellomere 0.76 × as long as F1; marginal cell length 0.72 × distance from apex of cell to wing tip; cu-v of hind wing 0.89 × length of second abscissa of M+Cu; S6 with distinctly rounded posteriorly projecting subapical lobe, visible as spine when viewed laterally (Fig. 22C).

***Pubescence***: Head white on labrum, clypeus, scape and lower half of genae; pale orange hairs on upper genae and frons above antennal sockets; scutum, scutellum, metanotum, mesosoma laterally and ventrally under wing basis and pronotal lobe orange-brown, mesosoma white ventrally, small patch of black hair below hind wing; fore leg with long white hairs on posterior parts of femur and tibia, tarsi black; fore leg inner surfaces of tibia and tarsus with dark brown hairs; mid femur black; mid tibia black, outer surface white with small patches of pale orange hairs apically; mid metatarsus and tarsus black; hind leg outer surface of femur, tibia and metatarsus black, scopa and around basitibial plate white; hind leg inner surface black; metasomal terga with T1 pale orange and thin row of white hairs on posterior margin, T2–T4 black with white hairbands on posterior margins; T5 with white adpressed hairs and dense patch of black hairs apicomedially, T6 with strong brown hairs flanking the pygidial plate; S1-S5 with short dark hairs and rows of white hairs which are longer at lateral corners.

***Colouration***: Integument black, apart from: antennae brown below; S1 brown; scape with pale yellow patch below; labrum ivory with two small brown marks in dorsolateral corners; clypeus ivory, with two black sub-parallel marks; supraclypeal area with ivory triangle; paraclypeal area black, mandible ivory at base, brown at tip; proboscis brown.

**Table:** **Phenology.**

**Month**:	**Jan**	**Feb**	**Mar**	**Apr**	**May**	**Jun**	**Jul**	**Aug**	**Sep**	**Oct**	**Nov**	**Dec**
No. of records:	0	1	0	0	0	0	0	0	0	0	0	0

#### Flower records.

None.

#### Distribution.

Figure 22E.

#### Etymology.

The specific epithet refers to Andy Young, the collector of the specimen, in honour of his contribution to *Amegilla* taxonomy by providing fresh specimens in ethanol, which were valuable for DNA analyses.

## Supplementary Material

XML Treatment for Subgenus Asaropoda

XML Treatment for Amegilla (Asaropoda) albiceps

XML Treatment for Amegilla (Asaropoda) albiclypeata

XML Treatment for Amegilla (Asaropoda) albigenella

XML Treatment for Amegilla (Asaropoda) aurantia

XML Treatment for Amegilla (Asaropoda) batleyi

XML Treatment for Amegilla (Asaropoda) bombiformis

XML Treatment for Amegilla (Asaropoda) calva

XML Treatment for Amegilla (Asaropoda) crenata

XML Treatment for Amegilla (Asaropoda) dawsoni

XML Treatment for Amegilla (Asaropoda) epaphrodita

XML Treatment for Amegilla (Asaropoda) flava

XML Treatment for Amegilla (Asaropoda) frogatti

XML Treatment for Amegilla (Asaropoda) griseocincta

XML Treatment for Amegilla (Asaropoda) houstoni

XML Treatment for Amegilla (Asaropoda) incognita

XML Treatment for Amegilla (Asaropoda) nitidiventris

XML Treatment for Amegilla (Asaropoda) preissi

XML Treatment for Amegilla (Asaropoda) rhodoscymna

XML Treatment for Amegilla (Asaropoda) scoparia

XML Treatment for Amegilla (Asaropoda) scymna

XML Treatment for Amegilla (Asaropoda) xylocopoides

XML Treatment for Amegilla (Asaropoda) youngi

## Supplementary materials

Supplementary material 1 Supplementary table - examined specimen data Data type: Microsoft Excell Spreadsheet (.xlsx). Explanation note: Examined specimen data. https://binary.pensoft.net/file/376139This dataset is made available under the Open Database License (http://opendatacommons.org/licenses/odbl/1.0/). The Open Database License (ODbL) is a license agreement intended to allow users to freely share, modify, and use this Dataset while maintaining this same freedom for others, provided that the original source and author(s) are credited.Remko Leijs, James Dorey, Katja Hogendoorn
